# Matching the Coupling of Valence Electrons in the Oxide Interface to Perturb the Magnetic Order Enhancing Oxygen Reduction in Zinc–Air Batteries

**DOI:** 10.1002/anie.7852726

**Published:** 2026-05-25

**Authors:** Jing Li, Ningkang Peng, Jianhua Ma, Tingyu Lu, Haibin Zhu, Guangyao Zhou, Yizhou Zhang, Yanhui Gu, Yawen Tang, Hao Li

**Affiliations:** ^1^ School of Chemistry and Chemical Engineering Southeast University Nanjing China; ^2^ Advanced Institute for Materials Research (WPI‐AIMR) Tohoku University Sendai Japan; ^3^ College of Science Jinling Institute of Technology Nanjing China; ^4^ School of Chemistry and Materials Science, Jiangsu Key Laboratory of New Power Batteries, Jiangsu Collaborative Innovation Center of Biomedical Functional Materials Nanjing Normal University Nanjing China; ^5^ School of Computer and Electronic Information Nanjing Normal University Nanjing China

**Keywords:** electronic compatibility, oxygen reduction reaction, spin state regulation, super‐exchange interaction, zinc–air battery

## Abstract

The inherently locked spin state between the metal sites and oxygen‐containing intermediates imposes an intrinsic limitation on the maximum achievable oxygen reduction reaction (ORR) activity. Herein, we construct the sub‐5 nm Fe_2_O_3_/Sm_2_O_3_ heterojunctions immobilized in N‐doped carbon nanofibers (denoted as sub‐5 nm Fe_2_O_3_/Sm_2_O_3_@N‐CNFs), where coupled Fe (3d)‐O (2p)‐Sm (4f) orbitals can regulate the interfacial spin order, thereby attenuating the Fe–OH binding. *Operando* spectroscopy and density functional theory calculations reveal that the super‐exchange interaction across the Fe–O–Sm bond induces an antiparallel magnetic alignment, which suppresses the spin interaction with OH* at the surface, thereby accelerating OH* desorption and enhancing ORR activity. In 0.1 M KOH, the catalyst delivers excellent ORR performance with a half‐wave potential of 0.94 V and a Tafel slope of 92.4 mV dec^−1^, along with long‐term stability. Furthermore, the liquid‐ and all‐solid‐state rechargeable zinc–air batteries (ZABs) assembled with sub‐5 nm Fe_2_O_3_/Sm_2_O_3_@N‐CNFs also exhibit marked device performance, surpassing Pt/C + RuO_2_ benchmarks. These results demonstrate that interfacial spin regulation via Fe–O–Sm coupling is an effective strategy to enhance ORR activity and stability of catalysts by reconfiguring local magnetic ordering to tune oxygenated‐intermediate adsorption. More broadly, this intrinsic‐property modulation can be extended to other anion‐bridged compounds and spin‐involved electrocatalytic reactions.

## Graphical abstract

Sub‐5 nm Fe_2_O_3_/Sm_2_O_3_ heterojunction nanoparticles are anchored on nitrogen‐doped carbon nanofibers, featuring coupled Fe(3d)‐O(2p)‐Sm(4f) orbitals at the interface. The resulting super‐exchange induces antiparallel magnetic ordering at heterointerfaces, suppressing spin‐dependent interaction between surface OH* species and Fe sites to facilitate OH* desorption. Therefore, the catalyst exhibits excellent oxygen reduction reaction performance.

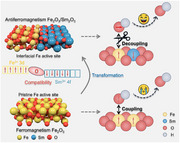

## 1 Introduction

Oxygen reduction reaction (ORR) is a fundamental electrochemical process central to the performance of energy conversion technologies such as fuel cells and metal‐air batteries [1]. However, ORR suffers from inherently sluggish kinetics and high overpotential arising from complex proton‐coupled electron transfer (PCET), which limits system efficiency [2]. Therefore, the development of electrocatalysts with optimized adsorption‐desorption behavior is essential to overcome kinetic and thermodynamic barriers to enhance ORR performance [3]. State‐of‐the‐art Pt‐based catalysts have been considered the benchmark due to their excellent activity nearing the apex of the ORR volcano plot [4]. Nevertheless, their widespread deployment is hindered by unacceptable cost, natural scarcity, and susceptibility to poisoning [5]. These limitations have stimulated extensive efforts to design earth‐abundant electrocatalysts with superior activity, stability, and cost‐effectiveness.

Among numerous candidates, Fe‐based materials are promising because their ORR activity approaches the performance of Pt‐based benchmarks at a lower cost [6]. Within this family, compared with Fe single‐atom catalysts and other Fe‐based compounds, Fe oxides (Fe_2_O_3_) exhibit a highly stable oxide lattice, which can enhance chemical stability against active site degradation under alkaline conditions [7, 8]. Furthermore, surface Fe atoms of Fe_2_O_3_ exhibit natural unsaturated oxygen coordination and intrinsic ferromagnetism, which can provide strong adsorption for oxygen intermediates to trigger the reaction [9, 10]. Unfortunately, this strong interaction also leads to excessive OH* adsorption, which blocks active sites to raise the reaction barrier, thus ultimately setting OH* reduction as the rate‐determining step of the entire system [11]. Specifically, this result arises from ferromagnetic ordering at the Fe_2_O_3_ surface, which causes spin‐selective stabilization of adsorbed OH*, thus strengthening Fe–OH interactions and slowing desorption [12]. Therefore, to address this issue, targeted modulation of the electronic and magnetic properties of Fe_2_O_3_ is significant to weaken OH* binding to accelerate the cycling efficiency of active sites, thereby unlocking the catalytic potential of Fe_2_O_3_. Recent advances indicate that tuning the electronic spin states of catalysts can regulate spin‐dependent interactions with spin‐polarized intermediates (e.g., OH*), thereby optimizing the energetics of key intermediates and accelerating ORR kinetics [13, 14, 15, 16]. In addition, the heterojunction strategy is considered to have the advantage of maintaining the lattice integrity of Fe_2_O_3_ while still keeping strong interactions at the heterogeneous interface [17]. Therefore, the formation of a heterogeneous interface can generate the interfacial exchange coupling to tune spin alignment and local electronic configuration at the nanoscale, thereby achieving the coexistence of activity and stability [18, 19]. Rare earth (RE) oxides are an excellent choice due to their excellent lattice compatibility with ferrites, high electrical conductivity contribution, and unique intra‐atomic 4f valence‐electron exchange properties [20, 21]. Among them, Sm_2_O_3_ exhibits unique magnetic characteristics because Sm^3+^ has a partially filled high‐spin 4f configuration (4f^5^6s^0^). Notably, its open‐shell electronic structure is analogous to that of high‐spin Fe^3+^ (3d^5^4s^0^), such that both ions carry robust localized magnetic moments. Building on this valence‐electron compatibility, enhanced interfacial spin polarization is expected to render antiparallel exchange across Sm–O–Fe linkages energetically accessible via f–p–d orbital coupling, thereby perturbing the native magnetic ordering of Fe_2_O_3_ and facilitating spin injection and interfacial orbital hybridization [22, 23, 24]. Moreover, in contrast to other rare earth oxides, Sm_2_O_3_ exhibits typical cubic lattice parameters (∼9.5 Å), which are well‐matched with those of cubic Fe_2_O_3_ (∼8.5 Å) [25, 26]. This structural compatibility can promote the formation of strongly interacted heterojunction interfaces characterized by low defect densities and thermodynamic stability, thereby enhancing interfacial electronic‐spin coupling and charge transfer to boost the catalytic activity [27]. Consequently, Sm_2_O_3_ is chosen as an ideal modulator for Fe_2_O_3_ because it can simultaneously tune interfacial magnetism and stabilize the heterostructure. The resulting Fe–O–Sm interaction can disrupt interfacial long‐range ferromagnetic ordering and weaken the spin‐dependent coupling between OH* and site, which accelerates ORR kinetics [28].

Based on the background mentioned above, we herein propose to construct the ultra‐small Fe_2_O_3_/Sm_2_O_3_ heterojunction nanoparticles anchored in N‐doped carbon nanofibers (abbreviated as sub‐5 nm Fe_2_O_3_/Sm_2_O_3_@N‐CNFs hereafter). This heterostructure can form a gradient orbital coupling unit [Fe (3d)‐O (2p)‐Sm (4f)], which allows for the regulation of the spin‐ordering degree at Fe sites to facilitate OH* desorption. Experimental and theoretical investigations demonstrate that the interfacial Fe–O–Sm coupling can construct a rapid electron transport pathway, which facilitates the electron supply to adsorbed OH* to reduce the desorption energy barrier. Concurrently, the heterointerface disrupts surface ferromagnetic ordering, suppressing spin‐induced strong coupling of OH* at the surface and enhancing the overall catalytic dynamics. The regulations of electronic structure and magnetism properties cooperatively accelerate the OH* reduction step, thereby both enhancing the intrinsic ORR activity and preserving structural robustness. As a result, the as‐obtained sub‐5 nm Fe_2_O_3_/Sm_2_O_3_@N‐CNFs exhibits remarkable ORR performance in a 0.1 M KOH electrolyte, including the higher half‐wave (0.94 V_RHE_), faster reaction kinetics (92.4 mV dec^−1^), and long‐term durability, outperforming commercial Pt/C catalysts and most as‐reported Fe‐based catalysts. When integrated into rechargeable zinc–air batteries (ZABs) as the air cathode, the sub‐5 nm Fe_2_O_3_/Sm_2_O_3_@N‐CNFs also possess excellent performance in liquid and all‐solid‐state configurations, surpassing that of commercial Pt/C + RuO_2_ systems. This study establishes a clear design principle for constructing spin‐regulated heterojunction catalysts and provides valuable insights for the development of high‐performance ORR electrocatalysts toward next‐generation energy devices.

## 2 Results and Discussion

### 2.1 Design Rationale

**FIGURE 1:**
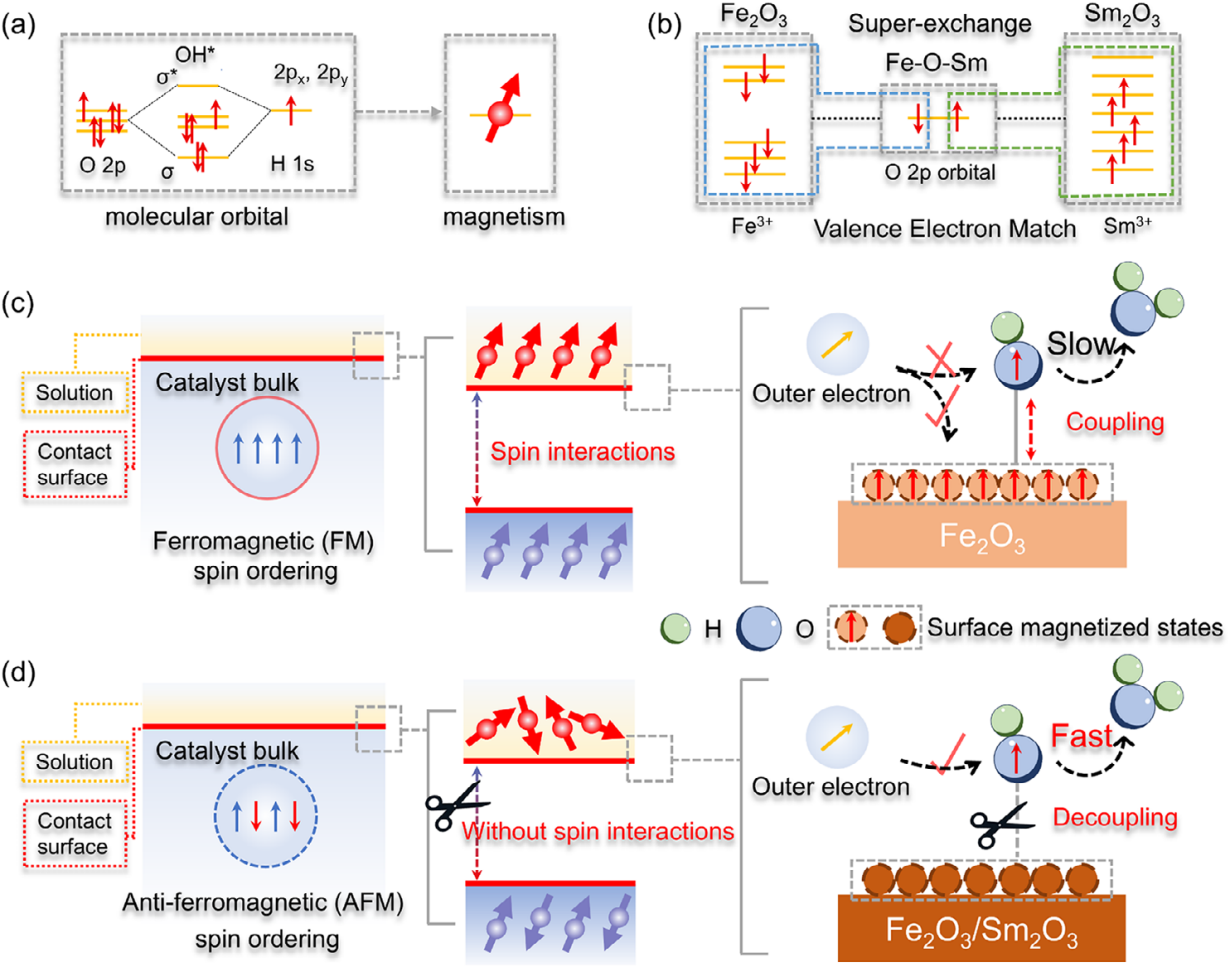
Spin‐state regulation at Fe_2_O_3_/Sm_2_O_3_ interface to promote OH* desorption in ORR: (a) schematic of the OH* molecular orbital configuration and its associated magnetic moment; (b) antiferromagnetic alignment induced by super‐exchange coupling of the [Fe–O–Sm] unit; (c) parallel spin alignment induces OH* trapping at the contact surface via spin‐interactions, inhibiting electron transfer and resulting in sluggish desorption; and (d) antiferromagnetic alignment at the Fe_2_O_3_/Sm_2_O_3_ heterointerface reduces spin‐interaction, enhances electron accessibility, and facilitates OH* reduction.

Typically, ORR proceeds via a four‐electron process involving O_2_ adsorption, OOH* formation, O–O bond cleavage, OH* generation, and desorption as H_2_O. Among these, O_2_ adsorption and activation initiate the reaction, and OH* desorption sustains catalytic turnover, both of which may critically govern the overall rate [29]. For Fe_2_O_3_‐based materials, the conversion of OH* to H_2_O constitutes the RDS rather than initial O_2_ activation [11]. This originates from the favorable orbital overlap between Fe 3d orbitals and O_2_ π* orbitals at the surface, which promotes O_2_ adsorption and activation. In contrast, the desorption of OH* is thermodynamically less favorable due to its higher free energy barrier, particularly under strong adsorption regimes, which blocks the sites and slows overall reaction kinetics [30]. As shown in Figure 1a, the OH* is a spin‐active odd‐electron intermediate, which selectively couples with the intrinsic ferromagnetic ordering of Fe_2_O_3_. This spin‐selective coupling can give rise to spin interactions, effectively trapping OH* on the surface and increasing the energy barrier of desorption [28]. To overcome this limitation, it is essential to achieve spin‐state decoupling between OH* and the Fe active sites, while simultaneously facilitating rapid electron injection into absorbed OH* and accelerating H_2_O formation. Herein, we propose a heterointerface engineering strategy to address spin‐dependent OH* adsorption, thereby enabling a more favorable energetic pathway for ORR on the Fe_2_O_3_ surface. This strategy co‐modulates interfacial electron spin state and electronic structure by constructing Fe–O–Sm active units. Within this configuration, oxygen atoms serve as electronic bridges to mediate coupling of valence‐electron‐matching Fe (3d) and Sm (4f) orbitals [22]. As shown in Figure 1b, the resulting super‐exchange interactions in Fe–O–Sm units promote antiparallel spin alignment between Fe and Sm centers, which can disrupt the long‐range ferromagnetic order at the pristine Fe_2_O_3_ interface [31, 32]. In greater depth, as conventional ferromagnetic oxides, the pristine Fe_2_O_3_ exhibits the collective spin alignment of internal electrons, giving rise to a pronounced interfacial magnetic ordering at the contact surface, thus resulting in spin‐polarized adsorption of paramagnetic OH* species (Figure 1c) [33]. This magnetic ordering‐induced spin interaction strengthens the coupling between OH* and Fe sites, thereby increasing the desorption barrier. Furthermore, ordered spin domains impede the mobility of charge carriers by localizing electronic states, thus suppressing efficient electron transfer toward adsorbed OH*. In contrast, as shown in Figure 1d, the Fe_2_O_3_/Sm_2_O_3_ heterointerface utilizes Sm‐derived 4f magnetic states to counter‐align with Fe magnetic moments, establishing an antiferromagnetic configuration. This perturbation reduces the surface long‐range order spin polarization, thereby weakening the magnetic interaction between OH* and the catalytic sites to promote its decoupling. In addition, the resulting spin‐decoherent surface environment enhances electron delocalization and mobility, thereby facilitating electron delivery to OH* for subsequent H_2_O formation. Therefore, the synergistic integration of orbital coupling and spin regulation effectively attenuates the strong interaction between OH intermediates and sites, significantly enhancing the intrinsic catalytic activity of ORR. This design rationale is rooted in the spin‐dependent mechanism of ORR, which highlights the potential of interfacial spin engineering to develop high‐performance and stable non‐precious metal oxide catalysts.

### 2.2 Materials Synthesis and Characterization

**FIGURE 2:**
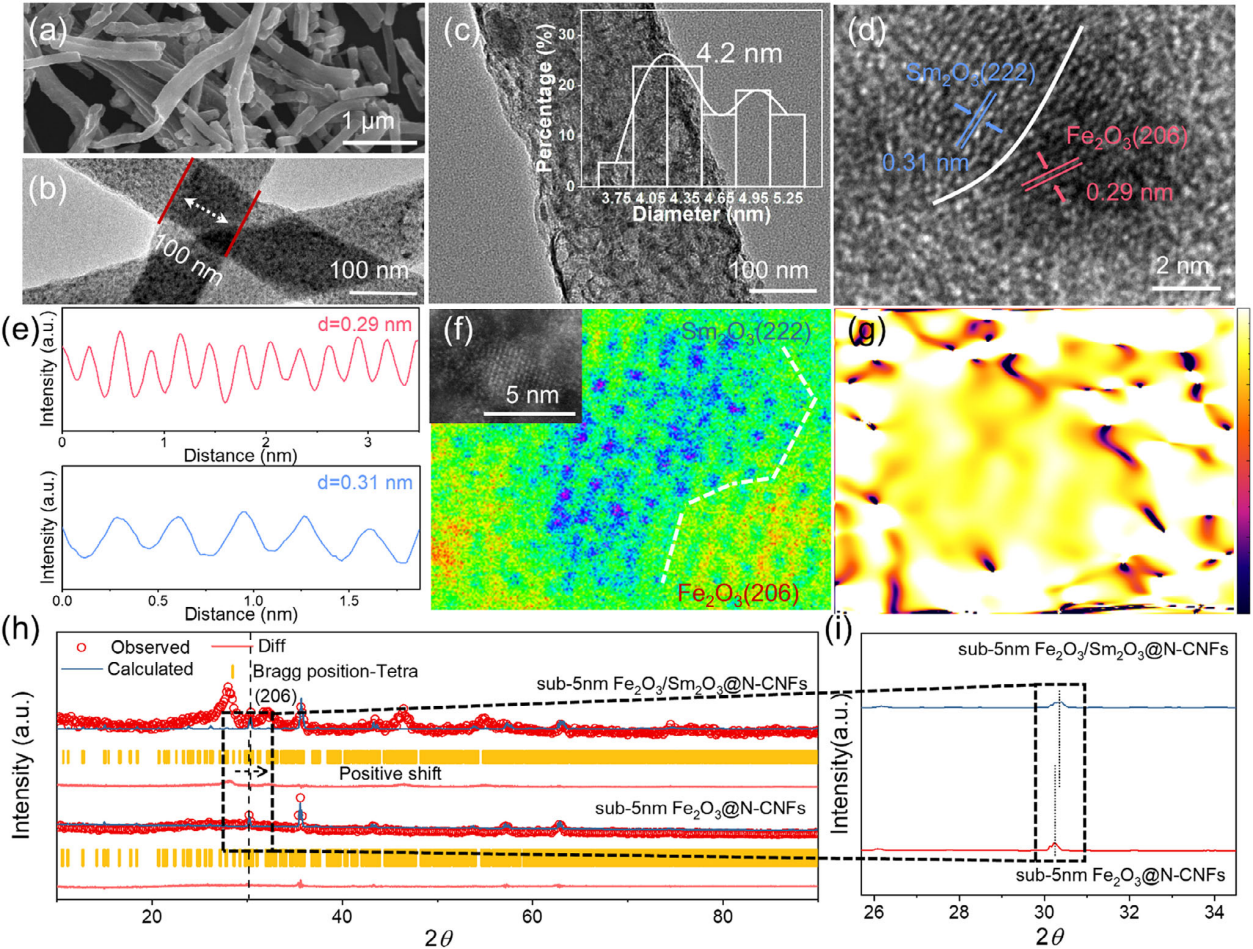
Structural elucidation of the sub‐5 nm Fe_2_O_3_/Sm_2_O_3_@N‐CNFs: (a) SEM image, (b) TEM image, (c) HRTEM image, (d) HADDF‐STEM image, (e) lattice spacing, (f) the inverse FFT image obtained by selecting the FFT patterns in AC‐HADDF‐STEM image (inset: AC‐HADDF‐STEM image), and (g) GPA at *ε*_xx_. The crystal structure analysis sub‐5 nm Fe_2_O_3_/Sm_2_O_3_@N‐CNFs and sub‐5 nm Fe_2_O_3_@N‐CNFs: (h) Rietveld‐refined XRD patterns and (i) magnified image of diffraction peak variation.

Guided by theoretical insights, the rare‐earth element Sm (4f^6^6s2) with a unique electronic configuration is selected to modulate the Fe 3d orbitals, aiming to enhance ORR performance. The detailed synthesis procedure is shown in Figure S1. Briefly, through facile electrospinning and high‐temperature pyrolysis, ultrasmall Fe_2_O_3_/Sm_2_O_3_ heterojunction nanoparticles are anchored in one‐dimensional porous nitrogen‐doped carbon nanofibers (denoted as sub‐5 nm Fe_2_O_3_/Sm_2_O_3_@N‐CNFs), where terephthalic acid (H_2_BDC) serves as the chelating agent and polyacrylonitrile (PAN) provides nitrogen and carbon sources. Meanwhile, a series of control samples is produced by the same synthetic method to elucidate interaction mechanisms during material fabrication. Specifically, in the presence of ligands, the introduction of Fe^3+^ or Sm^3+^ ions yields the ultrasmall Fe_2_O_3_ nanoparticles anchored in N‐CNFs (denoted as sub‐5 nm Fe_2_O_3_@N‐CNFs) or ultrasmall Sm_2_O_3_ nanoparticles anchored in N‐CNFs (denoted as sub‐5 nm Sm_2_O_3_@N‐CNFs), respectively. In the absence of ligands, the introduction of Fe^3+^, Sm^3+^, or Fe^3+^ and Sm^3+^ ions leads to the formation of the corresponding Fe_2_O_3_@N‐CNFs, Sm_2_O_3_@N‐CNFs, or Fe_2_O_3_/Sm_2_O_3_@N‐CNFs, respectively. As shown in Figure S2a, the x‐ray diffraction (XRD) analysis results reveal that the synthesized sub‐5 nm Fe_2_O_3_/Sm_2_O_3_@N‐CNFs contains both Fe_2_O_3_ and Sm_2_O_3_ phases, corresponding to the tetragonal *P*4_3_2_1_2 space group (No. 96, PDF: 25–1402) and the cubic *I*a $\bar{3}\;$ space group (No. 206, PDF: 42–1461), respectively. Moreover, the obtained Fe_2_O_3_/Sm_2_O_3_@N‐CNFs (Figure S2d) exhibit a crystal structure identical to that of sub‐5 nm Fe_2_O_3_/Sm_2_O_3_@N‐CNFs, indicating that ligand incorporation only affects the nanoparticle size without altering the phase composition. This conclusion is further supported by XRD analyses of other comparative catalysts, such as sub‐5 nm Fe_2_O_3_@N‐CNFs (Figure S2b) and Fe_2_O_3_@N‐CNFs (Figure S2e), as well as sub‐5 nm Sm_2_O_3_@N‐CNFs (Figure S2c) and Sm_2_O_3_@N‐CNFs (Figure S2f). Scanning electron microscopy (SEM) (Figure 2a) and transmission electron microscopy (TEM) images (Figure 2b and Figure S3) exhibit that compared with other N‐CNFs family catalysts without the H_2_BDC (Fe_2_O_3_/Sm_2_O_3_@N‐CNFs, Fe_2_O_3_@N‐CNFs, and Sm_2_O_3_@N‐CNFs), the obtained catalysts with ligands present a more uniform and slender fibrous structure. This can be attributed to the strong coordination of H_2_BDC with metal ions, which promotes the homogeneous dispersion of the ions, thereby weakening the physical entanglement and coordination between the metal ions and the PAN polymer chains and enhancing the spinning quality [34]. Under the confinement effect of the ligand, sub‐5 nm Fe_2_O_3_/Sm_2_O_3_@N‐CNFs can retain a uniform and well‐defined one‐dimensional nanofiber morphology (diameter ≈100 nm) after carbonization, thereby endowing the potential for superior stability during catalysis. Furthermore, Figure 2c and Figure S4 show that H_2_BDC incorporation markedly reduces particle sizes from ∼17.3 nm (Fe_2_O_3_@N‐CNFs), ∼15.5 nm (Sm_2_O_3_@N‐CNFs), and ∼24.8 nm (Fe_2_O_3_/Sm_2_O_3_@N‐CNFs) to ∼1.9 nm (sub‐5 nm Fe_2_O_3_@N‐CNFs), ∼3.7 nm (sub‐5 nm Sm_2_O_3_@N‐CNFs), and ∼4.2 nm (sub‐5 nm Fe_2_O_3_/Sm_2_O_3_@N‐CNFs). This verifies the key role of strong electrostatic interactions between H_2_BDC and metal ions (Fe^3+^ and Sm^3+^) in suppressing spontaneous aggregation of nanoparticles at high temperature, thereby promoting the exposure of active sites for efficient ORR catalysis. Remarkably, as shown in Figure S5a, the as‐prepared sub‐5 nm Fe_2_O_3_/Sm_2_O_3_@N‐CNF exhibits the highest degree of carbon defects (*I*_D_/*I*_G_ = 1.21), compared to the sub‐5 nm Fe_2_O_3_@N‐CNFs (*I*_D_/*I*_G_ = 1.14), sub‐5 nm Sm_2_O_3_@N‐CNFs (*I*_D_/*I*_G_ = 1.11), Fe_2_O_3_/Sm_2_O_3_@N‐CNFs (*I*_D_/*I*_G_ = 1.08), Fe_2_O_3_@N‐CNFs (*I*_D_/*I*_G_ = 1.06), and Sm_2_O_3_@N‐CNFs (*I*_D_/*I*_G_ = 0.99). This result is attributed to the increment of metal content and reduction of particle size, which distinctly enhances the interaction between metal oxides and the nitrogen‐doped carbon matrix, thus disrupting the continuity of the sp^2^ carbon network and leading to a substantial increase in defect density [35]. Figure S5b,c illustrate that the N_2_ adsorption‐desorption isotherms of sub‐5 nm Fe_2_O_3_/Sm_2_O_3_@N‐CNF can be affiliated with a characteristic Type IV isotherm with an H4 hysteresis loop, revealing a significant presence of mesopore features, which is attributed to gas release during the high‐temperature decomposition of oxygen‐containing functional groups. Additionally, based on the results, the Brunauer–Emmett–Teller (BET) specific surface area is calculated to be 397.1 m^2^ g^−1^. It is worth noting that the abundant porous structure is conducive to mass transfer and the accessibility of active sites. The aberration‐corrected high‐angle annular dark‐field scanning transmission electron microscopy (AC‐HAADF‐STEM) image (Figure S6a) and energy‐dispersive x‐ray spectroscopy (EDS) elemental mappings (Figure S6b) collectively demonstrate a uniform distribution of ultrafine Fe_2_O_3_/Sm_2_O_3_ nanoparticles in the carbon substrate. The high‐resolution HAADF‐STEM image (Figure 2d) of the sub‐5 nm Fe_2_O_3_/Sm_2_O_3_@N‐CNFs shows a distinct heterogeneous interface composed of Fe_2_O_3_ (206) and Sm_2_O_3_ (222) planes, with lattice fringe spacings of 0.29 and 0.31 nm (Figure 2e), respectively, confirming the successful formation of the heterostructure, consistent with the XRD results above.

The geometric phase analysis (GPA) is performed to further investigate the lattice configuration arising from sub‐nanometer heterointerface coupling. As shown in Figure 2f,g, the *ε*_xx_ strain map of sub‐5 nm Fe_2_O_3_/Sm_2_O_3_@N‐CNFs reveals a pronounced lattice deformation localized at the coupling interface. This phenomenon is plausibly driven by the interfacial constraint due to the lattice mismatch between metastable tetragonal Fe_2_O_3_ and stable cubic Sm_2_O_3_, which distorts the Fe_2_O_3_ lattice to alter the average Fe–O bond length, thereby demonstrating strong interfacial coupling between Fe_2_O_3_ and Sm_2_O_3_. Rietveld refinement analysis (Figure 2h,i) further validates the microstructural phase transformation, where the Fe_2_O_3_ in sub‐5 nm Fe_2_O_3_/Sm_2_O_3_@N‐CNFs and sub‐5 nm Fe_2_O_3_@N‐CNFs samples both maintain a pure tetragonal phase. Exclusively, compared to pristine sub‐5 nm Fe_2_O_3_@N‐CNFs, the characteristic peaks of Fe_2_O_3_ in sub‐5 nm Fe_2_O_3_/Sm_2_O_3_@N‐CNFs exhibit a positive shift, attributed to the strong coupling at the heterogeneous interface, which induces contraction of the Fe_2_O_3_ phase structure to reduce interatomic spacing. These findings indicate that the Sm_2_O_3_ unit largely retains an O_h_‐like coordination symmetry, whereas the Fe_2_O_3_ domains adopt a tetragonally distorted Fe–O polyhedron with D_4_ symmetry (a subgroup of O_h_), arising from strong metal‐ligand coordination and interface contact. The resulting symmetry/coordination compatibility across the interface is expected to lower interfacial distortion energy, thereby facilitating robust Fe_2_O_3_/Sm_2_O_3_ coupling and promoting Fe (3d)‐O (2p)‐Sm (4f) orbital hybridization, which ultimately perturbs the Fe spin configuration and accelerates ORR kinetics.

**FIGURE 3:**
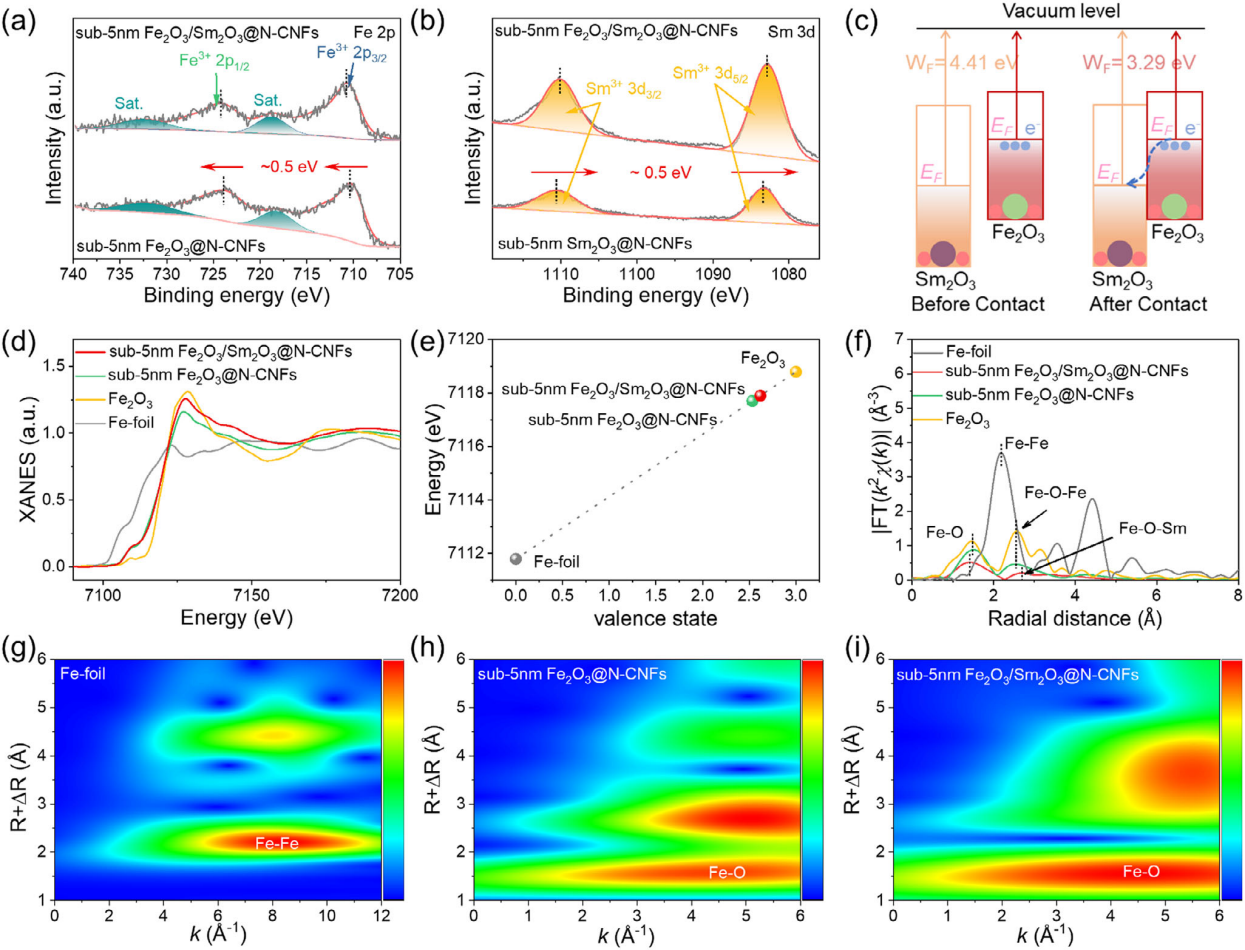
The analysis of valence state and bonding structure of elements in sub‐5 nm Fe_2_O_3_/Sm_2_O_3_@N‐CNFs and sub‐5 nm Fe_2_O_3_@N‐CNFs: (a) Fe 2p spectra, (b) Sm 3d spectra, (c) change in W_F_, (d) Fe K‐edge XANES spectra, (e) the valence state of Fe, (f) Fe K‐edge Fourier transform EXAFS spectra, and (g–i) wavelet transform for the *k* ^2^‐weighted EXAFS contour plots of the Fe element.

X‐ray photoelectron spectroscopy (XPS) measurement is conducted to unveil the chemical composition, bond configuration, and valence states of elements in sub‐5 nm Fe_2_O_3_/Sm_2_O_3_@N‐CNFs, sub‐5 nm Fe_2_O_3_@N‐CNFs, and sub‐5 nm Sm_2_O_3_@N‐CNFs. As shown in Figure S7, the XPS survey spectrums again confirm the presence of C, N, O, Sm, and Fe elements in sub‐5 nm Fe_2_O_3_/Sm_2_O_3_@N‐CNFs (Figure S7a), as well as C, N, O, and Fe elements in sub‐5 nm Fe_2_O_3_@N‐CNFs (Figure S7b) and C, N, O, and Sm elements in sub‐5 nm Sm_2_O_3_@N‐CNFs (Figure S7c). Additionally, as displayed in Figure S8a–c, the C 1s components of the three catalysts are located at 284.8 eV (C–C), ∼286.0 eV (C–N), and ∼287.0 eV (C–O), demonstrating similar carbon environments across the samples. Similarly, the N 1s spectra (Figure S8d–f) can be well fitted to four peaks located at ∼398.5, ∼400.5, ∼401.0, and ∼403.0 eV, corresponding to pyridine‐N, pyrrole‐N, graphitic‐N, and oxidized‐N, respectively. Meanwhile, the O 1s peaks (Figure S8g–i) also maintain a highly similar coordination environment at the corresponding C–O (∼531.5 eV) and adsorbed O (∼533.5 eV). In addition, similar to the Fe–O bond in sub‐5 nm Fe_2_O_3_@N‐CNFs (530.2 eV) and Sm–O bond in sub‐5 nm Sm_2_O_3_@N‐CNFs (529.8 eV), the sub‐5 nm Fe_2_O_3_/Sm_2_O_3_@N‐CNFs exhibits the consistent Fe–O and Sm–O bond. These XPS results indicate that the formation of heterojunction can generate mixed phases without changing the original chemical bond composition. For the high‐resolution Fe 2p spectrum (Figure 3a), a pair of dominating peaks led by Fe 2p spin‐orbit splitting is located at approximately 710.75 eV (Fe^3+^ 2p_3/2_) and 724.15 eV (Fe^3+^ 2p_1/2_), along with two satellite peaks at 718.76 and 732.45 eV in sub‐5 nm Fe_2_O_3_/Sm_2_O_3_@N‐CNFs [36]. The high‐resolution Sm 3d spectrum of sub‐5 nm Fe_2_O_3_/Sm_2_O_3_@N‐CNFs (Figure 3b) can be well deconvoluted into two prominent peaks located at 1082.83 and 1110.08 eV, corresponding to Sm^3+^ 3d_5/2_ and Sm^3+^ 3d_3/2_, respectively, arising from the spin‐orbital splitting effect [37]. Notably, identified Fe^3+^ characteristic peaks in sub‐5 nm Fe_2_O_3_/Sm_2_O_3_@N‐CNFs regularly positively shift about 0.5 eV compared with those of sub‐5 nm Fe_2_O_3_@N‐CNFs. In contrast, compared to the counterpart of pristine sub‐5 nm Sm_2_O_3_@N‐CNFs, the binding energies of Sm^3+^ characteristic peaks in sub‐5 nm Fe_2_O_3_/Sm_2_O_3_@N‐CNFs display a negative shift of ∼0.5 eV. These results collectively indicate the obvious electron transfer across heterogeneous interfaces, where Fe^3+^ acts as an electron donor and Sm^3+^ acts as an electron acceptor. To clarify fine electron behavior across the coupling interface in sub‐5 nm Fe_2_O_3_/Sm_2_O_3_@N‐CNFs, the ultraviolet photoelectron spectroscopy (UPS) tests (Figure S9) are carried out to analyze the energy bands of sub‐5 nm Fe_2_O_3_@N‐CNFs and sub‐5 nm Sm_2_O_3_@N‐CNFs. As shown in Figure 3c, the work functions (W_F_) are 3.29 eV for Fe_2_O_3_ and 4.41 eV for Sm_2_O_3_. The 1.12 eV offset (ΔW_F_) drives electron transfer from Fe_2_O_3_ (lower W_F_) to Sm_2_O_3_ (higher W_F_) upon contact until the Fermi levels equilibrate, thereby producing electron depletion in Fe_2_O_3_ and accumulation in Sm_2_O_3_. The resultant built‐in field and space‐charge regions are consistent with an increment of apparent Fe oxidation state in Fe_2_O_3_ and downward band bending in Sm_2_O_3_, indicating that interfacial coupling modulates the Fe valence via interfacial charge redistribution [38]. To further elucidate the coordination environments of Fe centers in sub‐5 nm Fe_2_O_3_/Sm_2_O_3_@N‐CNFs and sub‐5 nm Fe_2_O_3_@N‐CNFs, Fe K‐edge x‐ray absorption spectroscopy (XAS) measurements are performed. As shown in the x‐ray absorption near‐edge structure (XANES) spectra (Figure 3d), both samples exhibit pre‐edge and white‐line features highly similar to those of the Fe_2_O_3_ reference, indicating a largely preserved Fe–O coordination geometry. The absorption edges of Fe in sub‐5 nm Fe_2_O_3_/Sm_2_O_3_@N‐CNFs and sub‐5 nm Fe_2_O_3_@N‐CNFs lie between those of Fe foil and Fe_2_O_3_, where the absorption edge of Fe in sub‐5 nm Fe_2_O_3_/Sm_2_O_3_@N‐CNFs performs a marginal shift to the higher energy region relative to that in sub‐5 nm Fe_2_O_3_@N‐CNFs. As further illustrated by the corresponding valence analysis (Figure 3e), the valence state of the Fe element in sub‐5 nm Fe_2_O_3_/Sm_2_O_3_@N‐CNFs is closer to Fe(III) than that in sub‐5 nm Fe_2_O_3_@N‐CNFs. This result implies a slightly more electron‐deficient Fe environment caused by the formation of heterogeneous structure, which is consistent with XPS and UPS results. In the Fourier transform (FT) *k* ^2^‐weighted EXAFS spectra and wavelet‐transform (WT) EXAFS of the Fe element in all the samples (Figure 3f–i and Figure S10a), sub‐5 nm Fe_2_O_3_/Sm_2_O_3_@N‐CNFs exhibits a distinct coordination peak attributed to Fe–O at 1.97 Å without the existence of the Fe–Fe bond. Additionally, compared to the pure sub‐5 nm Fe_2_O_3_@N‐CNFs (2.0 Å), the shorter Fe–O bond in sub‐5 nm Fe_2_O_3_/Sm_2_O_3_@N‐CNFs suggests a change in crystal structure because of direct interface interactions between Fe_2_O_3_ and Sm_2_O_3_, thereby enhancing the covalency for promoting electron delocalization [39]. As shown in Table S1, the FT‐EXAFS spectra are fitted by using backscattering paths of Fe–O. The obtained results exhibit that the coordination number of the Fe atom in sub‐5 nm Fe_2_O_3_/Sm_2_O_3_@N‐CNFs is approximately 1.2, whereas that in sub‐5 nm Fe_2_O_3_@N‐CNFs is about 1.8. Meanwhile, the high fitting accuracy in *K*‐space of the Fe element (Figure S10b–e) further supports the precision and reliability of the coordination environment and local structural features revealed in *R*‐space (Figure S10f–i). The above results indicate that electron transfer occurs from Fe_2_O_3_ to Sm_2_O_3_ in sub‐5 nm Fe_2_O_3_/Sm_2_O_3_@N‐CNFs, demonstrating the formation of a heterogeneous interface and the electronic perturbation of Fe centers by Sm_2_O_3_, which enables the catalyst to possess high catalytic activity.

### 2.3 ORR Electrocatalytic Performance

**FIGURE 4:**
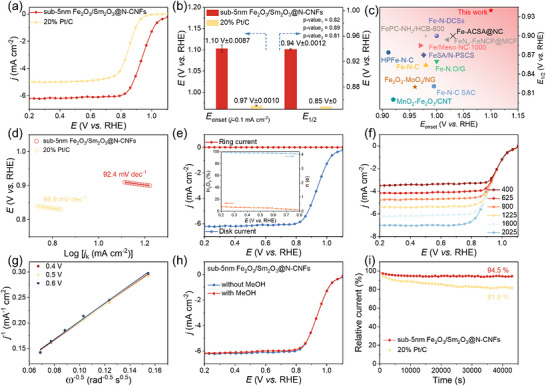
Electrocatalytic ORR performance evaluation of different samples in 0.1 M KOH medium: (a) LSV curves, (b) *E*_onset_ at 0.1 mA cm^−2^ and *E*_1/2_ of as‐prepared catalysts (error bars represent the standard deviation (SD) from three independent measurements (*n* = 3), data are presented as mean ± SD, statistical significance is evaluated using one‐way ANOVA, and at the 0.05 level, the data is significantly drawn from a normally distributed population), (c) comparison of *E*_onset_ and *E*_1/2_ with reported Fe‐based electrocatalysts, (d) Tafel slope plots, (e) RRDE curves of sub‐5 nm Fe_2_O_3_/Sm_2_O_3_@N‐CNFs (inset: H_2_O_2_ yield and ORR electron transfer number), (f) LSV curves of sub‐5 nm Fe_2_O_3_/Sm_2_O_3_@N‐CNFs at different rotating rates, (g) K–L plots of sub‐5 nm Fe_2_O_3_/Sm_2_O_3_@N‐CNFs in 0.4, 0.5, and 0.6 V, (h) methanol tolerance examinations of sub‐5 nm Fe_2_O_3_/Sm_2_O_3_@N‐CNFs, and (i) *i–t* curves of sub‐5 nm Fe_2_O_3_/Sm_2_O_3_@N‐CNFs and Pt/C catalyst.

The alkaline ORR activity of the as‐prepared sub‐5 nm Fe_2_O_3_/Sm_2_O_3_@N‐CNFs and other compared catalysts is evaluated by using a rotating ring‐disk electrode (RRDE) with a standard three‐electrode system in an O_2_‐saturated 0.1 M KOH aqueous solution. To investigate the effect of metal site size on catalytic activity, the ORR performance of the catalysts synthesized with ligands is compared to that of these samples without the addition of ligands. As shown in Figure 4a and Figures S11a,b and S12a,b, the sub‐5 nm Fe_2_O_3_@N‐CNFs, sub‐5 nm Sm_2_O_3_@N‐CNFs, and sub‐5 nm Fe_2_O_3_/Sm_2_O_3_@N‐CNFs all exhibit better ORR performance in *E*_onset_ and *E*_1/2_ than their counterpart catalysts (Fe_2_O_3_@N‐CNFs [*E*_onset_ = 0.91 V and *E*_1/2_ = 0.74 V], Sm_2_O_3_@N‐CNFs [*E*_onset_ = 0.85 V and *E*_1/2_ = 0.67 V], and Fe_2_O_3_/Sm_2_O_3_@N‐CNFs [*E*_onset_ = 1.0 V and *E*_1/2_ = 0.82 V]), respectively. This result emphasizes the crucial role of the size effect induced by ligand engineering in promoting the exposure of active sites and regulating electronic eigenstates to enhance the ORR catalytic activity. The collected cyclic voltammetry (CV) curves in both N_2_ and O_2_‐saturated 0.1 M KOH electrolyte (Figure S12c) display that the cathodic reduction peak of sub‐5 nm Fe_2_O_3_/Sm_2_O_3_@N‐CNFs appears at 0.90 V_RHE_, which is more positive than that of commercial 20% Pt/C (0.80 V_RHE_), revealing the better ORR activity of sub‐5 nm Fe_2_O_3_/Sm_2_O_3_@N‐CNFs compared with Pt/C reference. The linear sweep voltammetry (LSV) polarization curves are used to evaluate the ORR performance in detail. Figure 4b and Figure S12b show that the sub‐5 nm Fe_2_O_3_/Sm_2_O_3_@N‐CNFs exhibit a higher onset potential (*E*_onset_) of 1.1 V and half‐wave potential (*E*_1/2_) of 0.94 V than those of sub‐5 nm Fe_2_O_3_@N‐CNFs (*E*_onset_ = 1.07 V, *E*_1/2_ = 0.8 V), sub‐5 nm Sm_2_O_3_@N‐CNFs (*E*_onset_ = 0.99 V, *E*_1/2_ = 0.73 V), and benchmark 20% Pt/C (*E*_onset_ = 0.94 V, *E*_1/2_ = 0.85 V), and a vast majority of recently reported Fe‐based ORR catalysts (Figure 4c and Table S2), demonstrating the synergistic effect between Fe_2_O_3_ and Sm_2_O_3_ in catalytic ORR. Cyclic voltammetry (CV) tests within the non‐Faradaic potential range (1.01–1.11 V_RHE_) at various scanning rates (20–100 mV s^−1^, Figure S13) are performed to examine the electrochemical double‐layer capacitance (*C*_dl_) value. As exhibited in Figure S14a, the *C*_dl_ value of sub‐5 nm Fe_2_O_3_/Sm_2_O_3_@N‐CNFs is determined to be 12.0 mF cm^−2^, which is larger than that of sub‐5 nm Fe_2_O_3_@N‐CNFs (9.8 mF cm^−2^), sub‐5 nm Sm_2_O_3_@N‐CNFs (6.5 mF cm^−2^), Fe_2_O_3_/Sm_2_O_3_@N‐CNFs (10.0 mF cm^−2^), Fe_2_O_3_@N‐CNFs (7.3 mF cm^−2^) and Sm_2_O_3_@N‐CNFs (1.9 mF cm^−2^). This result indicates that sub‐5 nm Fe_2_O_3_/Sm_2_O_3_@N‐CNFs has more active sites due to the linear relationship between electrochemical active surface area (ECSA) and *C*_dl_ value, thereby revealing the promoting effect of the heterogeneous interface structure and size effect induced by ligand engineering on the exposure of more active sites. Interestingly, the electrochemical impedance spectroscopy (EIS) tests (Figure S14b and Table S3) and their corresponding fitting results indicate that the standard sub‐5 nm Fe_2_O_3_/Sm_2_O_3_@N‐CNFs exhibit a lower charge transfer resistance (*R*_ct_, 64.08 Ω) with a smaller semicircle than those of other reference samples. This result implies that interface coupling between ultrasmall heterogeneous phases can accelerate charge transfer and enhance ORR kinetics of the catalyst. The detailed reaction kinetics and mechanisms are explored by the Tafel slope (Figure 4d and Figure S15). Notably, the sub‐5 nm Fe_2_O_3_/Sm_2_O_3_@N‐CNFs perform a smaller Tafel slope (92.4 mV dec^−1^) with a transfer coefficient (α) of 0.64 than that of the sub‐5 nm Fe_2_O_3_@N‐CNFs (125.0 mV dec^−1^, *α* = 0.47), sub‐5 nm Sm_2_O_3_@N‐CNFs (156.0 mV dec^−1^, *α* = 0.38), Fe_2_O_3_/Sm_2_O_3_@N‐CNFs (95.0 mV dec^−1^, *α* = 0.62), Fe_2_O_3_@N‐CNFs (141.0 mV dec^−1^, *α* = 0.42), Sm_2_O_3_@N‐CNFs (170.0 mV dec^−1^, *α* = 0.35), N‐CNFs (196.0 mV dec^−1^, *α* = 0.30), and the commercial 20% Pt/C (98.9 mV dec^−1^, *α* = 0.60), manifesting the faster reaction kinetics of sub‐5 nm Fe_2_O_3_/Sm_2_O_3_@N‐CNFs for ORR [40]. Moreover, as shown in Figure 4e, RRDE‐measured results indicate that the peroxide (H_2_O_2_) yield on sub‐5 nm Fe_2_O_3_/Sm_2_O_3_@N‐CNFs is below 10% within the potential range from 0.2 to 0.8 V_RHE_, suggesting a high selectivity for the four‐electron pathway during ORR. Figure 4f shows that the LSV curves under different sweep rates (400 to 2025 rpm) exhibit a good linear parallel relationship. The current density linearly increases with rotation speed due to the thinning of the mass transfer diffusion layer, confirming the single reaction pathway [41]. The Koutecky–Levich (K–L) plots (Figure 4g) reveal a nice linear fitting in the representative potential range of 0.4–0.6 V, again confirming the four‐electron transfer catalytic mechanism (*n* ≈ 4) and first‐order reaction kinetics against the concentration of dissolved oxygen during the ORR process. More importantly, the methanol crossover tolerance and long‐term stability of ORR electrocatalysts are crucial for evaluating the application potential of materials in practical devices. As shown in Figure 4h and Figure S16a, compared with the sharp decline in the catalytic activity of the Pt/C benchmark, the LSV curve of sub‐5 nm Fe_2_O_3_/Sm_2_O_3_@N‐CNFs can maintain a good overlap with the initial state after injecting 1.0 M methanol into the electrolyte, demonstrating good methanol‐poisoning resistance performance and giant application potential in fuel cells. Furthermore, the durability of the catalyst is tested by the chronoamperometry method (*i*‐*t*) at a constant voltage of 0.7 V_RHE_. As shown in Figure 4i and Figure S16b, it is quite evident that after 42 000 s of continuous electrocatalysis, the current density of sub‐5 nm Fe_2_O_3_/Sm_2_O_3_@N‐CNFs only decreases by 5.5% of its initial value, surpassing that of commercial Pt/C and other reference catalysts, which indicates the marked electrochemical stability of sub‐5 nm Fe_2_O_3_/Sm_2_O_3_@N‐CNFs. Excitingly, the XRD pattern (Figure S17a), SEM image (Figure S17b), and TEM image (Figure S17c) of the post‐tested sub‐5 nm Fe_2_O_3_/Sm_2_O_3_@N‐CNFs indicate the excellent chemical stability of active sites and extraordinary mechanical robustness of sub‐5 nm Fe_2_O_3_/Sm_2_O_3_@N‐CNFs. Meanwhile, the inductively coupled plasma optical emission spectrometry (ICP‐OES) analysis (Table S4) confirms that the Fe and Sm contents in the sub‐5 nm Fe_2_O_3_/Sm_2_O_3_@N‐CNFs catalyst are 9.34 wt% and 10.52 wt%, respectively. After durability testing, the ICP‐OES result shows that the Fe and Sm contents in the post‐tested sub‐5 nm Fe_2_O_3_/Sm_2_O_3_@N‐CNFs are 9.20 wt% and 9.98 wt%, respectively, indicating minimal leaching and excellent compositional stability under operating conditions. Moreover, XPS analysis of post‐tested sub‐5 nm Fe_2_O_3_/Sm_2_O_3_@N‐CNFs (Figure S17d,e) reveals that the Fe 2p and Sm 3d spectra remain characteristic of Fe^3+^ and Sm^3+^ species, respectively, confirming the chemical robustness of the active centers.

### 2.4 Theoretical Simulations and In Situ Spectroscopy Insights Into the ORR Mechanism

**FIGURE 5:**
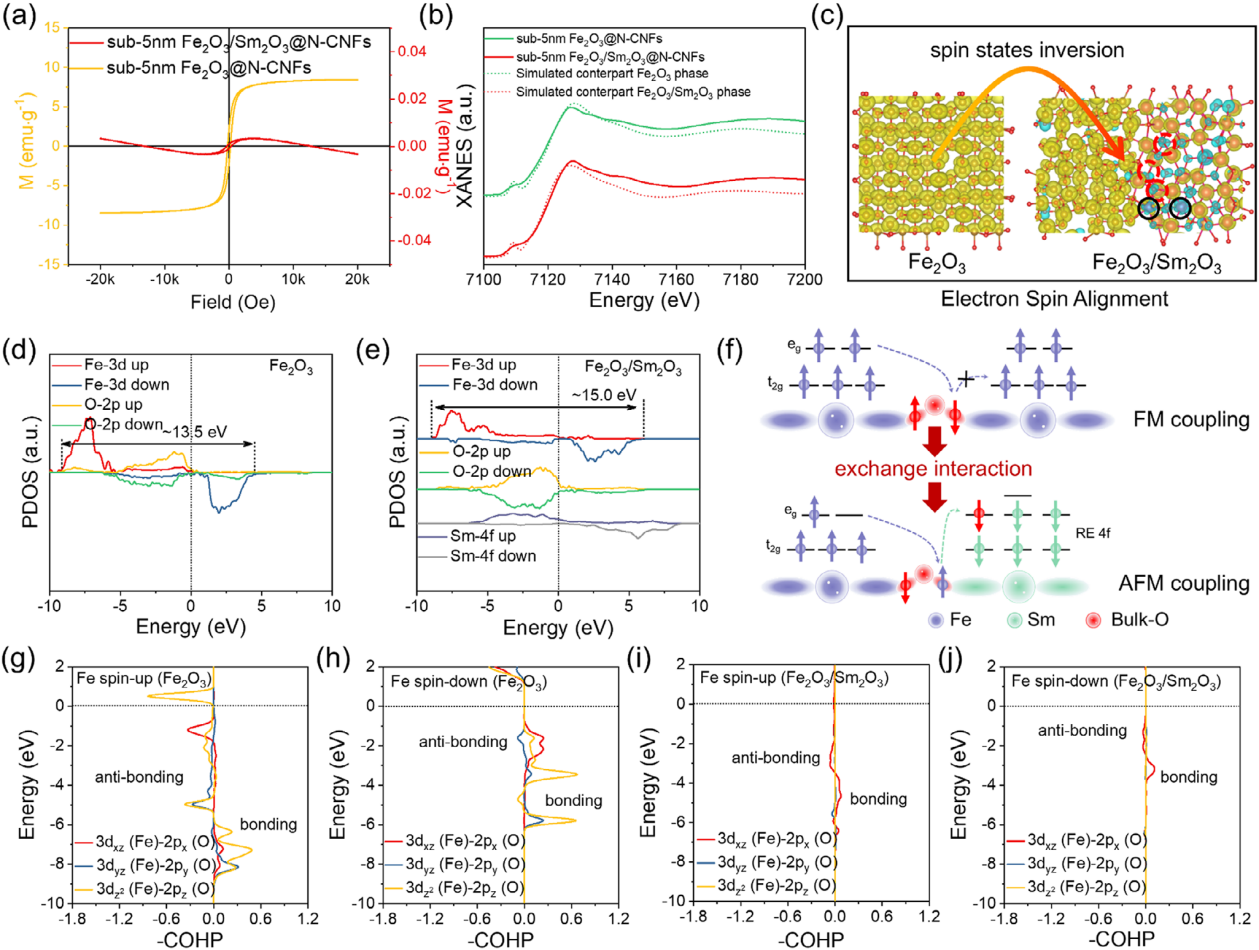
Magnetic and theoretical analysis of sub‐5 nm Fe_2_O_3_@N‐CNFs and sub‐5 nm Fe_2_O_3_/Sm_2_O_3_@N‐CNFs: (a) magnetic hysteresis loops of catalysts at room temperature, (b) the x‐ray absorption near‐edge structure of actual and simulated Fe_2_O_3_@N‐CNFs and Fe_2_O_3_/Sm_2_O_3_@N‐CNFs, (c) spin‐polarized density of states (DOS) analysis, (d), (e) projected density of states (PDOS), (f) schematic diagrams of FM and AFM interactions, respectively, and (g–j) crystal orbital Hamilton population (COHP) analysis.

To elucidate the role of intrinsic magnetic transitions in enhancing catalytic activity, vibrating sample magnetometry (VSM) is employed to characterize the magnetization of sub‐5 nm Fe_2_O_3_/Sm_2_O_3_@N‐CNFs and pristine sub‐5 nm Fe_2_O_3_@N‐CNFs. As shown in Figure 5a, sub‐5 nm Fe_2_O_3_@N‐CNFs exhibits a typical ferromagnetic signal with a saturation magnetization of ∼8.5 emu g^−1^, accompanied by pronounced coercive magnetic field (*H*_c_) and residual magnetization (*M*_r_), therefore reflecting its long‐range ferromagnetic order. In contrast, Fe_2_O_3_/Sm_2_O_3_@N‐CNFs displays a dramatically reduced magnetization (< 0.01 emu g^−1^) with weak hysteresis near *H* = 0, which can be attributed to the mutual cancellation of magnetic moments between the heterogeneous components driven by interfacial exchange coupling at the Fe_2_O_3_/Sm_2_O_3_ interface. Furthermore, under high magnetic fields, a negative slope in the magnetic hysteresis loops reflects dominant diamagnetic behavior stemming from the Lenz‐type response of electron orbitals [42, 43]. This mixed magnetic behavior may result from the antiparallel alignment of electrons arising from a strong super‐exchange‐induced antiferromagnetic coupling at the interface, thereby suppressing ferromagnetism and amplifying the diamagnetic contribution at high fields. Furthermore, the temperature‐dependent magnetization curves (Figure S18a) and corresponding fitting results (Figure S18c) of sub‐5 nm Fe_2_O_3_/Sm_2_O_3_@N‐CNFs show a lower net magnetization (*C* = +0.00018 emu K g^−1^ Oe^−1^, Curie constant; the sign indicates that they are in the same or opposite direction as the external magnetic field) and a stronger exchange interaction (*θ =* −5974.44 K, Weiss temperature, proportional to the exchange coupling constant) than those of sub‐5 nm Fe_2_O_3_@N‐CNFs (*C* = −0.00811 emu K g^−1^ Oe^−1^, *θ =* 4902.50 K) (Figure S18b,d), which may indicate the presence of interfacial super‐exchange interactions. Notably, such interfacial spin interactions are expected to perturb the spin order of Fe sites, thereby modulating the adsorption behavior of ORR intermediates and providing a plausible origin for the enhanced catalytic activity. Therefore, the density functional theory (DFT) is used to further validate the interfacial spin modulation mechanism in ORR, where Fe_2_O_3_/Sm_2_O_3_, Fe_2_O_3_, and Sm_2_O_3_ models are built (Figure S19). Among these, as shown in Figure 5b, the simulated Fe *K*‐edge XANES spectra based on the structures of the optimized Fe_2_O_3_ model and Fe_2_O_3_/Sm_2_O_3_ model match well the tested spectra of sub‐5 nm Fe_2_O_3_@N‐CNFs and sub‐5 nm Fe_2_O_3_/Sm_2_O_3_@N‐CNFs, thereby verifying the rationality of theoretical models.

The spin‐polarized density of states (DOS) analysis (Figure 5c) reveals distinct electronic spin configurations in heterogeneous Fe_2_O_3_/Sm_2_O_3_ and pristine Fe_2_O_3_. The results show that compared with Fe and O atoms with completely co‐directional spin alignment in Fe_2_O_3_, part of the Sm atoms (marked by the black circle) display antiparallel spin polarization with Fe atoms at the Fe_2_O_3_/Sm_2_O_3_ interface. Notably, a portion of bridging oxygen atoms (marked by the red circle) also undergoes spin reorientation, which is attributed to super‐exchange‐induced spin redistribution. As shown in Figure S20, Bader charge analysis and differential charge density calculations confirm electron transfer at the Fe_2_O_3_/Sm_2_O_3_ interface, where approximately 3.5 electrons are transferred from Fe_2_O_3_ to Sm_2_O_3_, verifying the aforementioned electronic structure information obtained from the physical characterizations. This electronic redistribution results in charge accumulation at Sm sites and depletion at Fe sites, thereby reinforcing Fe–O–Sm orbital hybridization and enhancing the super‐exchange interaction, which stabilizes antiferromagnetic order. The PDOS analyses (Figure 5d) further reveal that pristine Fe_2_O_3_ exhibits a narrow spin‐polarized‐dominated Fe 3d band (∼13.5 eV), indicating the low scalability of electrons in real space and concentrated electron states in energy, which results in a low degree of orbital hybridization between atoms. Upon heterojunction formation, the Fe 3d bandwidth (Figure 5e) broadens to ∼15 eV, accompanied by a visibly decreased polarization intensity, reflecting the suppressed spin polarization and the increased electronic delocalization. This subtle variation is attributed to the strong band dispersion of the O 2p orbital, which can well overlap with Fe 3d and Sm 4f orbitals, thus effectively bridging the localized 3d and 4f orbitals [20, 22]. The resulting [Fe–O–Sm] coupling units enhance interfacial orbital hybridization and covalency, broadening the Fe 3d band to improve the delocalization properties of electrons, thereby providing an accessible channel for facilitating super‐exchange‐type electron transfer across the interface [44]. The mechanism of this super‐exchange effect is shown in Figure 5f. Specifically, in ferromagnetic Fe_2_O_3_, Fe^3+^ ions are bridged by O atoms to form Fe–O–Fe linkages. Based on Hund's rule, the half‐filled Fe 3d orbitals with exceptional stability prevent direct electron hopping, thereby resisting electron pairing to minimize coulomb repulsion [32]. In contrast, in the non‐periodic Fe–O–Sm configuration, spin‐asymmetric coupling permits electrons from O 2p to empty Sm 4f orbitals, while Fe 3d valence electrons refill the resulting O 2p holes [33]. As shown in Figure S21a, this super‐exchange induced by spin selectivity strengthens the interaction between Fe and O atoms in the bulk, which results in shortening of the Fe–O bond, especially at the interface [45, 46]. Meanwhile, the shortening of the distance between the Fe atom and the coordination O atom in the plane leads to an increase in the coordination saturation of the metal center, thereby weakening its Lewis acidity toward the axial OH* and reducing the axial OH adsorption strength [39, 47]. Thus, as exhibited in Figure S21b, the bond length of the Fe–O bond between the active center and the adsorbed OH* increases from 1.8 Å in the pristine Fe_2_O_3_ to 2.0 Å in the Fe_2_O_3_/Sm_2_O_3_, indicating that the super‐exchange interaction in the [Fe–O–Sm] unit can effectively promote the desorption of OH*. The PDOS of OH* adsorbed by active sites (Figure S22) shows that the introduction of Sm_2_O_3_ significantly reduces the orbital overlap between the Fe 3d orbitals and the O 2p orbitals of OH*, thereby weakening OH* adsorption. Crystal orbital Hamilton population (COHP) analysis (Figure 5 g,h) exhibits the adsorption state of OH* on the surface, where COHP values for Fe 3d orbitals (3d_yz_, 3d_z_2, and 3d_xz_) in pristine Fe_2_O_3_ are more negative, confirming robust Fe–OH bonding at its surface. Meanwhile, the –COHP values for spin‐down states are higher than those for spin‐up, especially for 3d_z_2, reflecting strong *σ*‐type bonding driven by magnetic character. In Fe_2_O_3_/Sm_2_O_3_, the ‐COHP of [Fe–OH] (Figure 5i,j) exhibits a marked drop with 3d_z_2 bonding contributions approaching zero, and the antibonding population of [Fe–OH] in Fe_2_O_3_/Sm_2_O_3_ moves closer to *E*_F_. These results indicate that the regulation of magnetic behavior induced by the super‐exchange interaction at the heterointerface can markedly weaken σ‐bonding between sites and intermediates and enhance electronic delocalization, thus diminishing Fe–OH interactions to facilitate the transformation of OH*. Additionally, to examine the interfacial interaction between different rare‐earth oxides and Fe_2_O_3_, we further selected the neighboring rare‐earth elements Pm and Eu, which possess one fewer and one more valence electron than Sm, respectively. The results show that in the optimized Fe_2_O_3_/Eu_2_O_3_ model (Figure S23a), both the Eu (Figure S23b) and Fe (Figure S23c) sites exhibit strong OH* bonding, similar to the Pm and Fe sites in Fe_2_O_3_/Pm_2_O_3_ (Figure S23d–f). Notably, this bonding is significantly stronger than that of the Sm sites and OH* (Figure S23g). These results indicate that rare‐earth elements with non‐matching valence states lead to excessively strong adsorption, whereas valence‐electron matching enables more balanced OH* adsorption, supporting the proposed magnetic modulation model.

**FIGURE 6:**
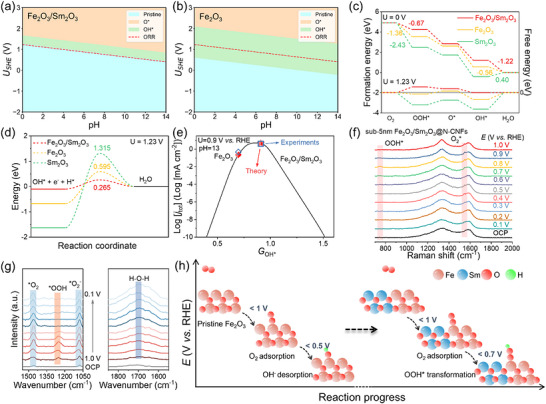
Surface Pourbaix diagrams of (a) Fe_2_O_3_/Sm_2_O_3_, (b) Fe_2_O_3_. Reaction pathway analysis of sub‐5 nm Fe_2_O_3_/Sm_2_O_3_@N‐CNFs and sub‐5 nm Fe_2_O_3_@N‐CNFs: (c) ORR free energy, (d) analysis of the OH* desorption transition state, (e) ORR volcano activity model predicting the exchange current density (*j*_0_) as a function of Δ*G*_OH*_ (pH = 13, U = 0.9 V vs. RHE), (f) in situ enhanced Raman spectroscopy of sub‐5 nm Fe_2_O_3_/Sm_2_O_3_@N‐CNFs, (g) in situ ATR‐SEIRAS of sub‐5 nm Fe_2_O_3_/Sm_2_O_3_@N‐CNFs, and (h) schematic comparison of the ORR reaction progress.

To further clarify the catalytic mechanism at the surface, a surface Pourbaix diagram is used to analyze the functional relationship between the applied potential versus SHE (*U*_SHE_) and pH, where various hydroxyl coverages, including OH* and O*, have been considered using DFT calculations. As shown in Figure 6a,b, the thermodynamic equilibrium line of ORR is located at its pristine surface state for Fe_2_O_3_/Sm_2_O_3_ heterostructure, whereas the functional line in Fe_2_O_3_ intersects the OH*‐passivated domain within the operational ORR window. This observation confirms that along the specific voltage‐pH trajectory required for ORR, interfacial electronic spin modulation in Fe_2_O_3_/Sm_2_O_3_ effectively suppresses surface OH* coverage, thereby facilitating OH* desorption to enhance the overall catalytic efficiency. Consequently, this super exchange interaction in the [Fe–O–Sm] unit synchronizes the catalytic thermodynamic stability with the ORR working window, fundamentally avoiding the OH*‐poisoning effect and ensuring superior intrinsic activity. Therefore, as shown in Figure 6c and Figures S24 and S25, the potential‐determining step (PDS) transforms from OH* decoupling on Fe_2_O_3_, Sm_2_O_3_, and Fe_2_O_3_/Sm_2_O_3_ with Sm as the sites to the formation of OOH* on Fe_2_O_3_/Sm_2_O_3_ with Fe as the sites, generating a better overall thermodynamic spontaneous trend with the lowest PDS free energy (−0.67 eV). Additionally, the transition state analysis (Figure 6d) demonstrates that compared to the pristine Fe_2_O_3_ (1.155 eV), the critical step of OH* reduction on Fe_2_O_3_/Sm_2_O_3_ exhibits a lower activation energy of about 0.266 eV, which suggests that the interaction at the Fe–O–Sm interface can optimize the binding strength of OH* species at the active sites to boost kinetics. To further assess intrinsic ORR activity, we map the OH* free energies of the various catalysts onto the alkaline ORR kinetic volcano plot, in which the model explicitly considers reaction kinetics under electrochemical conditions. As shown in Figure 6e, Fe_2_O_3_/Sm_2_O_3_ is located closer to the apex of the state‐of‐the‐art ORR microkinetic volcano (microkinetic modeling considering the operating coverage, exhaustive elementary steps, potential, pH effects under the RHE scale via electric field effect simulations, potential of zero charge, etc.) as a function of OH* binding energy, indicating that its OH* binding energetics are near‐optimal and its catalytic performance approaches the theoretical maximum. Consequently, Fe_2_O_3_/Sm_2_O_3_ exhibits a higher exchange current density (*j*_0_) than Fe_2_O_3_. Notably, the predicted current density by the microkinetic modeling is in excellent agreement with our experimental results in Figure 4a (for Fe_2_O_3_/Sm_2_O_3_: simulation, 0.654 log(mA cm^−2^) at 0.9 V versus RHE; experiment, 0.626 log(mA cm^−2^) at 0.9 V versus RHE; as shown in Figure 6e), which suggests the high accuracy of our simulation methods and the rationality of the catalyst surface structures considered in this study. All the above results manifest that the transformation of crystal structure and aligned electronic arrangement promotes interfacial super‐exchange interaction and suppresses spin polarization, thereby boosting the reduction of OH* to improve ORR. Subsequently, in situ enhanced Raman spectroscopy is employed to monitor the adsorption behavior of ORR intermediates to validate the proposed ORR mechanism in O_2_‐saturated 0.1 M KOH. For the sub‐5 nm Fe_2_O_3_/Sm_2_O_3_@N‐CNFs (Figure 6f), two distinct signal responses are observed at approximately 750 and 1530 cm^−1^, corresponding to the O–O stretching vibration in OOH and the O–O vibration of surface‐adsorbed O_2_*, respectively [48, 49]. Notably, as the potential decreases to 0.7 V versus RHE, the signal of OOH* nearly vanishes. In contrast, the signal of O_2_* persists until the potential is reduced to 0.2 V versus RHE. This result suggests that in the kinetically controlled region, the majority of O_2_ adsorbed on Fe sites is converted to OOH*. In the diffusion‐controlled region, the increased current density at lower potentials facilitates the further transformation of O_2_ toward fully reduced products. This behavior is consistent with a reduced potential‐dependent barrier on the Fe_2_O_3_/Sm_2_O_3_ composite, enabling the PDS to occur at more positive potentials during the ORR. In contrast, for pristine sub‐5 nm Fe_2_O_3_@N‐CNFs (Figure S26a) and sub‐5 nm Sm_2_O_3_@N‐CNFs (Figure S26b), upon reducing to lower voltage (0.7 V vs. RHE, and 0.6 V vs. RHE, respectively), a prominent OH* signal appears at ∼1250 cm^−1^, which is attributed to the Fe–OH bending mode [50]. Moreover, this characteristic peak persists down to 0.5 V versus RHE before disappearing, indicating the stepwise reduction of O_2_ to OH* followed by complete OH* desorption. In addition, Figure S26c,e reveals that the sub‐5 nm Fe_2_O_3_/Sm_2_O_3_@N‐CNFs exhibits minimal variation in the *I*_D_/*I*_G_ ratio throughout the potential sweep, underscoring their superior structural stability compared to sub‐5 nm Fe_2_O_3_@N‐CNFs with more changes [51]. Furthermore, the in situ attenuated total reflectance surface‐enhanced infrared absorption spectroscopy (ATR‐SEIRAS) is used to identify the key reactive intermediates. As shown in Figure 6g and Figure S27c, the sub‐5 nm Fe_2_O_3_/Sm_2_O_3_@N‐CNFs catalyst exhibits the absorbance peaks at ∼1450 and ∼1100 cm^−1^, which can be attributed to the O–O stretching mode of adsorbed O_2_ and O_2_^−^ species [52]. These peaks gradually emerge as the potential decreases to 0.7 V_RHE_ and subsequently remain nearly constant, indicating that a more negative potential can effectively promote the surface coverage of O_2_*, thereby facilitating the initiation of the reaction. Moreover, when the potential further decreases below 0.6 V_RHE_, corresponding to the diffusion‐limited region, a dynamic equilibrium between the adsorption and transformation of O_2_ is established. Noteworthily, the peak at ∼1250 cm^−1^, assigned to the bending mode vibrations of adsorbed OOH* intermediates, continuously increases with a decrease in potential until 0.7 V_RHE_, manifesting the progressive formation of OOH* species and the accelerated conversion of O_2_* to OOH* under a mixed kinetic‐diffusion region [52]. For comparison, both the sub‐5 nm Fe_2_O_3_@N‐CNFs (Figure S27a,d) and sub‐5 nm Sm_2_O_3_@N‐CNFs (Figure S27b,e) display two dominant absorption peaks at ∼1700 and ∼1450 cm^−1^, which are attributed to bending vibration of H–O–H and O_2_*, respectively [53]. As the potential decreases, the O_2_* signal gradually increases and reaches a steady state, while the H–O–H band becomes progressively stronger, signifying more pronounced hydroxyl adsorption compared with sub‐5 nm Fe_2_O_3_/Sm_2_O_3_@N‐CNFs. These in situ experimental findings align with the theoretical simulations above, demonstrating that the Fe_2_O_3_/Sm_2_O_3_ heterojunction can transform the PDS from difficult OH* desorption on Fe_2_O_3_ and Sm_2_O_3_ to effective OOH* formation at the heterointerface, thereby accelerating overall ORR kinetics (Figure 6h). Consequently, sub‐5 nm Fe_2_O_3_/Sm_2_O_3_@N‐CNFs exhibits superior electrocatalytic performance, offering a robust platform for efficient ORR in alkaline media.

### 2.5 Liquid and All‐Solid‐State Flexible ZAB

**FIGURE 7:**
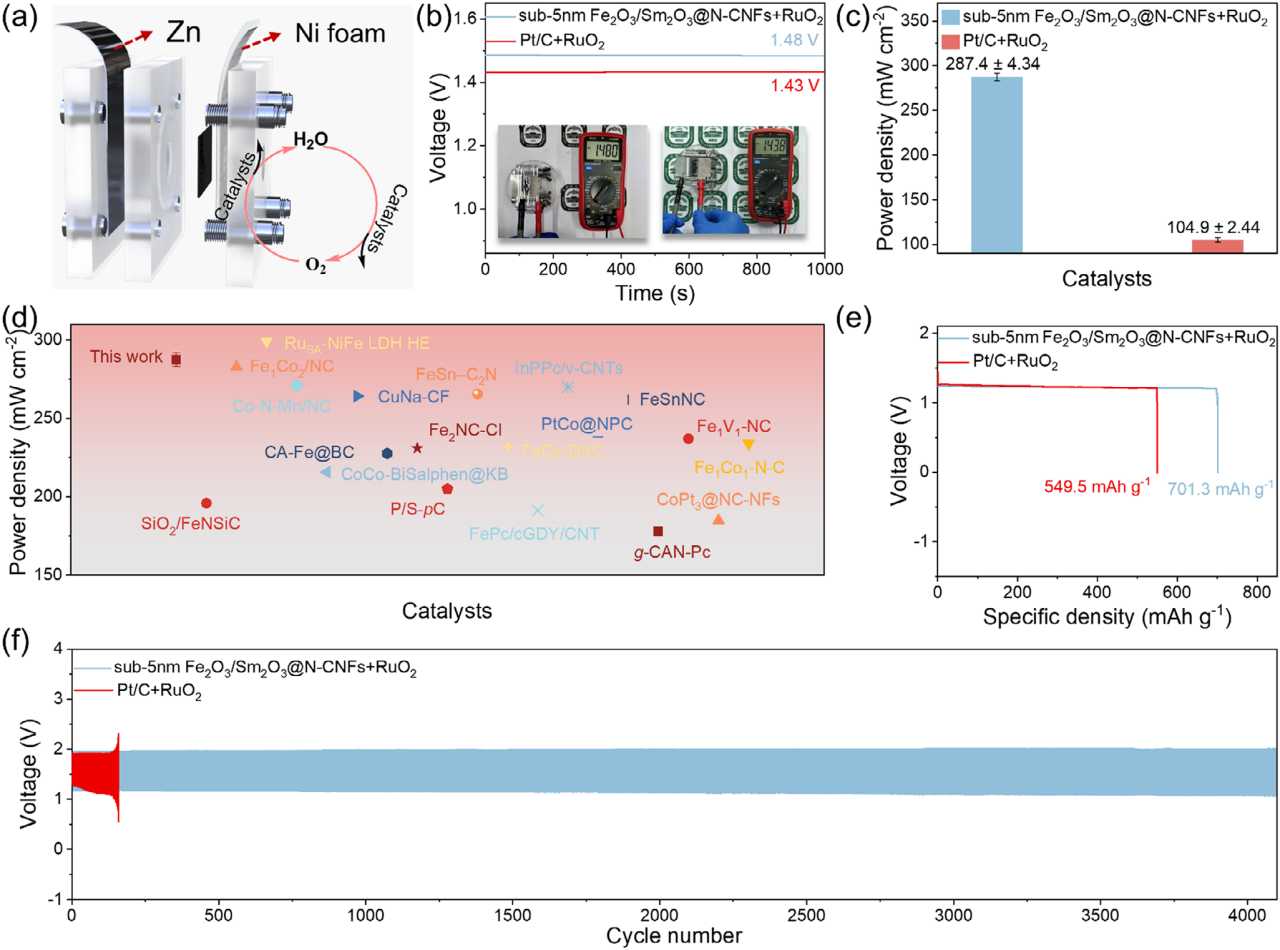
The performance comparison of liquid ZABs assembled with sub‐5 nm Fe_2_O_3_/Sm_2_O_3_@N‐CNFs + RuO_2_ and Pt/C + RuO_2_: (a) schematic illustration of the as‐prepared liquid ZABs, (b) the open‐circuit voltage (OCVs) plots (inset: the OCV recorded by a multimeter), (c) the power density, (d) comparison of power density with the recently reported ZABs, (e) specific capacities, and (f) the galvanostatic discharge‐charge cycling curves.

To further demonstrate the practical applicability of the sub‐5 nm Fe_2_O_3_/Sm_2_O_3_@N‐CNFs catalyst, a rechargeable liquid ZAB is fabricated by using a zinc plate as the anode and Ni foam as the cathode loaded with a mixture of sub‐5 nm Fe_2_O_3_/Sm_2_O_3_@N‐CNFs and RuO_2_ (Figure 7a). For a benchmark, a commercial Pt/C + RuO_2_‐based ZAB is constructed under identical conditions. As shown in Figure 7b, the sub‐5 nm Fe_2_O_3_/Sm_2_O_3_@N‐CNFs‐based ZAB delivers a higher open‐circuit voltage (OCV) of 1.48 V than that of the Pt/C‐based counterpart (1.43 V), suggesting the potential to power real devices. As expected, two series‐connected sub‐5 nm Fe_2_O_3_/Sm_2_O_3_@N‐CNFs‐based ZABs successfully light a light‐emitting diode (LED, ∼1.5 V) panel with “NNU” words (Figure S28a). Key performance metrics including power density and specific capacity are used to assess the practical operational efficiency of the equipment. Thus, as exhibited in Figure 7c–e and Figure S28b,c, the sub‐5 nm Fe_2_O_3_/Sm_2_O_3_@N‐CNFs‐based ZAB achieves a peak power density of 287.4 mW cm^−2^ and a specific capacity of 701.3 mAh g^−1^, outperforming the commercial Pt/C + RuO_2_ system (104.9 mW cm^−2^ and 549.5 mAh g^−1^) and most previously reported noble‐metal‐free ZABs (Table S5). The galvanostatic discharge curves at various current densities (1, 5, and 10 mA cm^−2^) (Figure S28d) manifest that the sub‐5 nm Fe_2_O_3_/Sm_2_O_3_@N‐CNFs‐based ZAB consistently maintains higher voltage plateaus than those of the Pt/C reference. Meanwhile, upon returning to lower current densities, the discharge potential of the sub‐5 nm Fe_2_O_3_/Sm_2_O_3_@N‐CNFs‐based ZAB rapidly recovers to its corresponding initial state, reflecting excellent rate capability and electrochemical reversibility. As a critical factor for practical energy devices, stability is assessed via a continuous charge‐discharge cycling test at 5 mA cm^−2^ with 20‐min intervals. As described in Figure 7f, the sub‐5 nm Fe_2_O_3_/Sm_2_O_3_@N‐CNFs‐based ZAB exhibits excellent cycling durability with 4100 stable cycles, exceeding the 146‐cycle lifespan of the Pt/C‐based system.

**FIGURE 8:**
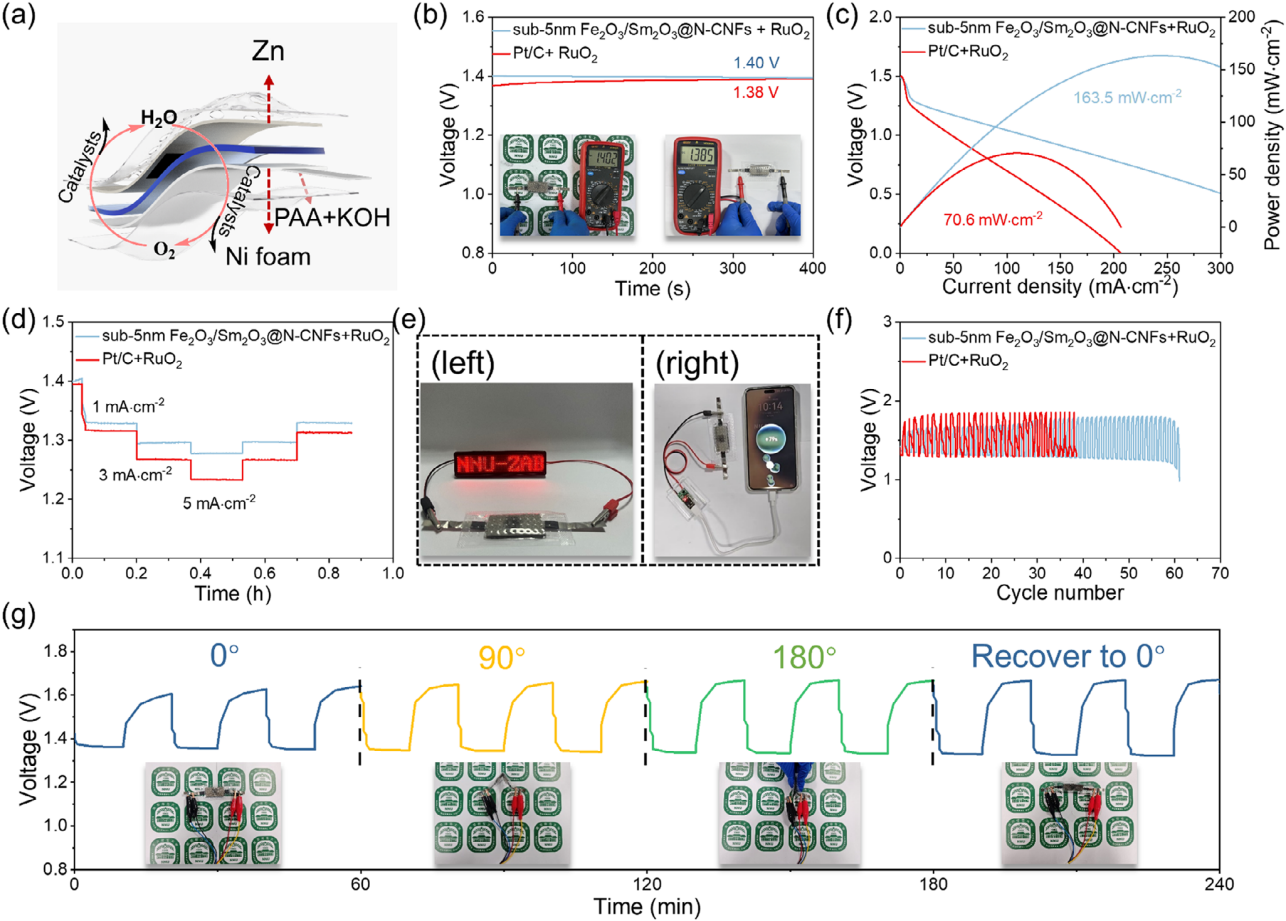
The performance comparison of all‐solid‐state flexible ZABs assembled with sub‐5 nm Fe_2_O_3_/Sm_2_O_3_@N‐CNFs + RuO_2_ and Pt/C + RuO_2_: (a) schematic illustration of the as‐prepared liquid ZABs, (b) the open‐circuit voltage (OCVs) plots (inset: the OCV recorded by a multimeter), (c) power density curves, (d) the discharge curves, (e) the photos of an LED and smartphone screen charged by a sub‐5 nm Fe_2_O_3_/Sm_2_O_3_@N‐CNFs‐based ZAB, (f) the galvanostatic discharge‐charge cycling curves, and (g) the galvanostatic discharge‐charge curves at different bending angles.

Due to the marked performance of the self‐made catalyst in the liquid ZAB, the flexible all‐solid‐state ZAB fabricated with the sub‐5 nm Fe_2_O_3_/Sm_2_O_3_@N‐CNFs and RuO_2_ is further used to investigate a broader application environment. As illustrated in Figure 8a, the all‐solid‐state ZAB is composed of nickel foam coated with catalysts as the cathode, polished zinc foil as the anode, and polyacrylic acid (PAA)/KOH gel as the solid electrolyte, respectively. As shown in Figure 8b–d, in light of the excellent performance of the synthesized catalyst in the previous liquid ZAB, the sub‐5 nm Fe_2_O_3_/Sm_2_O_3_@N‐CNFs + RuO_2_‐assembled solid‐state ZAB also exhibits a higher open‐circuit voltage (1.40 V), a higher peak power density (163.5 mW cm^−2^), and better rate performance in different current densities (1, 3 and 5 mA cm^−2^), exceeding those of commercial ZAB (1.38 V and 70.6 mW cm^−2^). Furthermore, the sub‐5 nm Fe_2_O_3_/Sm_2_O_3_@N‐CNFs‐based ZAB is connected to various devices to test the application capabilities of the battery. Impressively, the sub‐5 nm Fe_2_O_3_/Sm_2_O_3_@N‐CNFs‐based ZAB can not only smoothly power a small LED lamp (∼1.5 V) with the “NNU‐ZAB” word (left of Figure 8e) but even charge a smartphone (right of Figure 8e), demonstrating its potential for practical electronics. Additionally, the long‐term cycling stability of the ZAB is evaluated by continuous charge‐discharge testing at 1 mA cm^−2^ with 20 min per cycle. As displayed in Figure 8f, the sub‐5 nm Fe_2_O_3_/Sm_2_O_3_@N‐CNFs‐based solid‐state ZAB presents an extremely stable discharge‐charge curve and the long operating duration of 20 h (60 cycles), surpassing that of the Pt/C‐based solid‐state ZAB (39 cycles). Encouragingly, Figure 8g shows that under continuous operation, the sub‐5 nm Fe_2_O_3_/Sm_2_O_3_@N‐CNFs‐based all‐solid‐state ZAB can maintain a stable voltage plateau during folding experiments at different angles. The above results clearly highlight the practical potential of sub‐5 nm Fe_2_O_3_/Sm_2_O_3_@N‐CNFs in various flexible and wearable electronic devices.

## 3 Conclusion

In summary, we have established oxide‐heterointerface spin modulation as an effective strategy to optimize ORR intermediate binding and kinetics beyond intrinsic spin‐state constraints. Based on the above, the as‐prepared sub‐5 nm Fe_2_O_3_/Sm_2_O_3_@N‐CNFs catalyst delivers excellent ORR performance in alkaline conditions. Advanced experimental and computational analyses collectively show that the strongly coupled heterointerfaces and the work‐function mismatch drive electron transfer from Fe to Sm through bridged O atoms. This charge redistribution strengthens the interfacial Fe–O–Sm super‐exchange interaction and induces antiparallel spin alignment. As a result, the ferromagnetic spin coupling with OH* is suppressed, Fe–OH *σ*‐bonding is weakened, and the ORR barrier is lowered, resulting in accelerated ORR kinetics. Consistent with this mechanism, the sub‐5 nm Fe_2_O_3_/Sm_2_O_3_@N‐CNFs catalyst demonstrates state‐of‐the‐art catalytic activity (half‐wave potential of 0.94 V), fast reaction kinetics (Tafel slope of 92.4 mV dec^−1^), and superb stability in 0.1 M KOH. Furthermore, as the air cathode of ZAB, sub‐5 nm Fe_2_O_3_/Sm_2_O_3_@N‐CNFs‐based ZAB affords better performance than commercial ZAB in both the liquid and all‐solid‐state devices. These results establish valence‐electron‐compatibility‐enabled interfacial spin regulation as a general design principle to enhance alkaline ORR by optimizing oxygenated‐intermediate binding. Given the ubiquity of anion‐bridged exchange pathways in magnetic oxides, this concept can be extended to other electrocatalytic processes involving spin‐dependent interfacial steps.

## Author Contributions

**Jing Li**: investigation, validation, visualization, writing – original draft, writing – review and editing, formal analysis, data curation. **Ningkang Peng**: investigation, formal analysis. **Jianhua Ma**: investigation, formal analysis. **Tingyu Lu**: investigation, validation, writing – original draft, writing – review and editing, formal analysis, data curation, visualization, conceptualization. **Haibin Zhu**: investigation, formal analysis. **Guangyao Zhou**: investigation, formal analysis. **Yizhou Zhang**: investigation, formal analysis, funding acquisition, writing – review and editing. **Yizhou Zhang**: investigation, formal analysis, funding acquisition, writing – review and editing. **Yanhui Gu**: investigation, formal analysis. **Yawen Tang**: conceptualization, funding acquisition, investigation, writing – review and editing, visualization, validation, methodology, software, formal analysis, project administration, data curation, supervision, resources. **Hao Li**: conceptualization, investigation, funding acquisition, methodology, validation, visualization, writing – review and editing, software, formal analysis, project administration, data curation, supervision, resources.

## Conflicts of Interest

The authors declare no conflicts of interest.

## Supporting information




**Supporting File**: anie72581‐sup‐0001‐SuppMat.docx

## Data Availability

The data that support the findings of this study are available from the corresponding author upon reasonable request. Besides, the key experimental and computational results have also been uploaded to our Digital Catalysis Platform (*DigCat*: www.digcat.org).
