# Supplementary material for: Matching the Coupling of Valence Electrons in the Oxide Interface to Perturb the Magnetic Order Enhancing Oxygen Reduction in Zinc–Air Batteries
Source: Angew Chem Int Ed Engl. 2026 May 25;65(30):e7852726. doi: 10.1002/anie.7852726 (PMC13383175; doi:10.1002/anie.7852726)
Supplement: Supplementary file 1 — Supporting File: anie72581‐sup‐0001‐SuppMat.docx [file ANIE-65-e7852726-s001.docx]

**Matching the Coupling of Valence Electrons in the Oxide Interface to Perturb the Magnetic Order Enhancing Oxygen Reduction in Zinc-Air Batteries**

Jing Li,^[a],[d]^ Ningkang Peng,^[e]^ Jianhua Ma,^[d]^ Tingyu Lu,*^[b]^ Haibin Zhu,*^[a]^ Guangyao Zhou,*^[c]^ Yizhou Zhang,^[b]^ Yanhui Gu,^[e]^ Yawen Tang,^[d]^ and Hao Li*^[b]^

[a] J. Li, H. Zhu

School of Chemistry and Chemical Engineering,

Southeast University, Nanjing 211189, P. R. China;

E-mail address: zhuhaibin@seu.edu.cn (H. Zhu)

[b] T. Lu, Y. Zhang, H. Li

Advanced Institute for Materials Research (WPI-AIMR),

Tohoku University, Sendai 980-8577, Japan;

E-mail address: tingyu.lu.e7@tohoku.ac.jp (T. Lu), li.hao.b8@tohoku.ac.jp (H. Li)

[c] G. Zhou

College of Science,

Jinling Institute of Technology, Nanjing 211169, P. R. China;

E-mail address: zhouguangyao@jit.edu.cn (G. Zhou)

[d] J. Li, J. Ma, Y. Tang

School of Chemistry and Materials Science, Jiangsu Key Laboratory of New Power Batteries, Jiangsu Collaborative Innovation Center of Biomedical Functional Materials,

Nanjing Normal University, Nanjing 210023, P. R. China

[e] N. Peng, Y. Gu

School of Computer and Electronic Information,

Nanjing Normal University, Nanjing 210023, P. R. China

**Experimental section**

*Reagents and Chemicals*

The polyacrylonitrile (PAN, M_w_=150000), ferric chloride (FeCl_3_), samarium (III) chloride (SmCl_3_), 1,4-dicarboxybenzene (H_2_BDC), N, N-dimethylformamide (DMF), and Potassium hydroxide (KOH) are purchased from Sinopharm Chemical Reagent Co., Ltd. The Zinc Chloride (ZnCl_2_) is purchased from Shanghai Aladdin Biochemical Technology Co., Ltd., the commercial 20 wt.% Pt/C and RuO_2_ are provided by Johnson Matthey Chemicals, Ltd. All reagents and chemicals are used as received without further purification.

*Synthesis of sub-5nm Fe_2_O_3_/Sm_2_O_3_@N-CNFs and Other Compared Samples*

In a typical synthetic precursor, a mixed solution is prepared by sequentially dispersing 0.8 g PAN and 0.249 g H_2_BDC in 10 mL DMF for 12 h. Then, 0.162 g FeCl_3_ and 0.128 g SmCl_3_ are added into the white solution, accompanied by stirring for 6 h and sonicating for 1 h to form the yellow solution. The resulting yellow solution is electro-spun at ~21 kV with a 15 cm tip-to-collector distance. The collected fibrous membrane is pre-oxidized at 100 °C for 4 h, and then 200 °C for 4 h (at 2 °C min^-1^) in air. Subsequently, a high-temperature carbonization process is carried out at 800 °C for 2h under a nitrogen atmosphere, with a heating rate of 5 °C min^-1^. This process ultimately yields ultra-small Fe_2_O_3_/Sm_2_O_3_ heterojunction nanoparticles anchored on one-dimensional porous nitrogen-doped carbon nanofibers (denoted as sub-5nm Fe_2_O_3_/Sm_2_O_3_@N-CNFs). For comparison, the samples are prepared under the same synthesis conditions except for the composition of the electrospinning solution. Specifically, pyrolysis of nanofiber precursors containing only a single metal source produces sub-5nm Fe_2_O_3_@N-CNFs and sub-5nm Sm_2_O_3_@N-CNFs. N-CNFs is obtained without the addition of metal source. In addition, samples without H_2_BDC are prepared under the same conditions and named Fe_2_O_3_/Sm_2_O_3_@N-CNFs, Fe_2_O_3_@N-CNFs, and Sm_2_O_3_@N-CNFs, respectively.

*Materials Characterization*

Transmission electron microscopy (TEM) and high-resolution transmission electron

microscopy (HRTEM) are performed on a Jeol JEM-2010F (200 kV). Scanning electron microscopy (SEM) images are acquired on a Hitachi S5500. High-angle annular dark-field scanning TEM (HAADF-STEM) images are performed on JEOL JEM-ARM 200F. The atomic phase characterization of the material is performed on annular dark field scanning transmission electron microscopy (AC HAADF-STEM) images. X-ray powder diffraction (XRD) patterns are tested on Rigaku D/max-RC diffractometer under Cu Kα radiation (λ=0.15406 nm). X-ray photoelectron spectroscopy (XPS) is carried out on a Thermo VG Scientific ESCALAB 250 spectrometer with an Al Kα light source. Raman tests are conducted on a Raman spectrometer (Lab RAM HR800, λ=514 nm). The low-temperature N_2_ sorption measurements are investigated on a Micromeritics ASAP 2050 system. The metal content in the catalyst is determined by inductively coupled plasma mass spectrometry. Specifically, the metal content in the catalyst is quantified by inductively coupled plasma mass spectrometry (ICP-OES, Agilent 5900). To ensure complete dissolution, 100 mg of sample is dissolved in a mixture of 5 mL HNO_3_, 1 mL HF, 1 mL H_2_O_2_ and 1 ml HCl and heated under 200 ℃ for 8 h. The Fe K-edge XANES and extended XAFS (EXAFS) spectra are measured at the Deep-Inspectra-X1(Beijing SciStar Technology Co., Ltd.) under the transmission mode. Then, the Athena and Artemis software are used to analyze the collected data according to standard procedures. The magnetic properties are tested at the vibrating sample magnetometer (VSM) (Lake Shore Cryotronics, Inc.) within the range of 2T under room temperature.

*Electrochemical Testing*

All electrochemical measurements are performed on a CHI 760E electrochemical workstation equipped with a Pine Instruments high-speed rotator with a three-electrode setup, which are composed of a graphite rod as the counter electrode, a rotating ring-disk electrode (RRDE) as the working electrode, and a saturated calomel electrode (SCE) as the reference electrode in 0.1 M KOH solution. The catalyst-ink is prepared by ultrasonically treating the catalyst powder (2 mg) dispersed 400 μL of Nafion-mixed solution. Afterward, the 20 μL of as-prepared catalyst ink is dropped on the polished electrode surface. The linear sweep voltammetry (LSV) curves are conducted in O_2_-saturated 0.1 M KOH solution with the potential from range of 0 to 1.1 V_RHE_ in the ORR test. The electron-transfer number during the ORR is calculated out by the Koutecky-Levich plots at different potentials.

Koutecky-Levich equation:

$$\frac{\text{1}}{\text{j}}\text{=}\frac{\text{1}}{\text{j}_{\text{L}}}\text{+}\frac{\text{1}}{\text{j}_{\text{K}}}\text{=}\frac{\text{1}}{\text{0.62n}\text{F}\text{C}_{\text{0}}\text{D}_{\text{0}}^{\frac{\text{2}}{\text{3}}}\text{v}^{\frac{\text{-1}}{\text{6}}}\text{w}^{\frac{\text{1}}{\text{2}}}}\text{+}\frac{\text{1}}{\text{n}\text{F}\text{k}\text{C}_{\text{0}}}$$

where *j*, *j*_K_, and *j*_L_ are the measured current density, kinetic current density and limiting diffusion current density, *n* is the electron transfer number, *F* is the Faraday constant (96485 C mol^-1^), *C*_0_ is the concentration of O_2_ in the solution (1.14×10^-3^ mol L^-1^ for 0.1 M KOH), *D*_0_ is the diffusion coefficient of O_2_ (1.73×10^-5^ cm^2^ s^-1^ for 0.1 M KOH), *v* is the viscosity coefficient of the electrolyte (1.1×10^-2^ cm^2^ s^-1^ for 0.1 M KOH), and *w* is the electrode rotating speed. Additionally, the rotating speed is set as 1600 rpm and the scan rate was fixed at 5 mV s^-1^ and the H_2_O_2_ yield is calculated using the following equation:

$$\text{\%}\text{H}_{\text{2}}\text{O}_{\text{2}}\text{=}\frac{\text{200 }\text{I}_{\text{R}}}{\text{N }\text{I}_{\text{D}}\text{+}\text{I}_{\text{R}}}\text{=}\frac{\text{4N }\text{I}_{\text{D}}}{\text{N}\text{ I}_{\text{D}}\text{+}\text{I}_{\text{R}}}$$

where *I*_R_ and *I*_D_ stand for the ring current density and disk current density, respectively. *N* is the collecting efficiency of 0.37. The chronoamperometry tests are performed at 0.7 V. The electrochemical impedance spectroscopy (EIS) is measured in O_2_-saturated 0.1 M KOH at the open voltage from 10 kHz to 0.1 Hz without rotating speed.

*Electrochemical In-Situ Enhanced Raman Measurements*

The Raman spectra are collected using a Labram HR800 (532 nm laser) inverted Raman spectroscopy system. The *in-situ* surface-enhanced Raman spectra are performed in a Raman cell (Beijing Scistar Technology Co., Ltd.) with Au@SiO_2_ + samples decorated on a GC electrode (working electrode), Pt wire (counter electrode), and Ag/AgCl (saturated KCl) (reference electrode), respectively, in 0.1 M KOH solution. The CHI 760E electrochemical workstation is used to control the potential, which ranges from 1.0 to 0.1 V vs. RHE.

*Electrochemical In-situ ATR-SEIRAS Measurements*

The *in-situ* attenuated total reflection-surface-enhanced IR absorption spectroscopy (ATR-SEIRAS) is performed by a Bruker INVENIO R FT-IR spectrometer equipped with an MCT detector cooled with liquid nitrogen. The catalyst is dropped on hemispherical silicon crystal deposited chemically with Au film, which is used as working electrode. A Pt foil is used as the counter electrode and an Ag/AgCl electrode (saturated with KCl) is used as a reference electrode. Real-time FTIR spectra are recorded during the chronopotentiometry test. All spectra are expressed in absorbance unit as -log (*I*/*I*_0_) with *I* and *I*_0_ representing the sample and reference spectra.

*DFT Calculations*

We performed all density functional theory (DFT) calculations using the Vienna *Ab initio* Simulation Package (VASP)^[1, 2]^, within the generalized gradient approximation (GGA) and employed the Revised Perdew Burke Ernzerhof (RPBE) exchange correlation functional^[3]^. The projected augmented wave (PAW) method was used to describe the ionic cores^[4,5]^, and the valence electrons were expanded in a plane wave basis with a kinetic energy cutoff of 450 eV. Van der Waals interactions were included using the DFT-D3 empirical dispersion correction^[6]^. Geometry optimizations were carried out until the residual forces on each atom were below 0.05 eV Å^-1^ and the total energy change between successive steps was less than 1 × 10^-5^ eV. The bottom atomic layer of the slab was fixed during all calculations. Brillouin zone integrations were performed using a Gamma centered (2 × 2 × 1) k point mesh. To properly treat localized electronic states, the DFT plus U method was applied to Fe 3d and Sm 4f orbitals with effective U-J values of 5.3 eV and 4.7 eV, respectively^[7,8]^. Spin polarization was included throughout. Free energy changes (ΔG) for each elementary ORR step were evaluated using the computational hydrogen electrode (CHE) model^[9]^, where the chemical potential of a proton electron pair at 0 V vs. RHE equals half of the total energy of gas phase H_2_. The effect of electrode potential U (vs. RHE) was incorporated by adding-eU for each transferred electron, that is, G (U) = G (0 V) -neU, where e is the elementary charge, n is the number of transferred proton electron pairs, and U is the applied potential. The Gibbs free energy was calculated using the following equation: *G* = *E*_DFT_ + *E*_ZPE_ – *TS*, where E is the total energy, ZPE is the zero-point energy, T is the temperature (set at 298.15 K), and S is the entropy.

*Microkinetic Modeling of ORR*

The microkinetic modeling of the ORR volcano was based on the approach outlined by our team using the Digital Catalysis Platform (*DigCat*: www.digcat.org)^[10]^. Rates for intermediate steps were calculated using Equation:

$$\text{r} = \text{k}_{\text{f}} \text{П}\text{ }\text{θ}_{\text{reac}} \text{-} \text{k}_{\text{r}}{\text{ }\text{П}\text{ }\text{θ}}_{\text{prod}}$$

where *r* denotes the reaction rate, and *θ*_reac_ and *θ*_prod_ represent the surface coverages of the reactant and product species, respectively.

The rate constant *k* was computed within transition-state theory as a function of the prefactor A (s^-1^), activation free energy *G*_a_, Boltzmann constant *k*_B_, and reaction temperature *T*:

$$\text{k}\text{ }\text{=}\text{ }\frac{\text{k}_{\text{B}}\text{T}}{\text{h}} \text{e}^{\text{-}\frac{\text{G}_{\text{a}}}{\text{k}_{\text{B}}\text{T}}}\text{ }\text{=}\text{ }\text{A}{\text{ }\text{e}}^{\text{-}\frac{\text{G}_{\text{a}}}{\text{k}_{\text{B}}\text{T}}}$$

The elementary reaction steps considered in the microkinetic model are listed below:

O (aq) →O (dl)

O_2_ (dl) + * → O_2_*

O_2_* + H^+^ + e^-^ → HOO*

HOO* + H^+^ + e^-^ → O* + H_2_O (l)

O* + H^+^ + e^-^ → HO*

HO* + H^+^ + e^-^ → H_2_O (l) + *

HOO* + H^+^ + e^-^ → H_2_O_2_*

H_2_O_2_* → H_2_O_2_ (aq)+ *

For the O-O scission event that occurs concurrently with protonation, we adopted the activation-energy expression proposed by Dickens et al^[11]^. for O-O bond cleavage:

$$\text{G}_{\text{TS}}\text{ }\text{=}\text{ }\text{0.99}{\text{ }\text{G}}_{\text{HOO}\text{*}}\text{-}\text{0.25}\text{ }\text{+}\text{ }\text{0.42}\text{ }\text{U}_{\text{RHE}}$$

For all other proton-transfer events that do not involve additional bond breaking, an intrinsic barrier of 0.26 eV was used, and 0.5 electrons were assumed to be transferred at the transition state^[12]^. Perfectors for all proton–electron transfer events were set to 10^-9^ s^-1^ to account for solvent reorganization^[13]^. Complete details and considered parameters can be found in Refs.^[10,14,15]^.

*Analysis method of* *Temperature-dependent magnetization measurements*

Since the as-synthesized materials in this work does not satisfy the applicability criteria of the standard coupled two-sublattice model, the corresponding theoretical approach cannot be used to directly extract the exchange coupling constant (*J*). Nevertheless, given that the Curie-Weiss Law is a widely accepted method in magnetic studies, it is adopted here for the qualitative analysis of the experimental data ^[16-17]^. In particular, because *J* $\propto$ Weiss temperature *θ*, the *θ* derived from fitting resulting can serve as an indirect indicator for comparing the relative variation of *J*. Meanwhile, the Curie constant C obtained simultaneously from the fitting can also provide complementary evidence for evaluating the magnitude of the magnetic moment in the material.

Curie-Weiss Law:

$$\chi\text{ = }\mathrm{C}\text{/ (}T-\theta\text{)}$$

Where C is the Curie constant, χ is the magnetic susceptibility, *θ* is the Weiss temperature. According to this equation, the high-temperature region from 100 to 275 K is selected for first-order linear fitting, from which the corresponding slope and intercept are obtained, then the relevant physical parameters are subsequently derived.

*Liquid Zinc-Air Batteries Testing*

The rechargeable Zinc-air batteries (ZABs) are composed of a thickness of 0.3 mm polished zinc sheet as anode and hydrophilic carbon paper coated with 40 mg mL^-1^ catalyst and nickel foam as air cathode. The electrolyte consists of 6 M KOH + 0.2 M ZnCl_2_ mixed aqueous solution. The as-assembled ZABs are tested by setting the current density (5 mA cm^-2^) and cycle of each charge and discharge (20 min) in the Land CT2001A system. Meanwhile, the corresponding specific capacity (mAh g_Zn_^-1^) and energy density (Wh kg_Zn_^-1^) can be calculated according to the discharge curve when the constant current density is 5 mA cm^-2^. The calculation formula is as follows:

Specific capacity = current × service hours/weight of consumed Zn

Energy density = current × service hours × average discharge voltage/weight of consumed Zn

*All Solid-State Zinc-Air Batteries Performance Evaluation*

Using the same structure of the aqueous Zinc-Air battery, all solid-state Zinc-Air batteries are assembled by a polished zinc foil (0.2 mm), a piece of Ni foam with a catalyst loading of 1.0 mg cm^-2^ and a solid electrolyte. The battery performance measurements are basically the same as the aqueous Zinc-Air battery tests, except that the charge-discharge current density is reduced to 1 mA cm^-2^.

**Figures S1-S25**


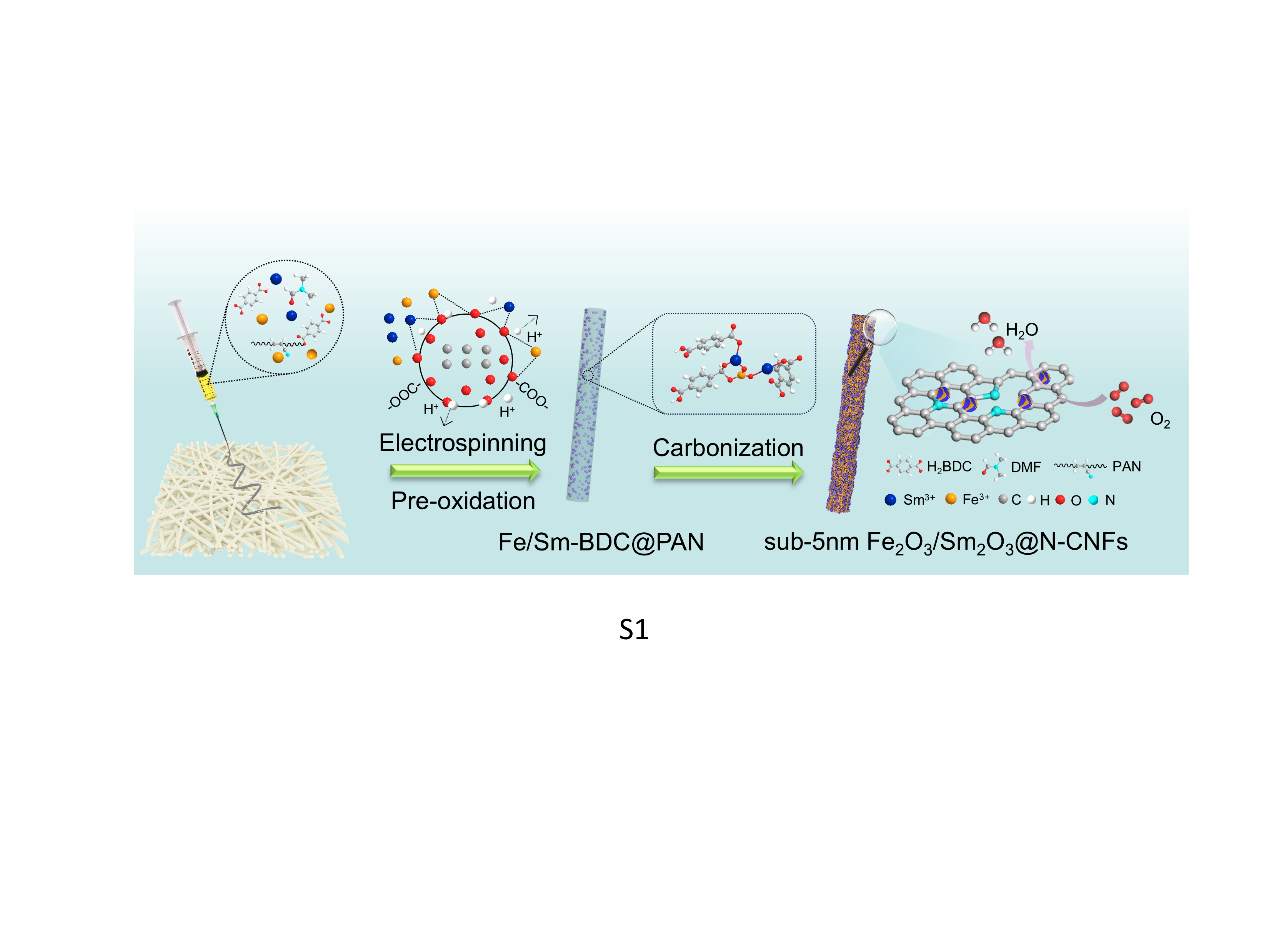


**Figure S1.** Schematic illustration of the fabrication procedure of the sub-5nm Fe_2_O_3_/Sm_2_O_3_@N-CNFs.


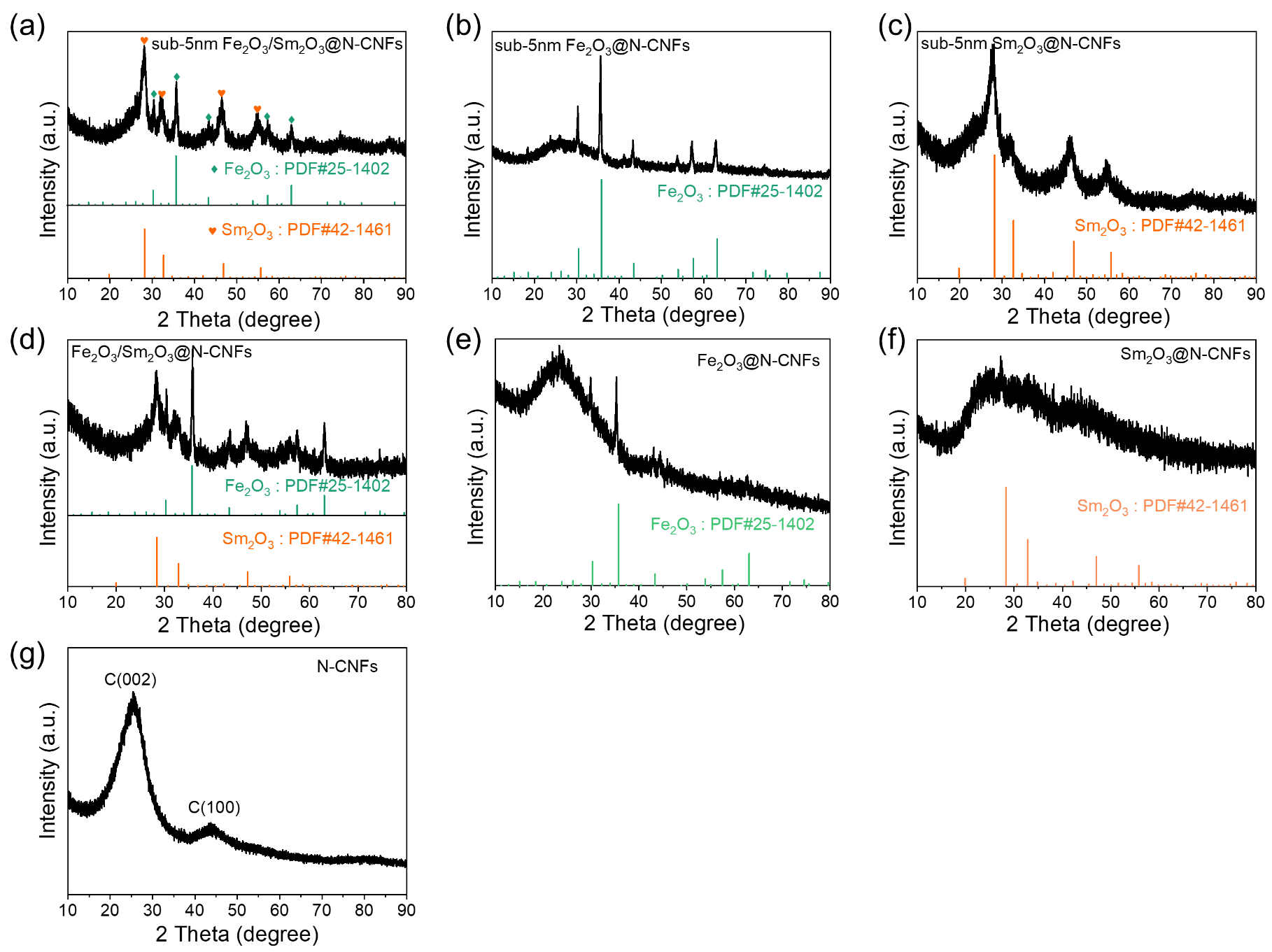


**Figure S2.** XRD patterns of all materials. (a) sub-5nm Fe_2_O_3_/Sm_2_O_3_@N-CNFs, (b) sub-5nm Fe_2_O_3_@N-CNFs, (c) sub-5nm Sm_2_O_3_@N-CNFs, (d) Fe_2_O_3_/Sm_2_O_3_@N-CNFs, (e) Fe_2_O_3_@N-CNFs, (f) Sm_2_O_3_@N-CNFs, and (g) N-CNFs.


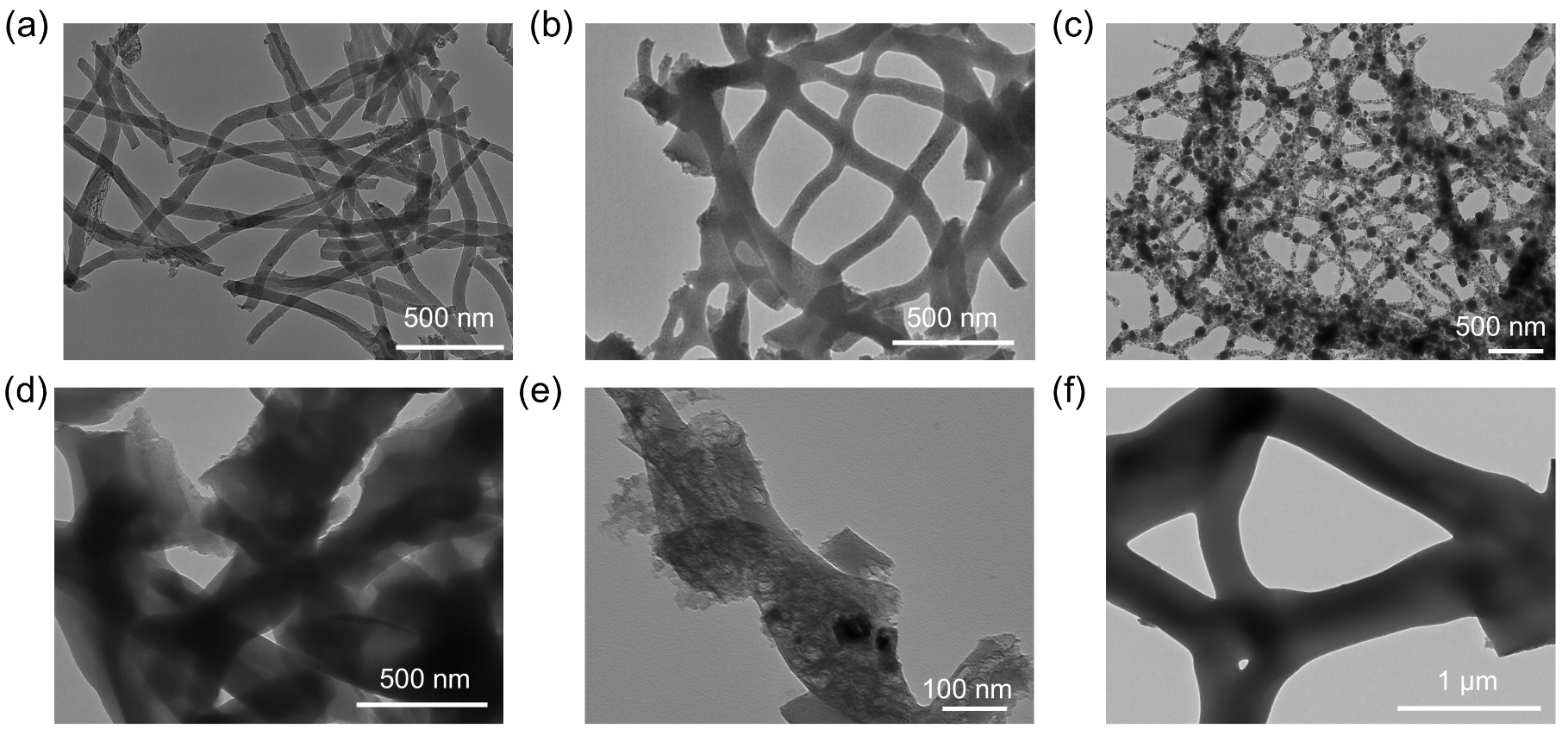


**Figure S3.** TEM images of reference materials. (a) sub-5nm Fe_2_O_3_@N-CNFs, (b) sub-5nm Sm_2_O_3_@N-CNFs, (c) Fe_2_O_3_/Sm_2_O_3_@N-CNFs, (d) Fe_2_O_3_@N-CNFs, (e) Sm_2_O_3_@N-CNFs, and (f) N-CNFs.


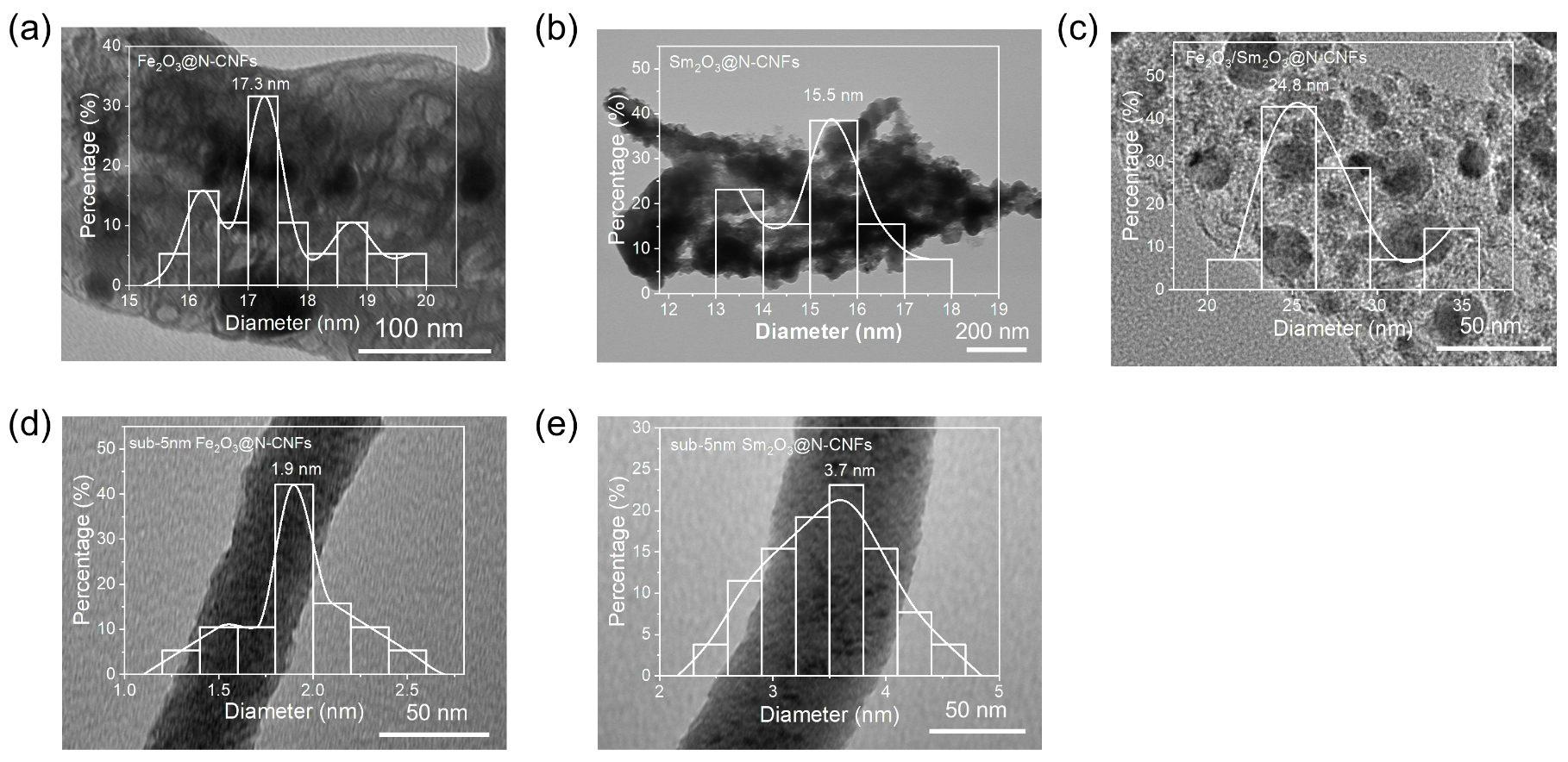


**Figure S4.** The particle size analysis of reference materials. (a) Fe_2_O_3_@N-CNFs, (b) Sm_2_O_3_@N-CNFs, (c) Fe_2_O_3_/Sm_2_O_3_@N-CNFs, (d) sub-5nm Fe_2_O_3_@N-CNFs, and (e) sub-5nm Sm_2_O_3_@N-CNFs.


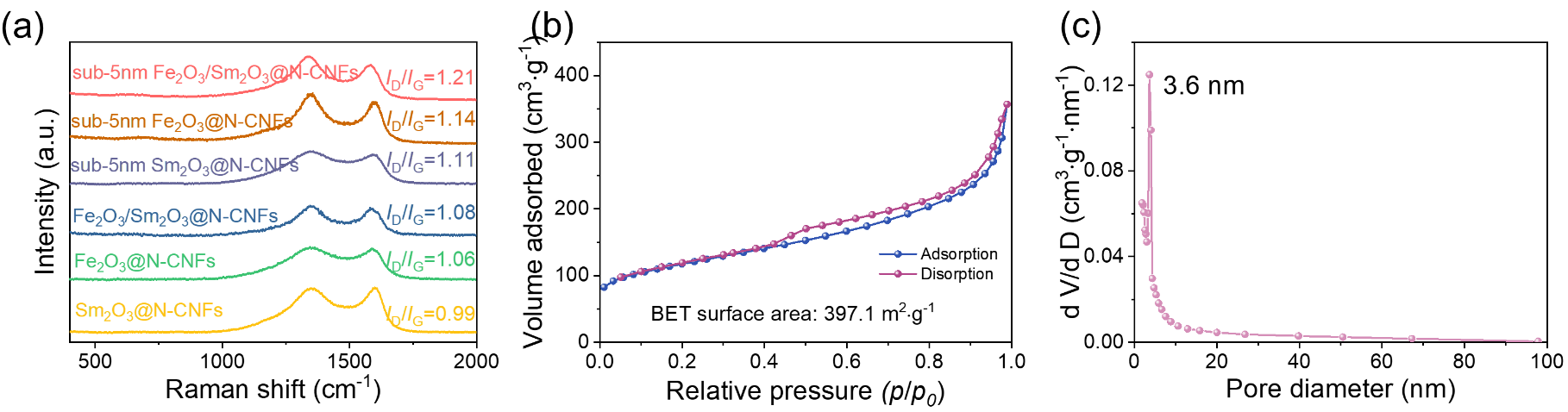


**Figure S5.** (a) the Raman spectrum of Fe_2_O_3_@N-CNFs, Sm_2_O_3_@N-CNFs, Fe_2_O_3_/Sm_2_O_3_@N-CNFs, sub-5nm Fe_2_O_3_@N-CNFs, sub-5nm Sm_2_O_3_@N-CNFs, and sub-5nm Fe_2_O_3_/Sm_2_O_3_@N-CNFs, (b) N_2_ adsorption-desorption isotherms of sub-5nm Fe_2_O_3_/Sm_2_O_3_@N-CNFs, and (c) the pore-size distribution curve of sub-5nm Fe_2_O_3_/Sm_2_O_3_@N-CNFs.


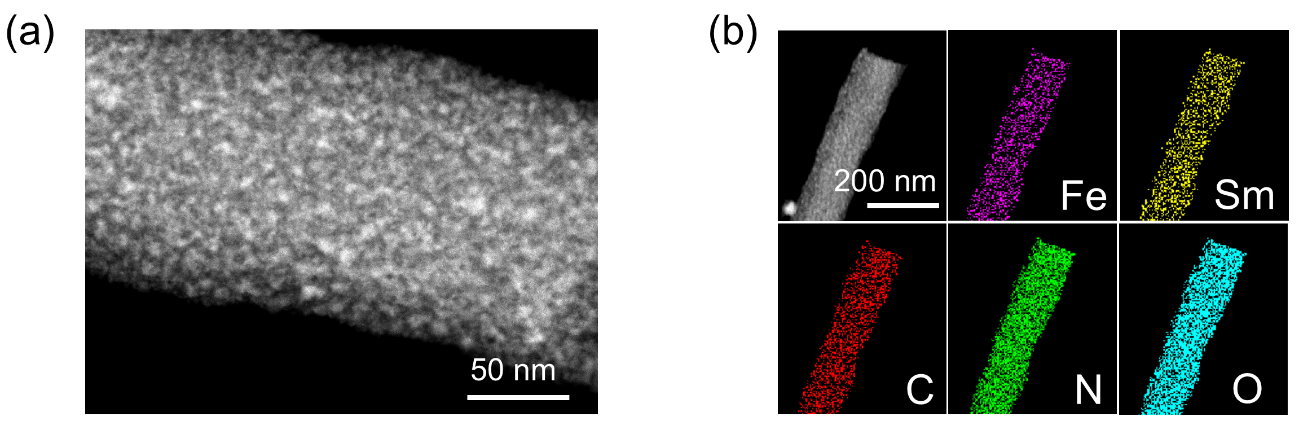


**Figure S6.** (a) AC-HAADF-STEM images of sub-5nm Fe_2_O_3_/Sm_2_O_3_@N-CNFs, (b) EDX element mapping of sub-5nm Fe_2_O_3_/Sm_2_O_3_@N-CNFs.


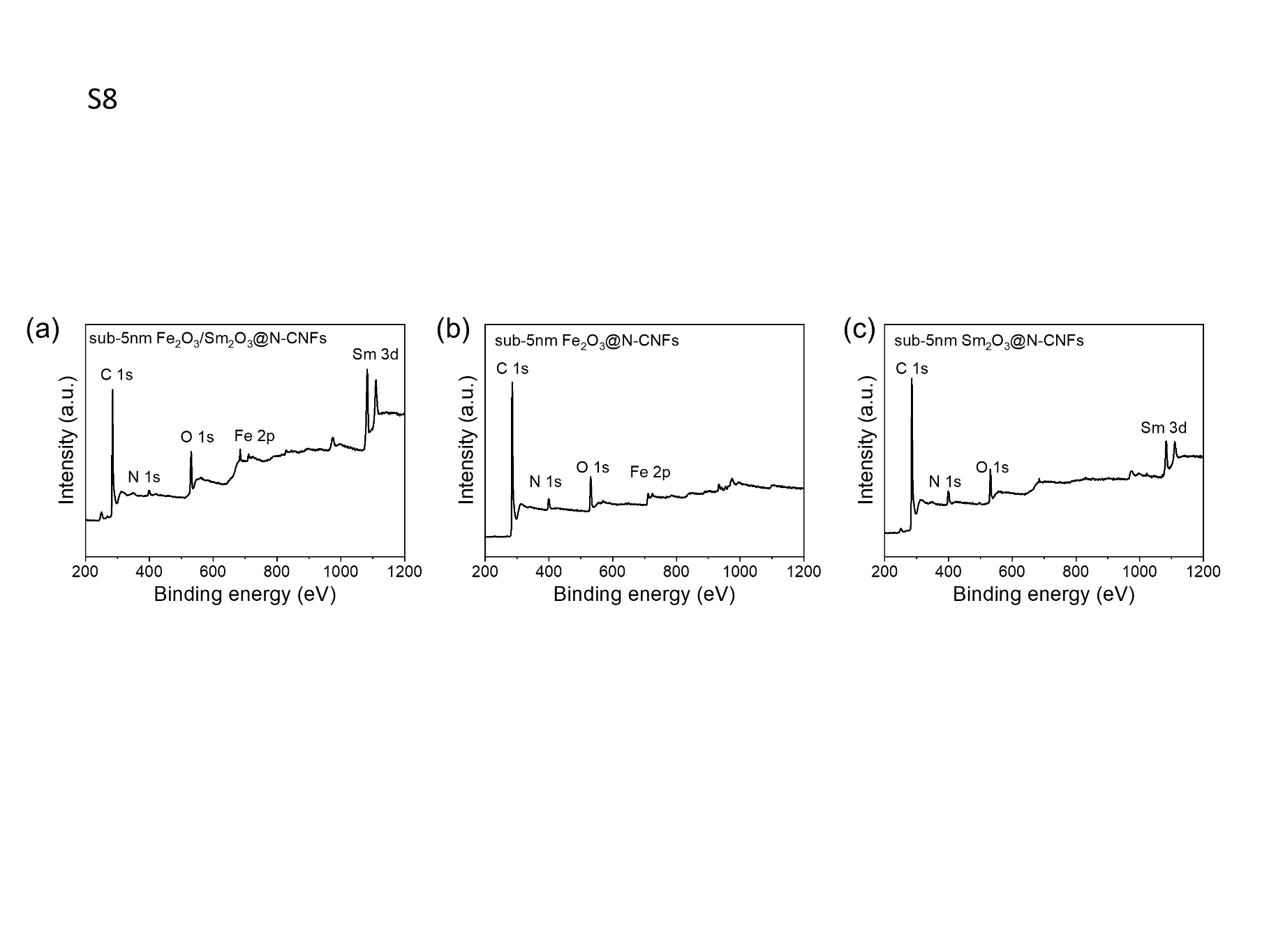


**Figure S7.** XPS survey spectrum of (a) sub-5nm Fe_2_O_3_/Sm_2_O_3_@N-CNFs, (b) sub-5nm Fe_2_O_3_@N-CNFs, and (c) sub-5nm Sm_2_O_3_@N-CNFs.


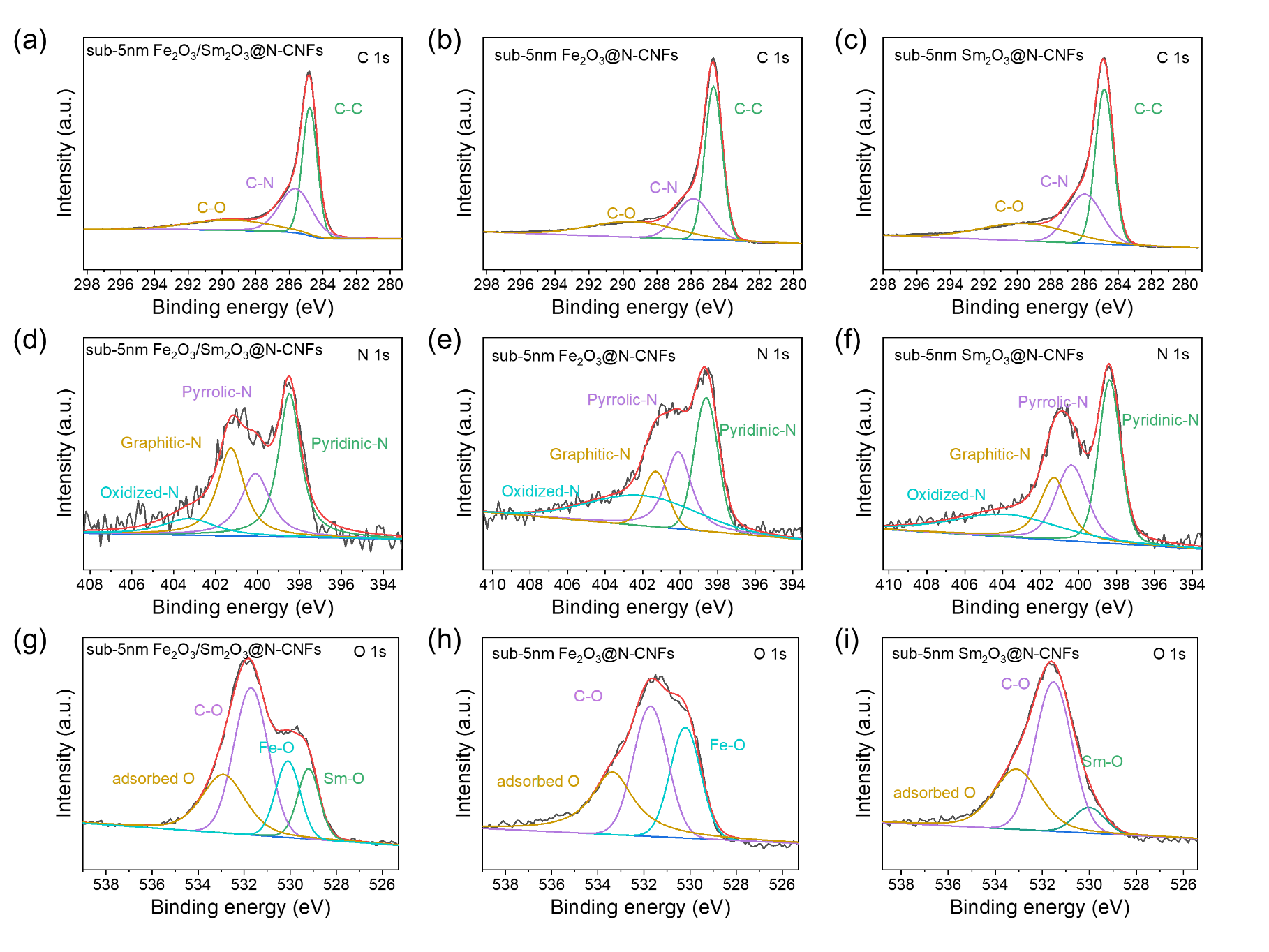


**Figure S8.** The high-resolution C 1s XPS spectrum of (a) sub-5nm Fe_2_O_3_/Sm_2_O_3_@N-CNFs, (b) sub-5nm Fe_2_O_3_@N-CNFs, and (c) sub-5nm Sm_2_O_3_@N-CNFs. The high-resolution N 1s XPS spectrum of (d) sub-5nm Fe_2_O_3_/Sm_2_O_3_@N-CNFs, (e) sub-5nm Fe_2_O_3_@N-CNFs, and (f) sub-5nm Sm_2_O_3_@N-CNFs. The high-resolution O 1s XPS spectrum of (g) sub-5nm Fe_2_O_3_/Sm_2_O_3_@N-CNFs, (h) sub-5nm Fe_2_O_3_@N-CNFs, and (i) sub-5nm Sm_2_O_3_@N-CNFs.


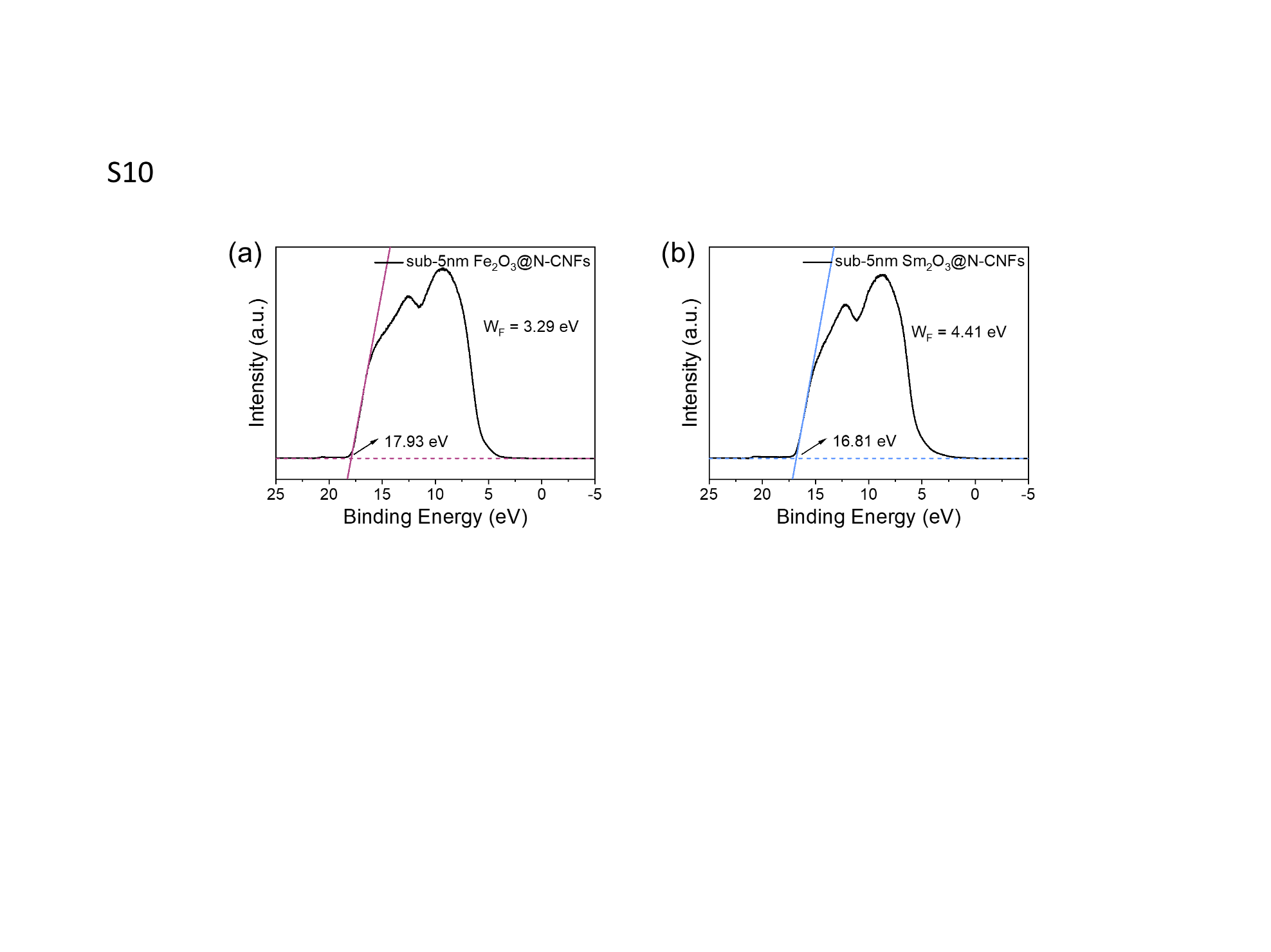


**Figure S9.** UPS spectrum of (a) sub-5nm Fe_2_O_3_@N-CNFs, and (b) sub-5nm Sm_2_O_3_@N-CNFs.


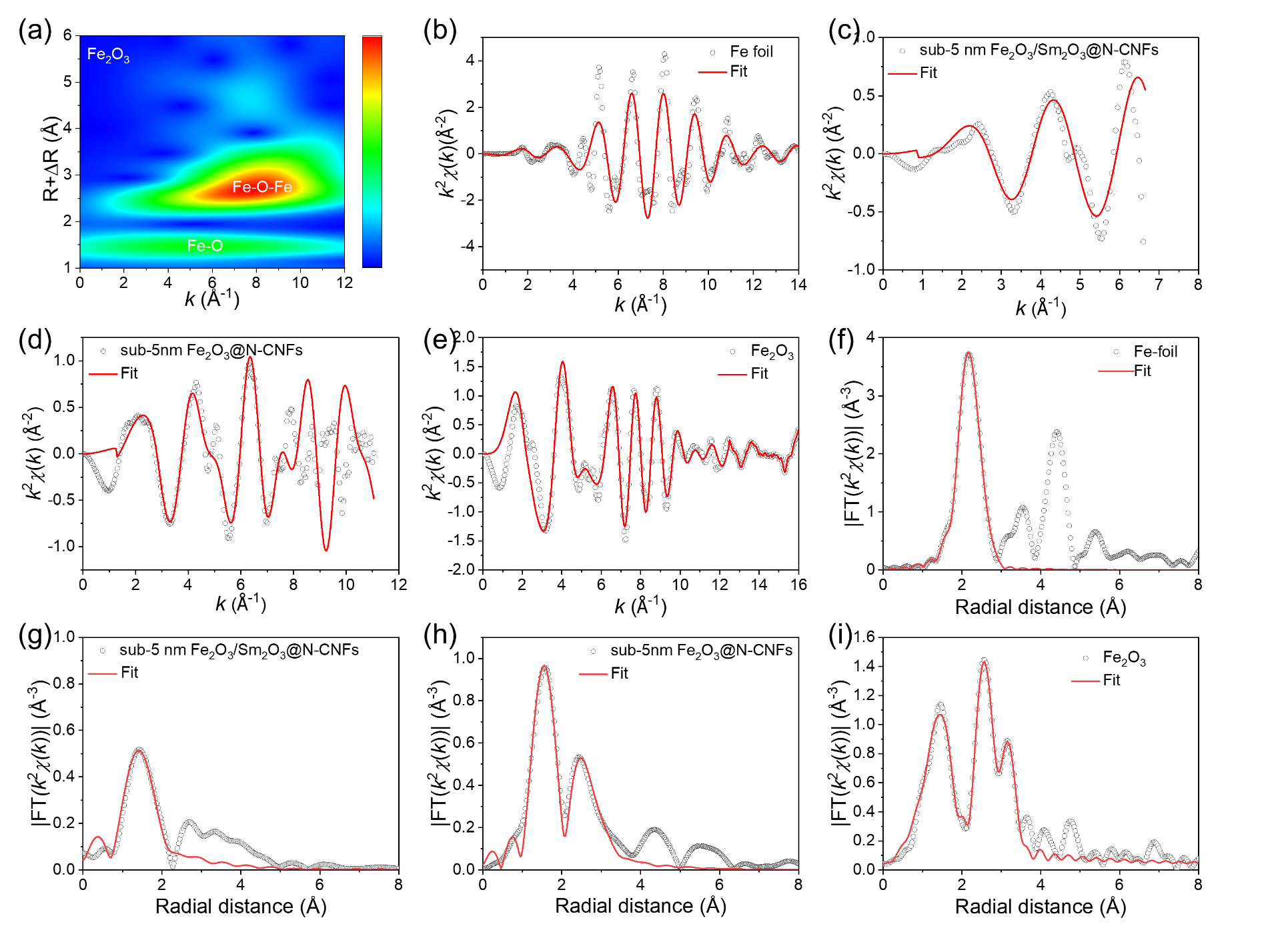


**Figure S10.** (a) Wavelet transform for the k^2^-weighted EXAFS contour plots of Fe element, Fe K-edge FT-EXAFS fitting analysis of (b) Fe foil in K-space, (c) sub-5nm Fe_2_O_3_/Sm_2_O_3_@N-CNFs in K-space, (d) sub-5nm Fe_2_O_3_@N-CNFs in K-space, (e) Fe_2_O_3_ in K-space, (f) Fe foil in R-space, (g) sub-5nm Fe_2_O_3_/Sm_2_O_3_@N-CNFs in R-space, (h) sub-5nm Fe_2_O_3_@N-CNFs in R-space, and (i) Fe_2_O_3_ in R-space.


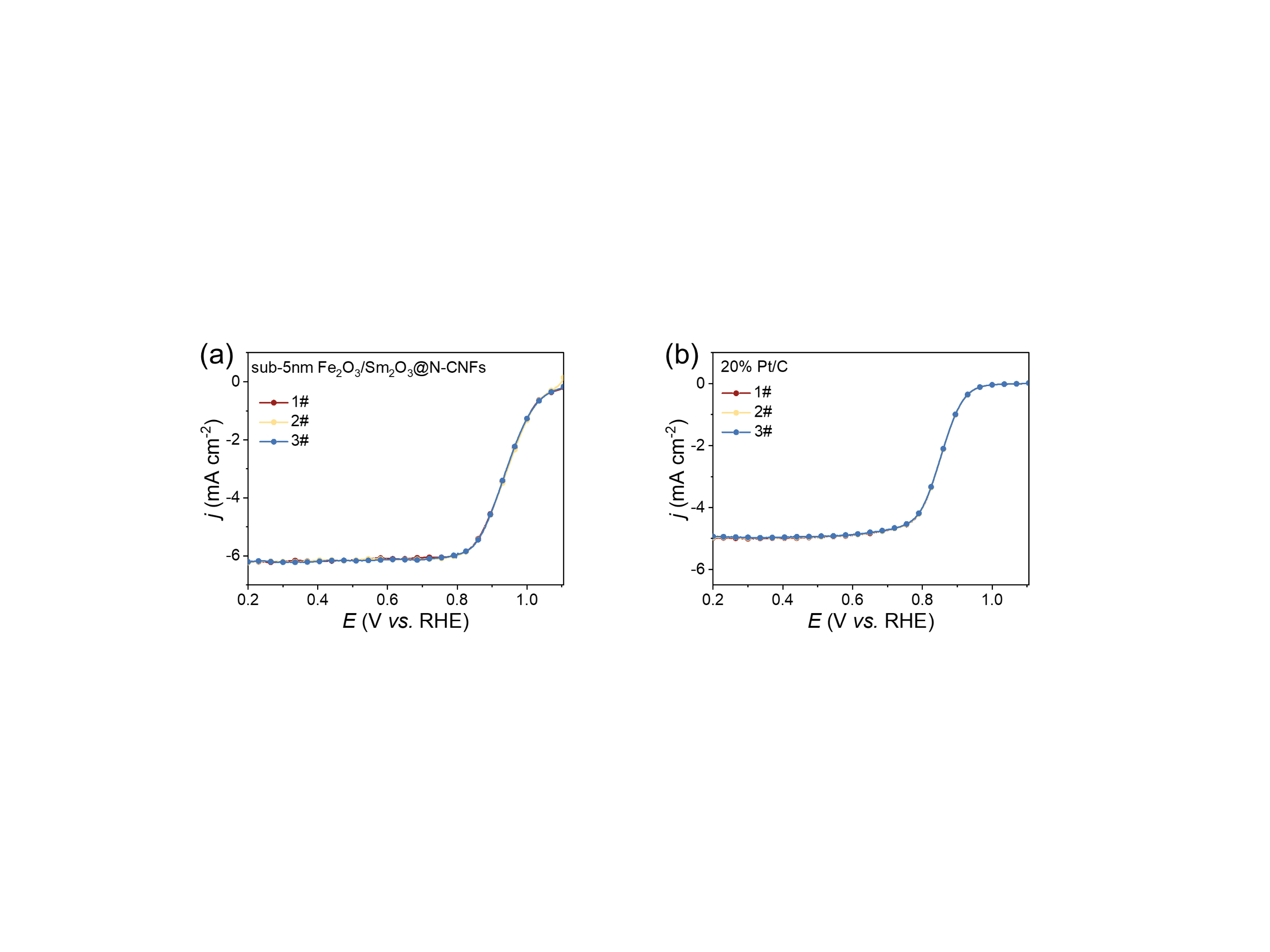


**Figure S11.** The three LSV curves under the same conditions: (a)sub-5nm Fe_2_O_3_/Sm_2_O_3_@N-CNFs, and (b) Pt/C.


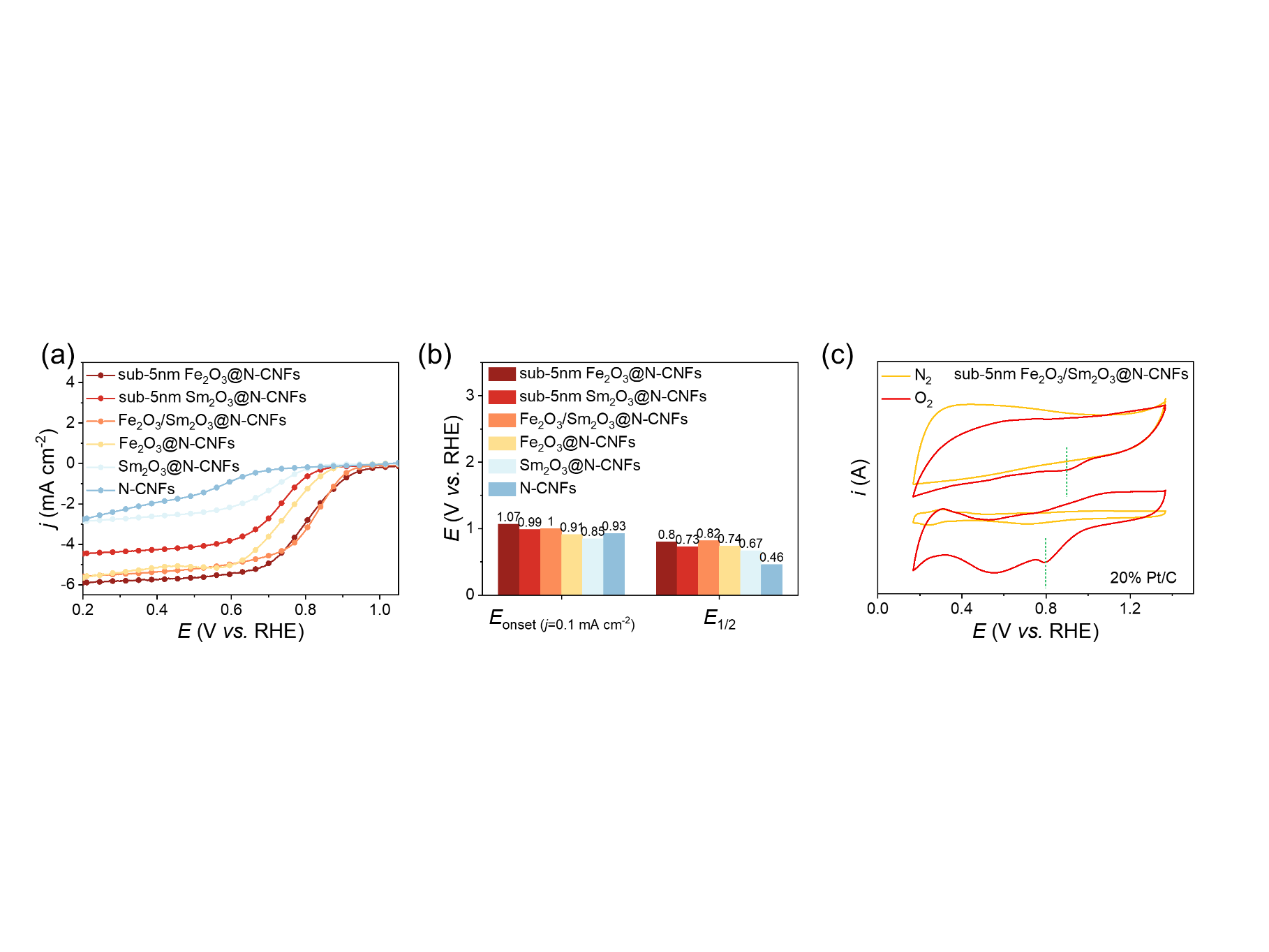


**Figure S12.** (a) the LSV curves of sub-5nm Fe_2_O_3_@N-CNFs, sub-5nm Sm_2_O_3_@N-CNFs, Fe_2_O_3_/Sm_2_O_3_@N-CNFs, Fe_2_O_3_@N-CNFs, Sm_2_O_3_@N-CNFs and N-CNFs, (b) performance comparison histogram of sub-5nm Fe_2_O_3_@N-CNFs, sub-5nm Sm_2_O_3_@N-CNFs, Fe_2_O_3_/Sm_2_O_3_@N-CNFs, Fe_2_O_3_@N-CNFs, Sm_2_O_3_@N-CNFs and N-CNFs. and (c) N_2_ and O_2_-saturated CV curves of sub-5nm Fe_2_O_3_/Sm_2_O_3_@N-CNFs and Pt/C.


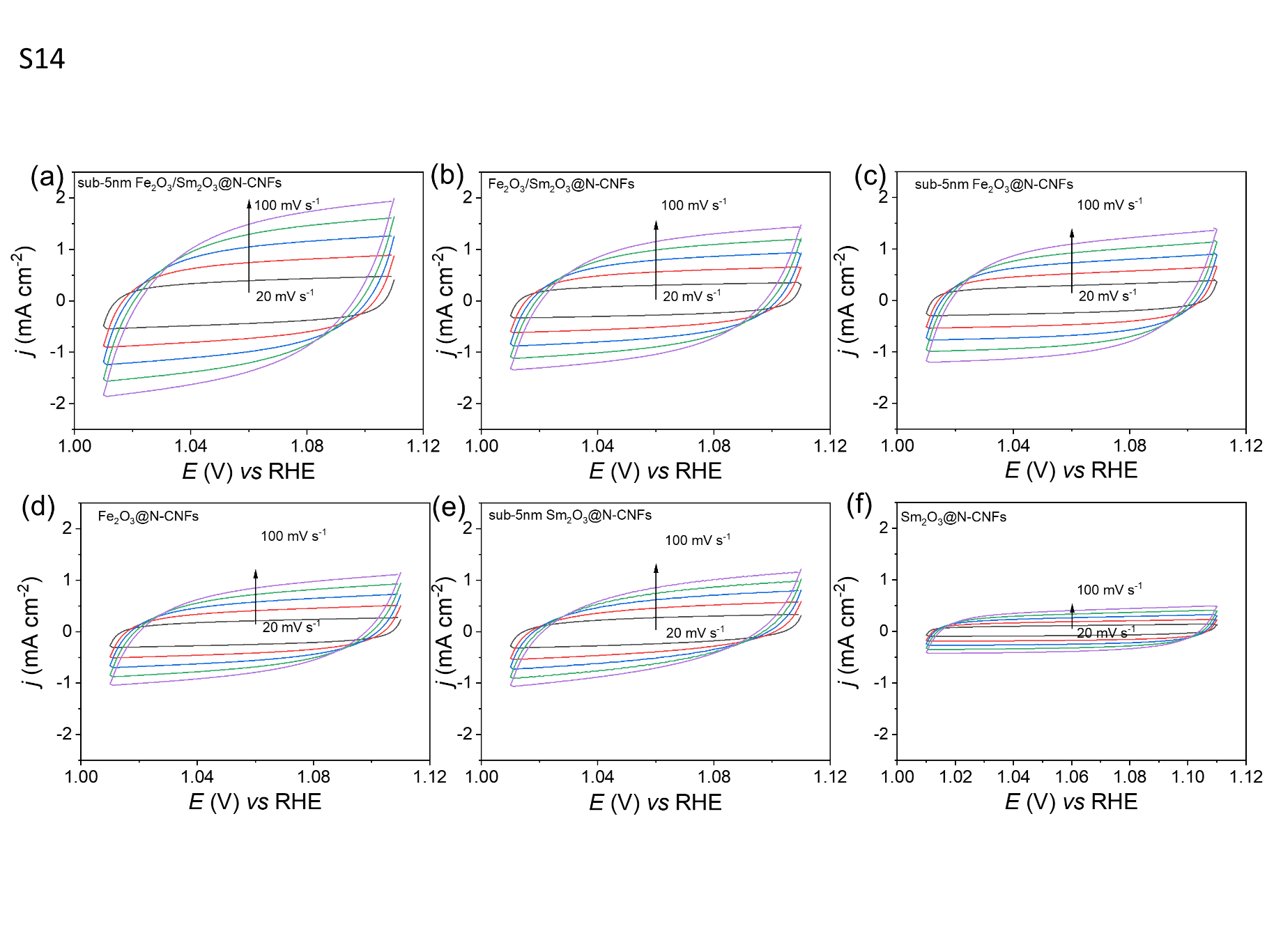


**Figure S13.** CV curves of the synthesized catalysts in the non-Faradaic region obtained at different scanning rates. (a) sub-5nm Fe_2_O_3_/Sm_2_O_3_@N-CNFs, (b) Fe_2_O_3_/Sm_2_O_3_@N-CNFs, (c) sub-5nm Fe_2_O_3_@N-CNFs, (d) Fe_2_O_3_@N-CNFs, (e) sub-5nm Sm_2_O_3_@N-CNFs, (f) Sm_2_O_3_@N-CNFs.


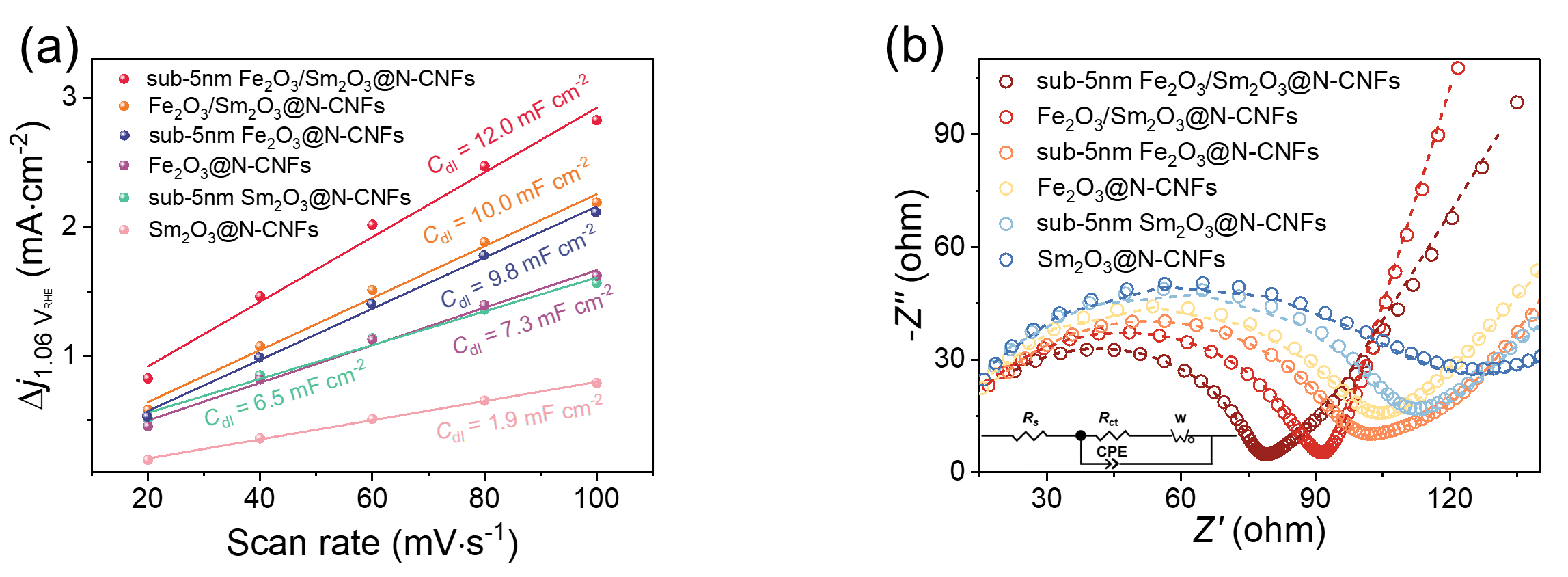


**Figure S14.** (a) Estimated *C*_dl_ values, (b) EIS Nyquist plots and corresponding fitting results of sub-5nm Fe_2_O_3_/Sm_2_O_3_@N-CNFs, Fe_2_O_3_/Sm_2_O_3_@N-CNFs, sub-5nm Fe_2_O_3_@N-CNFs, Fe_2_O_3_@N-CNFs, sub-5nm Sm_2_O_3_@N-CNFs, and Sm_2_O_3_@N-CNFs (inset: simulated equivalent circuit).


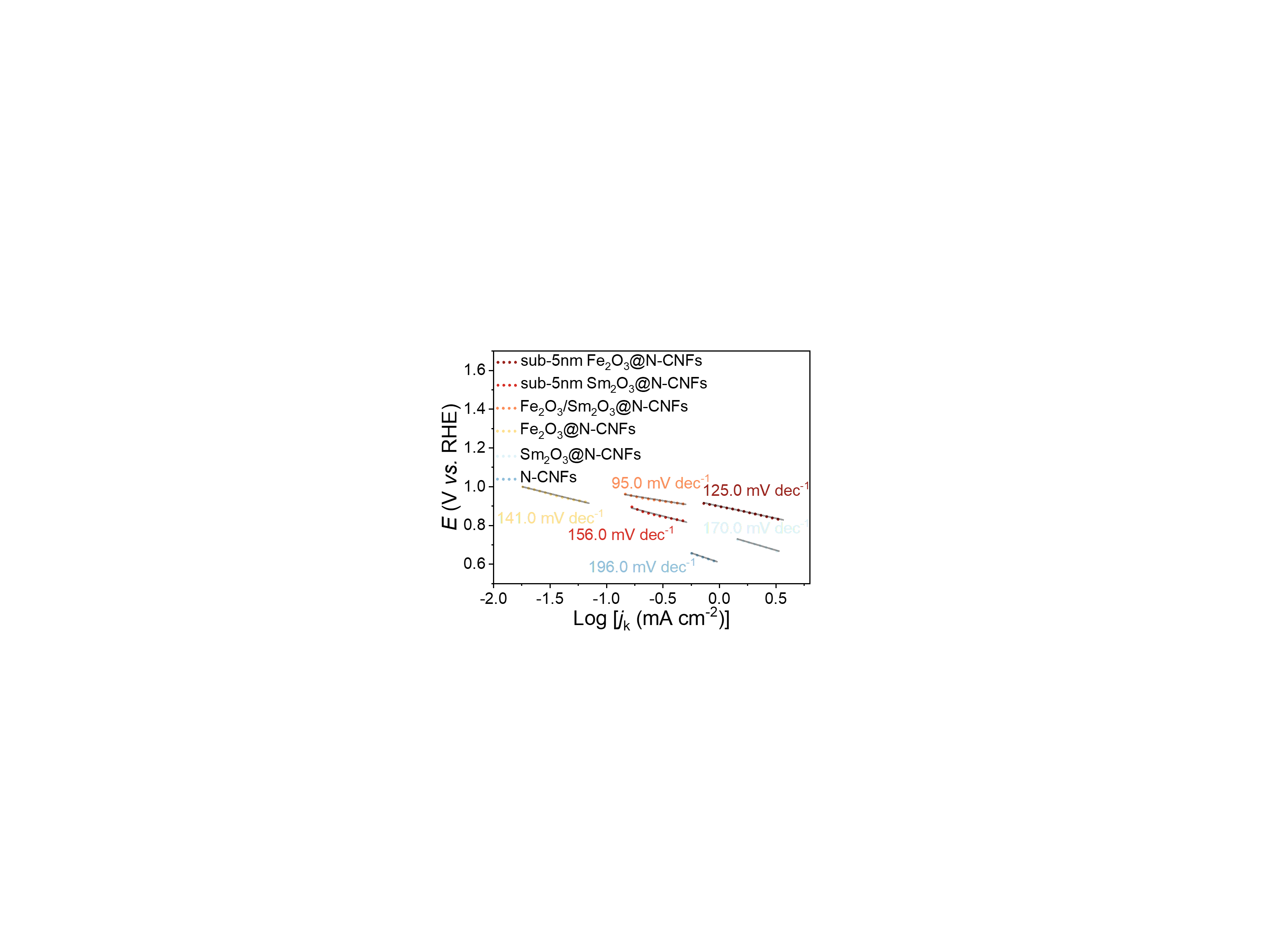


**Figure S15.** The Tafel slope of sub-5nm Fe_2_O_3_@N-CNFs, sub-5nm Sm_2_O_3_@N-CNFs, Fe_2_O_3_/Sm_2_O_3_@N-CNFs, Fe_2_O_3_@N-CNFs, Sm_2_O_3_@N-CNFs, and N-CNFs.


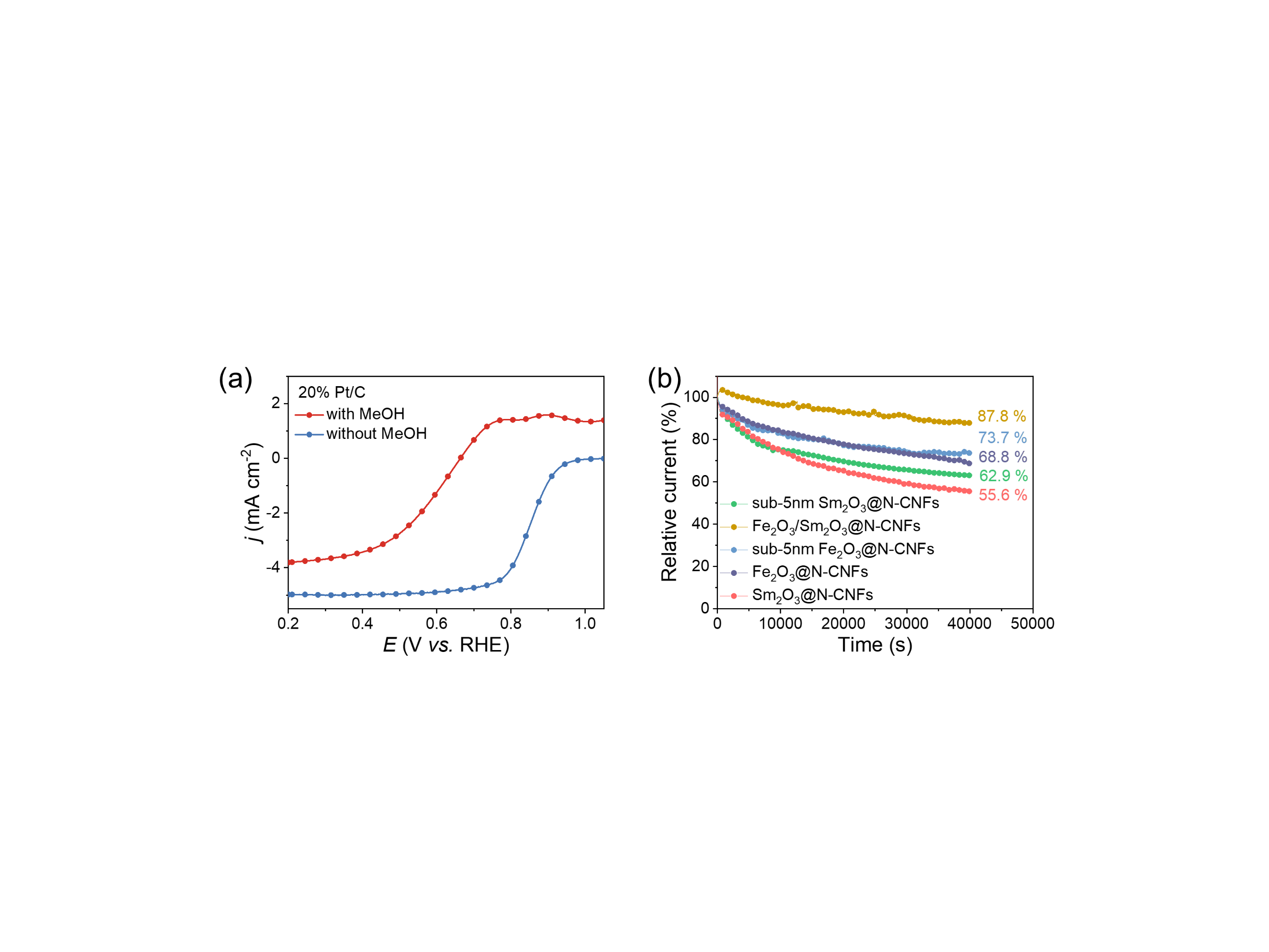


**Figure S16.** (a) methanol poisoning tolerance test of Pt/C, and (b) chronoamperometry curves of Fe_2_O_3_/Sm_2_O_3_@N-CNFs, sub-5nm Fe_2_O_3_@N-CNFs, Fe_2_O_3_@N-CNFs, sub-5nm Sm_2_O_3_@N-CNFs and Sm_2_O_3_@N-CNFs at 0.6 V vs. RHE.


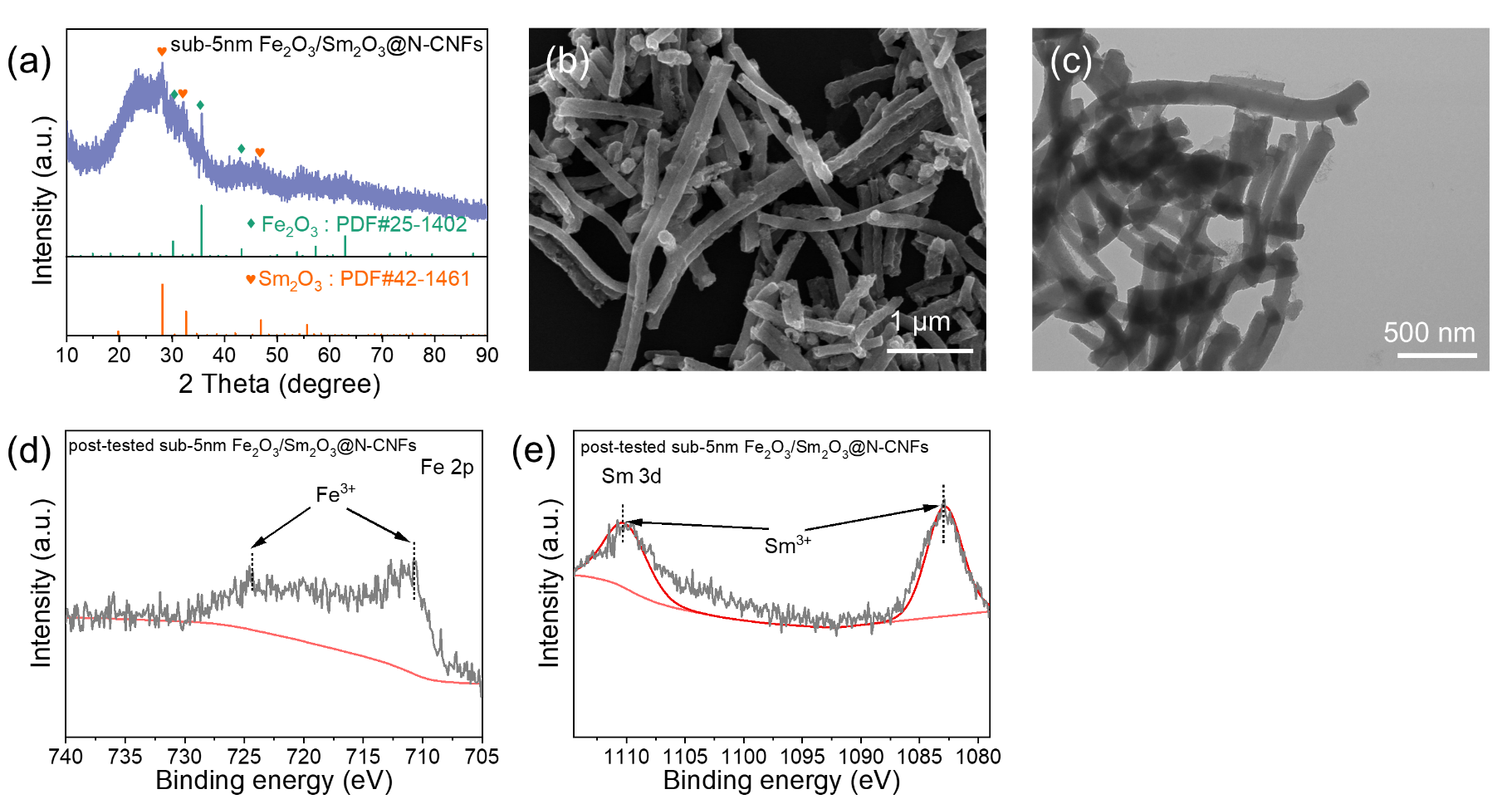


**Figure S17.** (a) XRD pattern, (b) SEM image, (c) TEM image, (d) Fe 2p spectrum, and (e) Sm 3d spectrum of sub-5nm Fe_2_O_3_/Sm_2_O_3_@N-CNFs after the stability test.


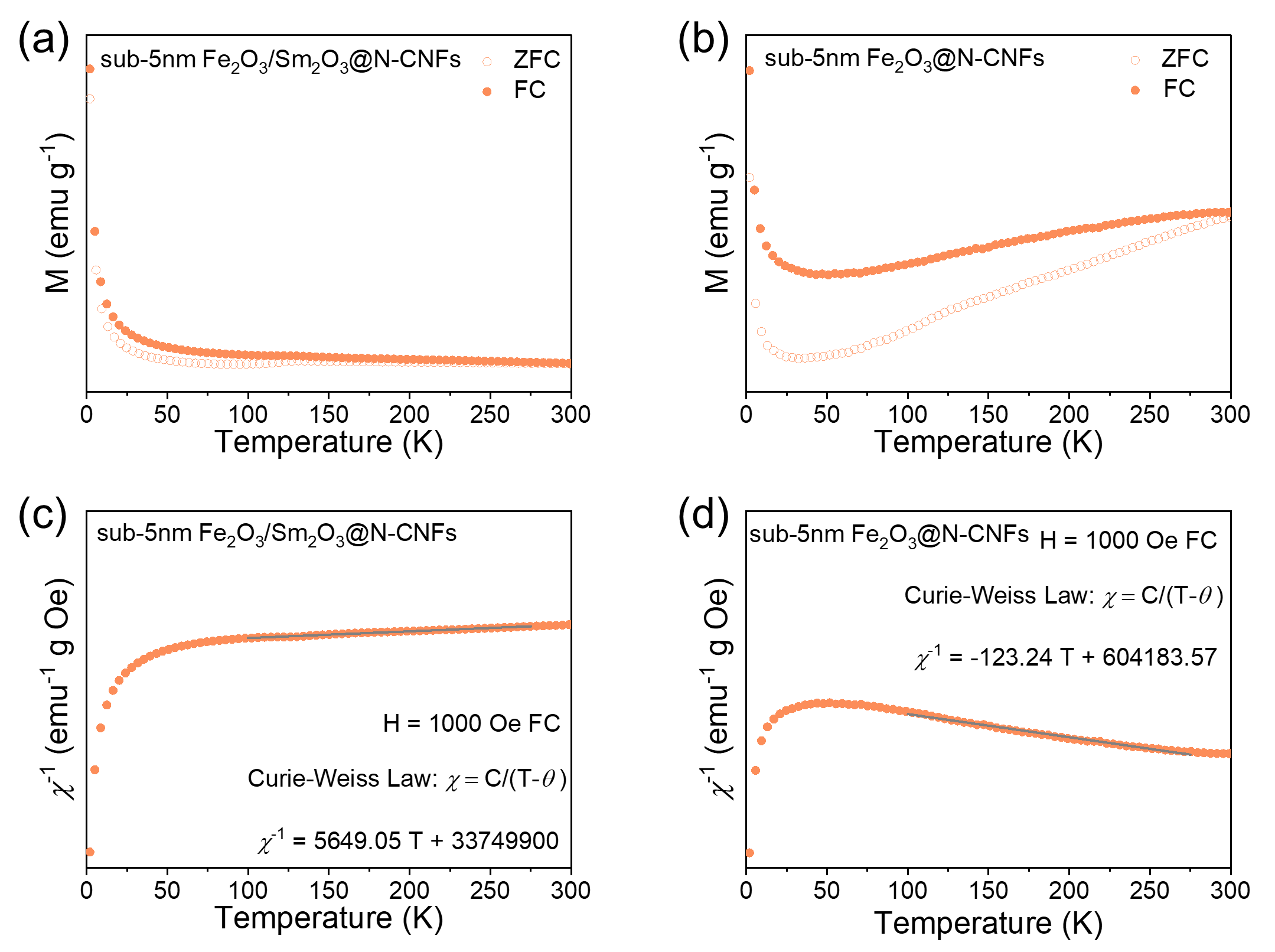


**Figure S18.** Temperature-dependent magnetization (M/T) measurements of sub-5nm Fe_2_O_3_/Sm_2_O_3_@N-CNFs and sub-5nm Fe_2_O_3_@N-CNFs: (a) M/T curves of sub-5nm Fe_2_O_3_/Sm_2_O_3_@N-CNFs in FC and ZFC (FC: field-cooled at a magnetic field of H = 1000 Oe, ZFC: zero field-cooled), (b) M/T curves of sub-5nm Fe_2_O_3_@N-CNFs in FC and ZFC, the analyses of temperature-dependent magnetic susceptibility (χ/T) in FC at a magnetic field of H = 1000 Oe: (c) χ^-1^/T curve of sub-5nm Fe_2_O_3_/Sm_2_O_3_@N-CNFs, and (d) χ^-1^/T curve of sub-5nm Fe_2_O_3_@N-CNFs (C: Curie constant, *θ*: Weiss temperature.)


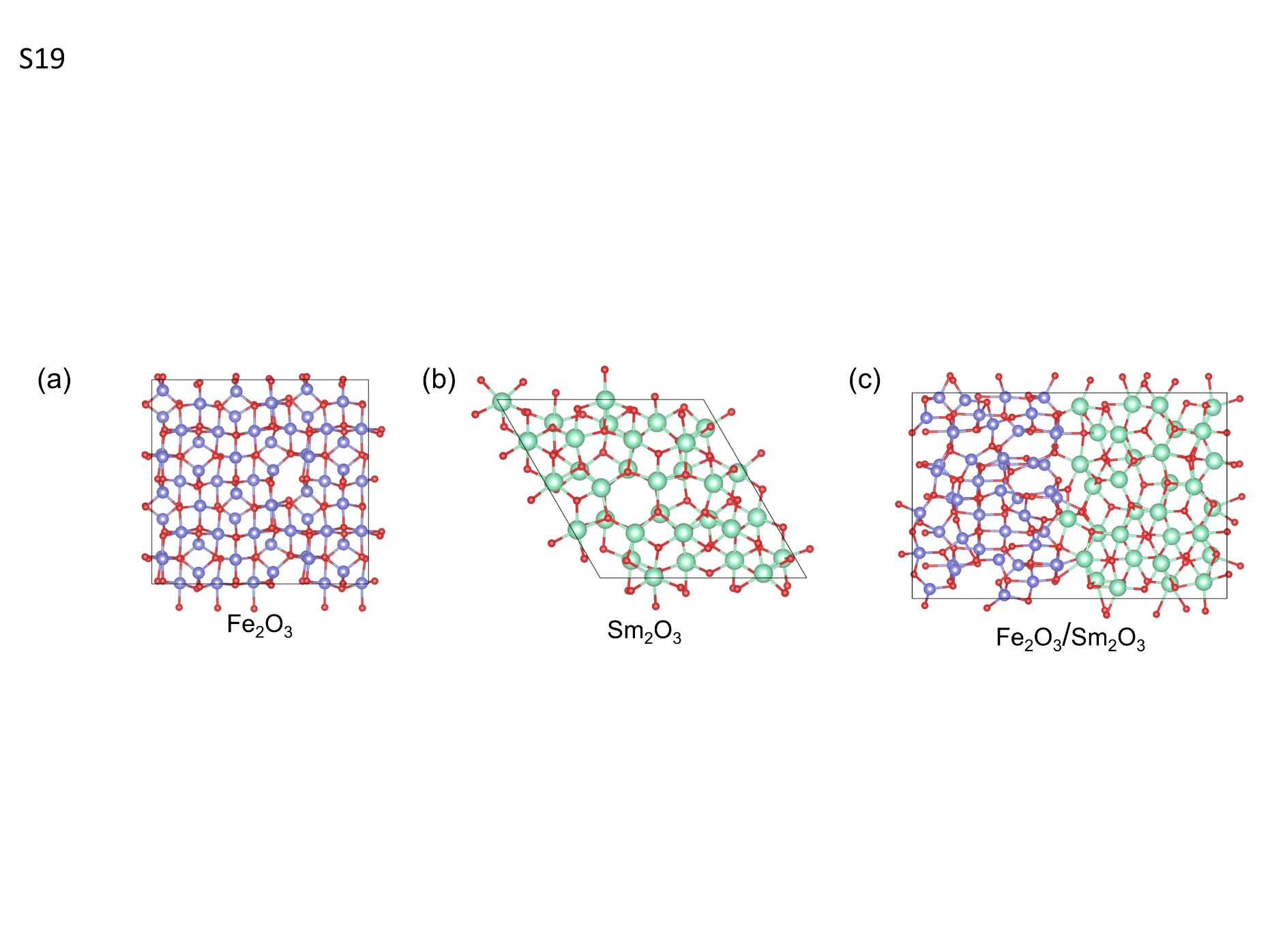


**Figure S19.** (a) The optimized structures of (a) Fe_2_O_3_ models, (b) Sm_2_O_3_ models, and (c) Fe_2_O_3_/Sm_2_O_3_ models.


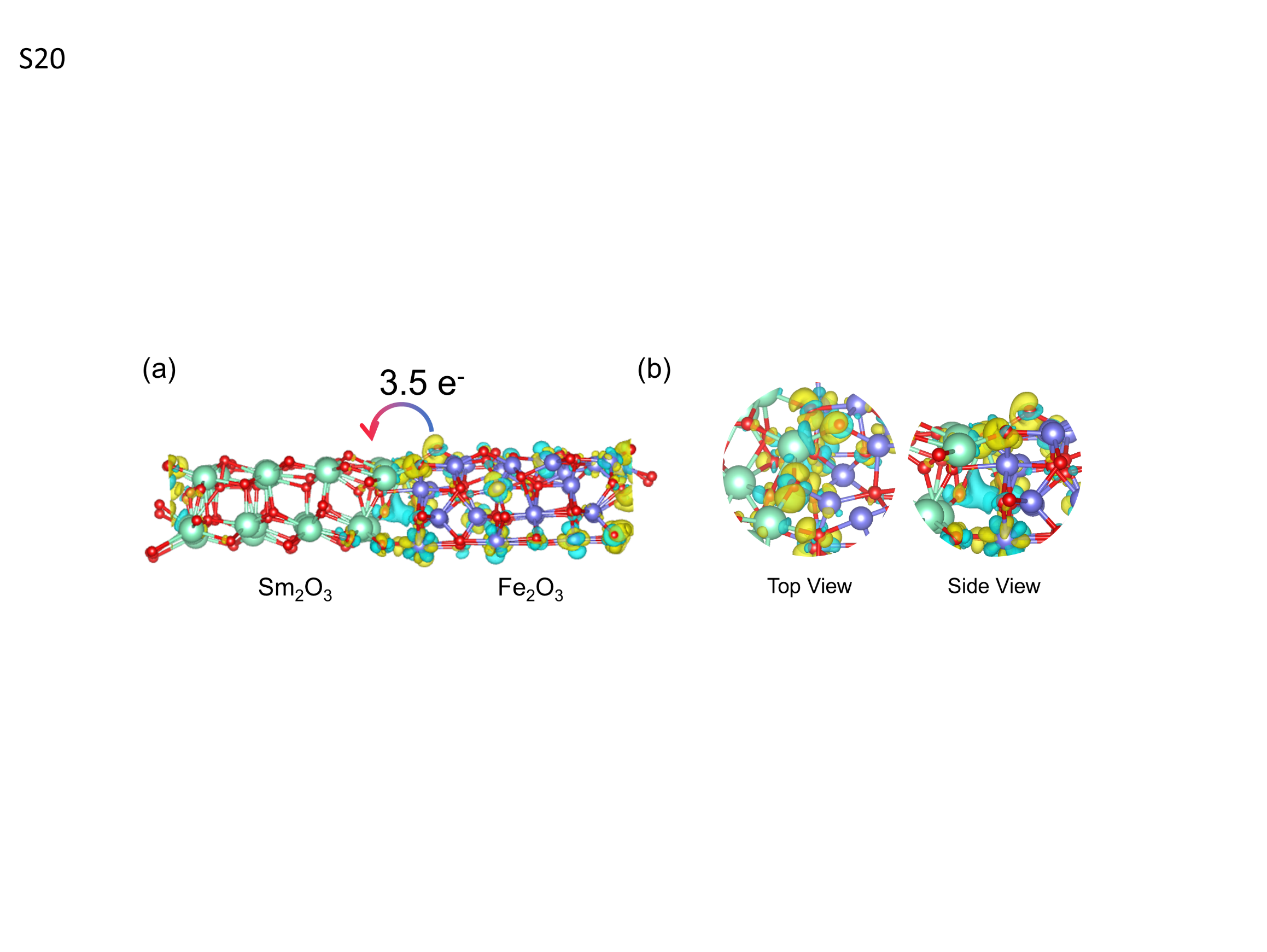


**Figure S20.** (a) Bader charge analysis, (b) differential charge analysis.


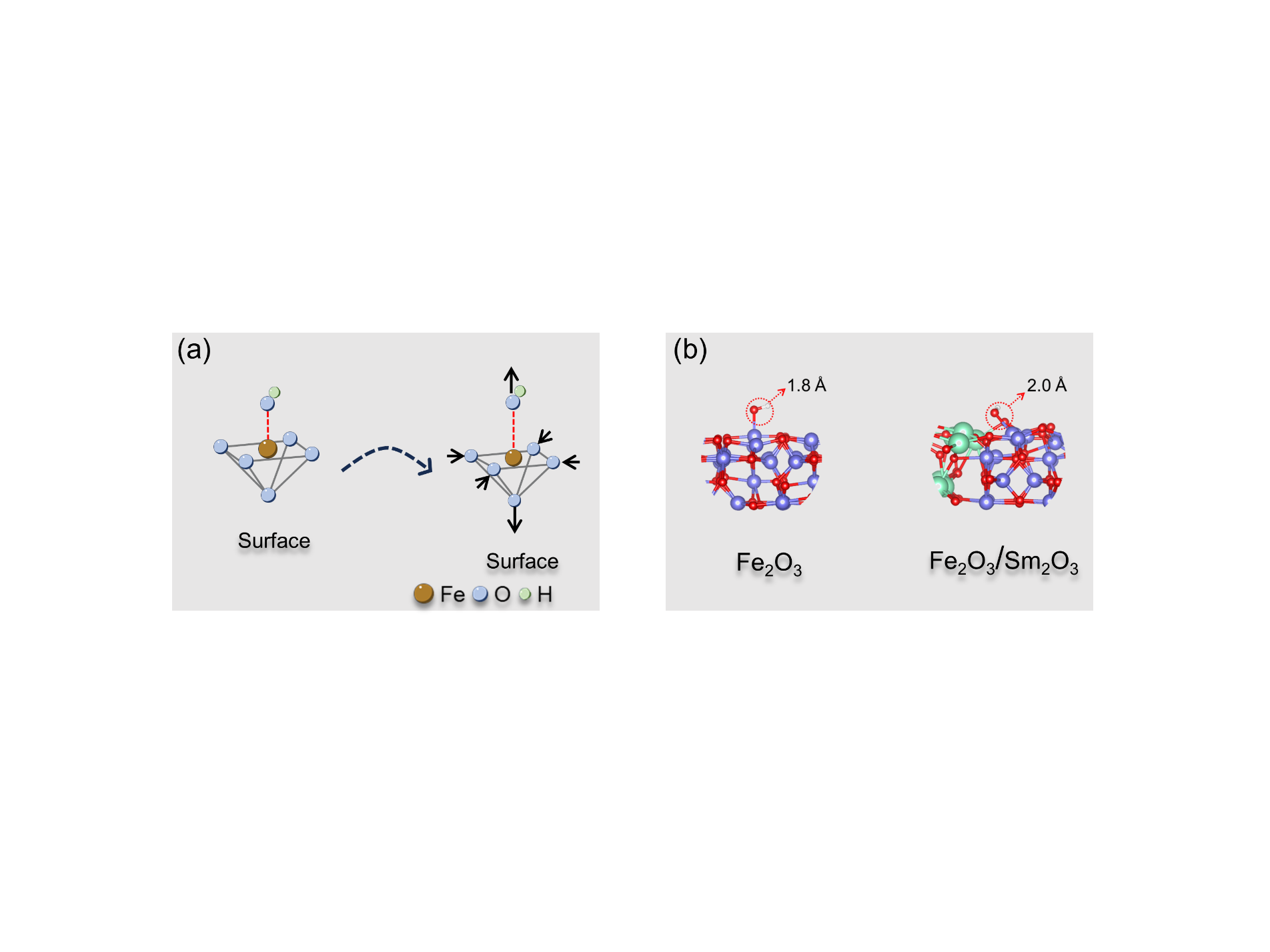


**Figure S21.** (a) schematic diagram of the Fe-OH change driven by lattice distortion on the surface, and (b) the bond length variation of Fe-OH.


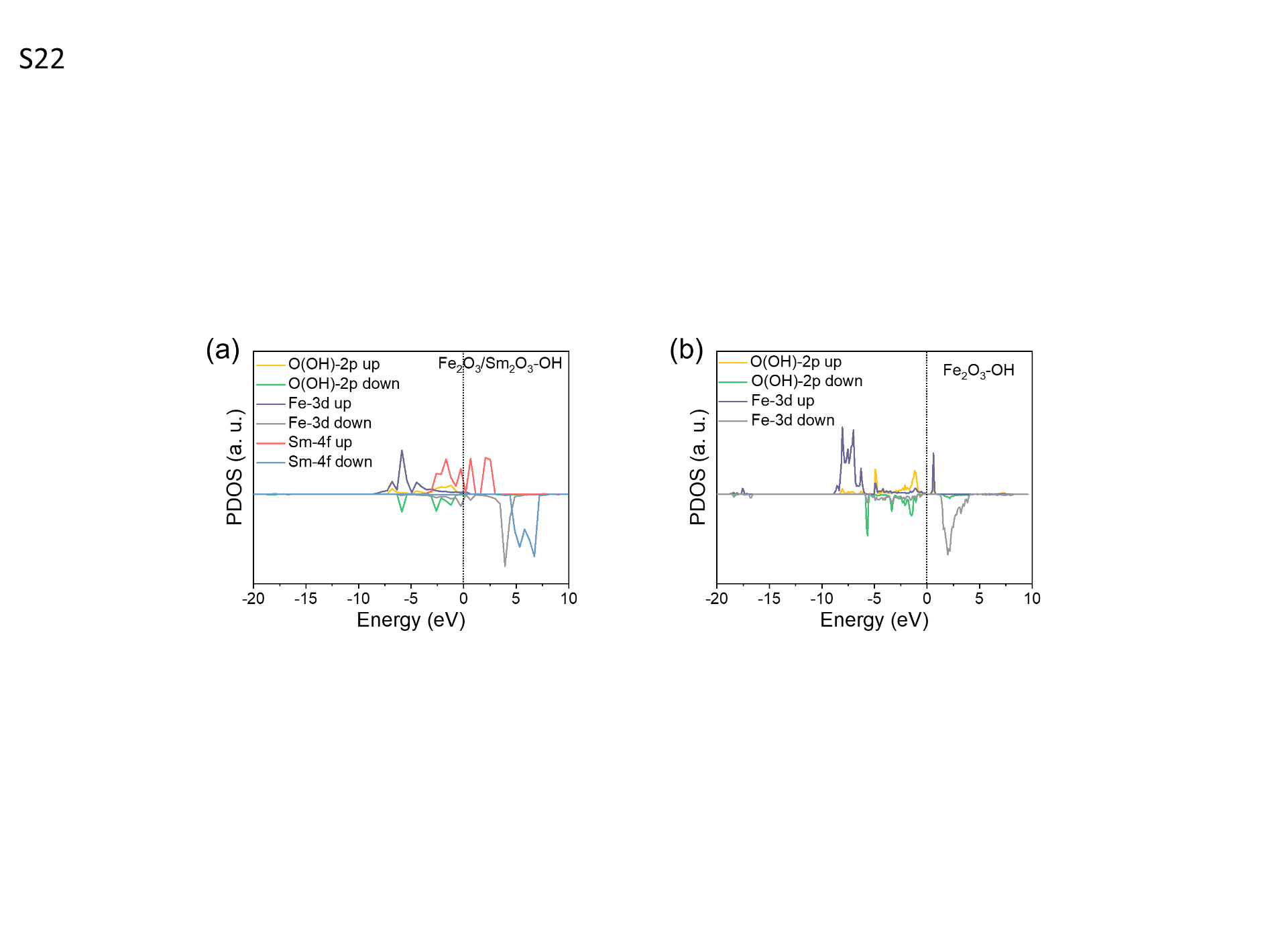


**Figure S22.** (a) PDOS of Fe_2_O_3_/Sm_2_O_3_ with OH*, (b) PDOS of Fe_2_O_3_ with OH*.


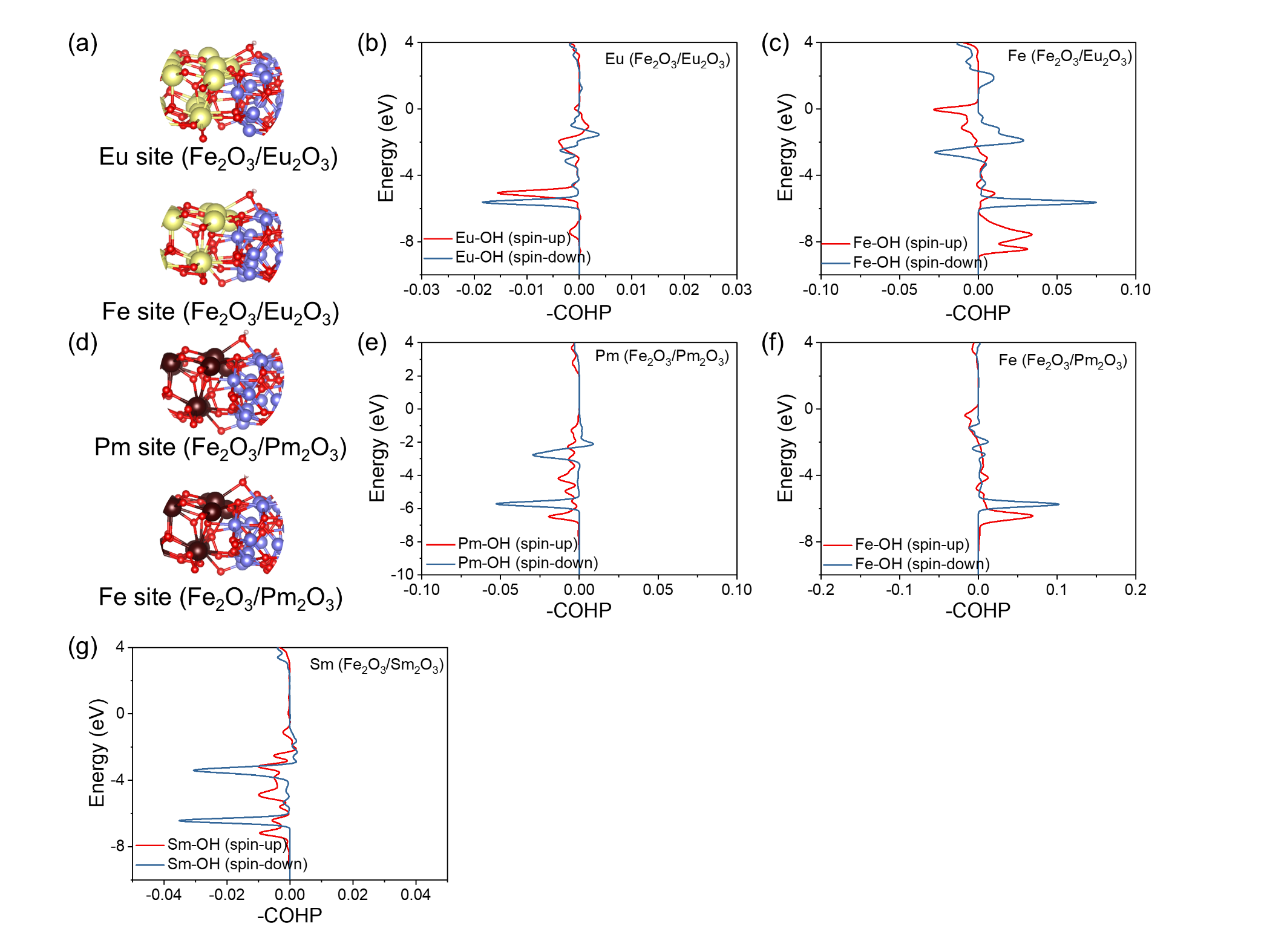


**Figure S23.** Crystal orbital Hamilton population (COHP) analysis: (a) optimized models of Fe_2_O_3_/Eu_2_O_3_, (b) Eu as the adsorption site for OH in Fe_2_O_3_/Eu_2_O_3_, (c) Fe as the adsorption site for OH in Fe_2_O_3_/Eu_2_O_3_, (d) optimized models of Fe_2_O_3_/Pm_2_O_3_, (e) Pm as the adsorption site for OH in Fe_2_O_3_/Pm_2_O_3_, (f) Fe as the adsorption site for OH in Fe_2_O_3_/Pm_2_O_3_, and (g) Sm as the adsorption site for OH in Fe_2_O_3_/Sm_2_O_3_.


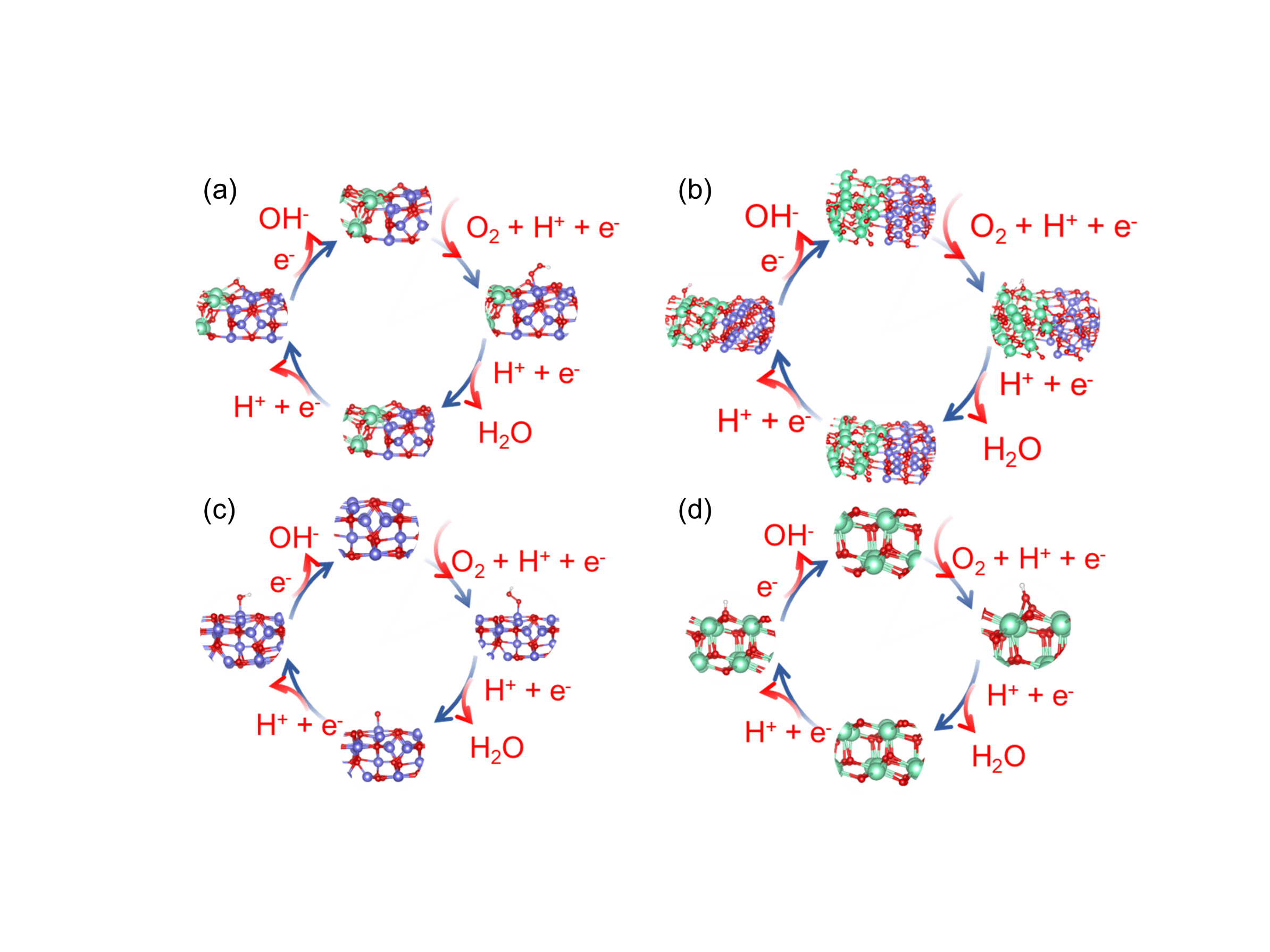


**Figure S24.** ORR reaction pathway in (a) Fe_2_O_3_/Sm_2_O_3_ with Fe as the sites, (b) Fe_2_O_3_/Sm_2_O_3_ with Sm as the sites, (c) Fe_2_O_3_, and (d) Sm_2_O_3_.


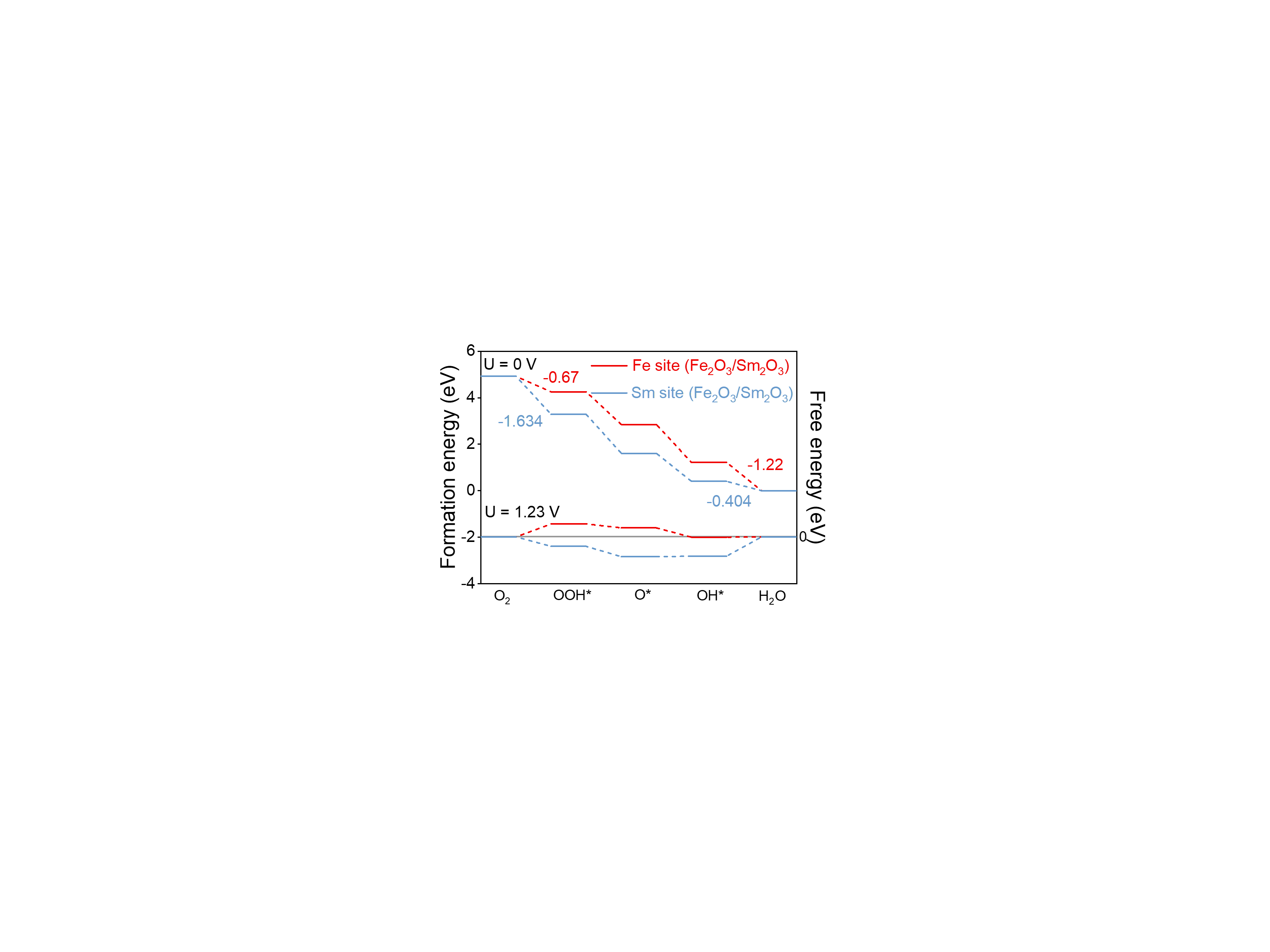


**Figure S25.** ORR free energy of Fe_2_O_3_/Sm_2_O_3_ with Fe or Sm as the sites.


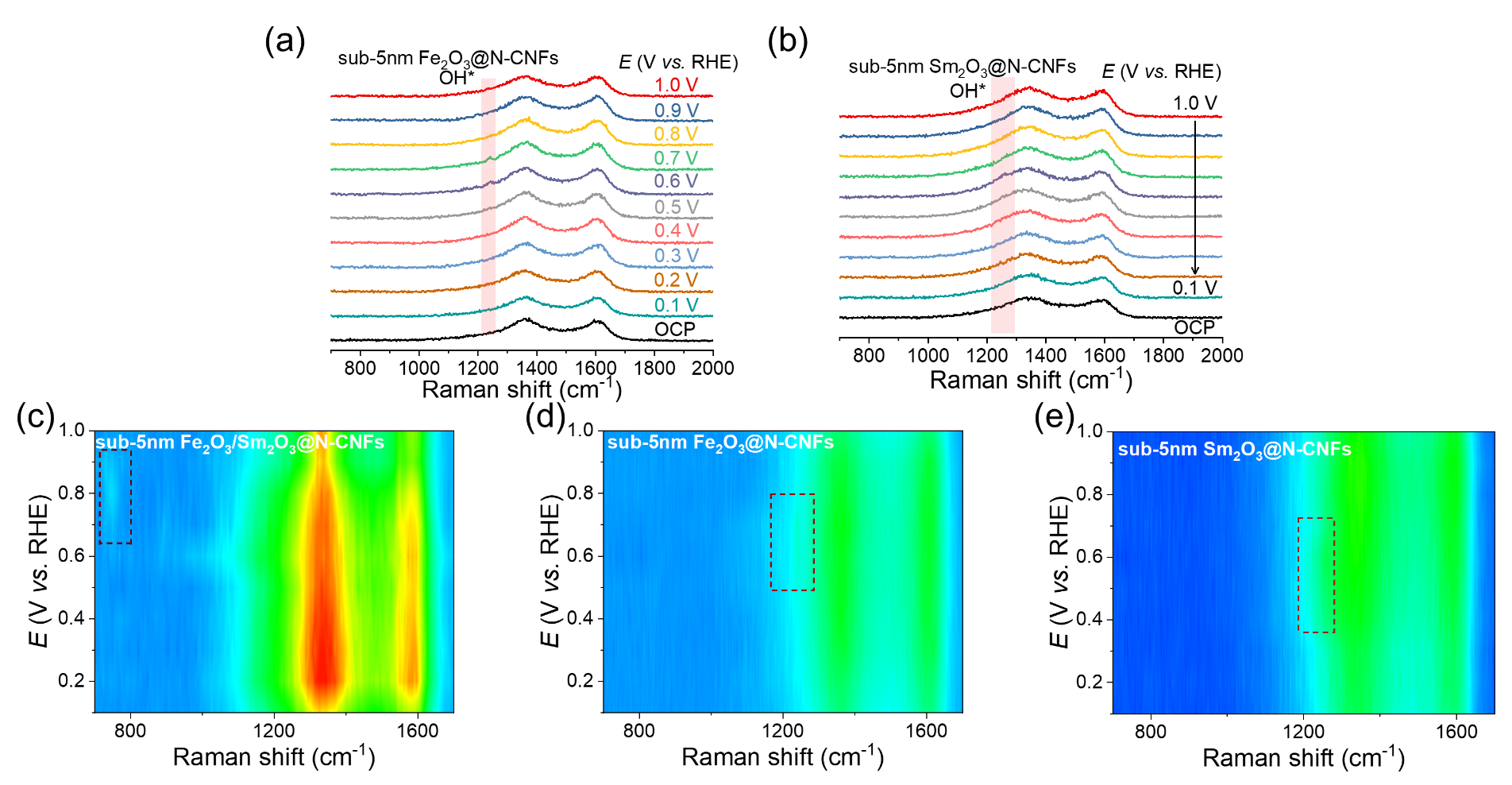


**Figure S26.** *In-situ* enhanced Raman spectra and corresponding Raman contour plot: the spectra of (a) sub-5nm Fe_2_O_3_@N-CNFs, and (b) sub-5nm Sm_2_O_3_@N-CNFs, the contour plot of (c) sub-5nm Fe_2_O_3_/Sm_2_O_3_@N-CNFs, (d) sub-5nm Fe_2_O_3_@N-CNFs, and (e) sub-5nm Sm_2_O_3_@N-CNFs.


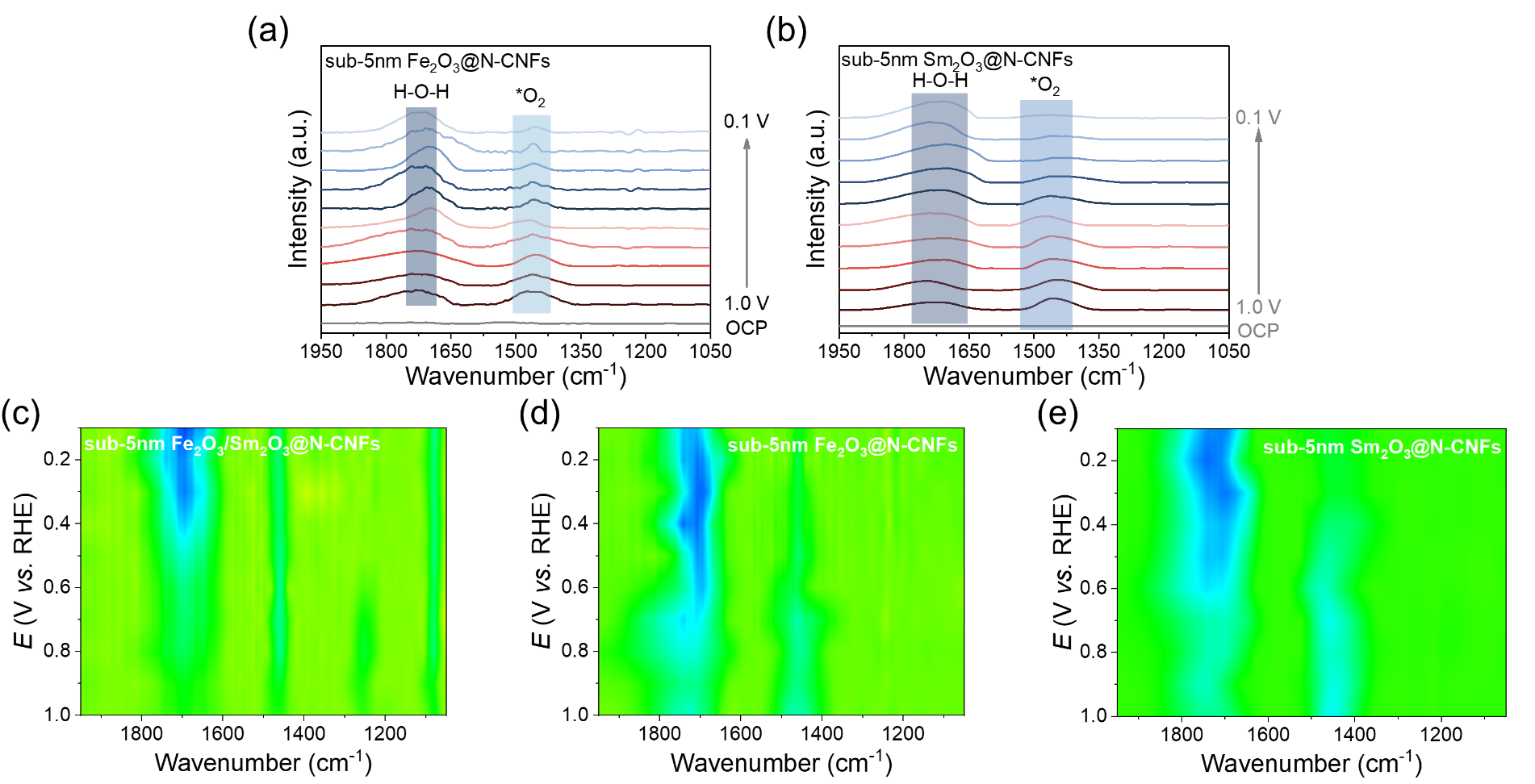


**Figure S27.** *In-situ* ATR-SEIRAS spectra and corresponding Infrared Spectroscopy contour plot: the spectra of (a) sub-5nm Fe_2_O_3_@N-CNFs, and (b) sub-5nm Sm_2_O_3_@N-CNFs, the contour plot of (c) sub-5nm Fe_2_O_3_/Sm_2_O_3_@N-CNFs, (d) sub-5nm Fe_2_O_3_@N-CNFs, and (e) sub-5nm Sm_2_O_3_@N-CNFs.


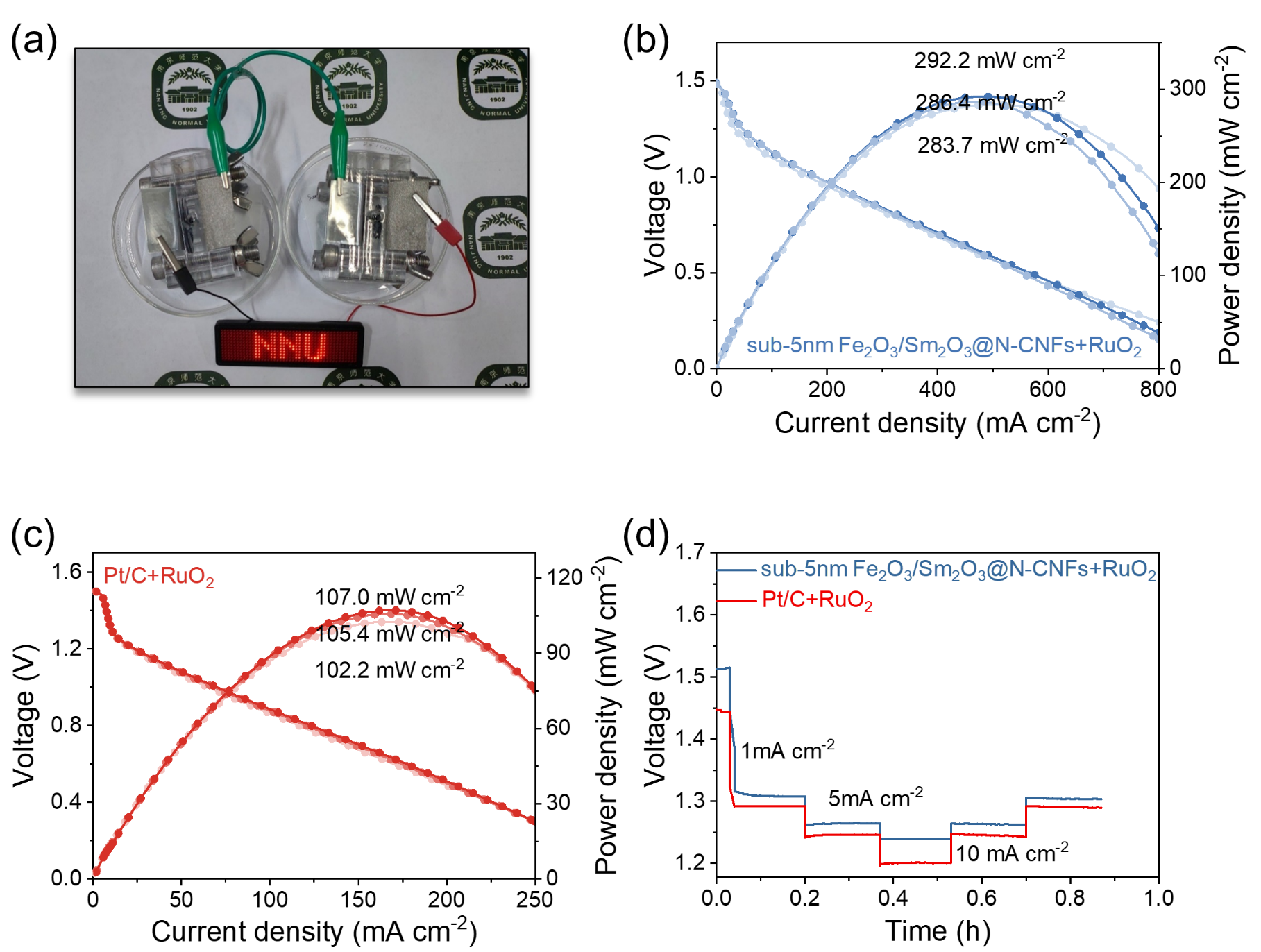


**Figure S28.** Liquid Znic-Air Battery performance test: (a) a photograph of a LED screen powered by two series-connected sub-5nm Fe_2_O_3_/Sm_2_O_3_@N-CNFs-based ZABs, (b) the discharge curves and corresponding power density curves of sub-5nm Fe_2_O_3_/Sm_2_O_3_@N-CNFs-based ZABs, (c) three discharge curve and corresponding power density curve of Pt/C-based ZAB, and (d) the galvanostatic discharge curves of sub-5nm Fe_2_O_3_/Sm_2_O_3_@N-CNFs-based ZAB and Pt/C-based ZAB at periodically changed current densities.

**Tables S1-S****5**

**Table S1.** EXAFS fitting parameters at the Fe K-edge for various samples.

| Sample | Path | CN | R (Å) | σ^2^ (🞨10^-3^ Å^2^) | E_0_ (ev) | R-factor |
| --- | --- | --- | --- | --- | --- | --- |
| Fe foil | Fe-Fe (1) | 8 | 2.47 (0.004) | 4.33  (2.75) | -0.57  (0.67) | 0.009 |
|  | Fe-Fe (2) | 6 | 2.86  (0.02) | 5.15  (5.13) |  |  |
| Sub-5nm Fe_2_O_3_@N-CNFs | Fe-O | 1.79(0.40) | 2.00  (0.02) | 5.31  (4.22) | 6.04  (2.11) | 0.019 |
|  | Fe-O-Fe | 2.35(0.54) | 3.05  (0.03) | 1.97  (1.00) |  |  |
| Sub-5nm Fe_2_O_3_/Sm_2_O_3_ N-CNFs | Fe-O | 1.21(0.24) | 1.95  (0.04) | 15.32 | 2.98  (4.42) | 0.014 |
|  | Fe-O-Sm | 0.8 (0.2) | 2.70 (0.03) | 13.25 |  |  |
| Fe_2_O_3_ | Fe-O | 6 | 2.00  (0.02) | 2.77  (0.53) | 4.38  (1.65) | 0.005 |
|  | Fe-O-Fe | 6 | 2.97  (0.03) | 2.12  (0.38) |  |  |

*^a^CN*, coordination number; *^b^R*, distance between absorber and backscatter atoms; *^c^σ*^2^, Debye-Waller factor to account for both thermal and structural disorders; *^d^ΔE*_0_, inner potential correction; *R* factor indicates the goodness of the fit. S_0_^2^ was fixed to 0.75, according to the experimental EXAFS fit of Fe foil by fixing CN as the known crystallographic value. A reasonable range of EXAFS fitting parameters: 0.600 < *Ѕ*_0_^2^ < 1.000; *CN >* 0; *σ*^2^ > 0 Å^2^; |Δ*E*_0_| < 15 eV; *R* factor < 0.02.

**Table S2.** Comparison of ORR performance of sub-5nm Fe_2_O_3_/Sm_2_O_3_@N-CNFs with some previously reported Fe-based electrocatalysts in 0.1 M KOH solution.

| **Catalyst** | ***E*_1/2_** (V_RHE_) | ***E*_onset_** (V_RHE_) | **Reference** |
| --- | --- | --- | --- |
| sub-5nm Fe_2_O_3_/Sm_2_O_3_@N-CNFs | 0.94 | 1.1 | This work |
| HPFe-N-C | 0.874 | 0.911 | *Appl. Catal. B: Environ. and Energy*, **2022**, 305, 121040. |
| Fe-N-C | 0.854 | 0.98 | *Appl. Catal. B: Environ. and Energy*, **2022**, 305, 121058. |
| Fe-N,O/G | 0.86 | 1.0 | *Energy Environ. Sci.,* ***2023***, 16, 2629-2636. |
| FeSA/N-PSCS | 0.87 | 0.976 | *Energy Storage Mater.,* **2023**, 59, 102790. |
| FeN_4_-FeNCP@MCF | 0.894 | 1.02 | *Adv. Funct. Mater.*, **2024**, 34, 2315150. |
| Fe/Meso-NC-1000 | 0.885 | 0.97 | *Adv. Mater.*, **2022**, 34, 2107291. |
| Fe-N-C SAC | 0.821 | 0.995 | *Angew. Chem. Int. Ed.* **2025**, 64, e202508674. |
| Fe_2_O_3_-MoO_3_/NG | 0.82 | 0.96 | *Chem. Eng. J.* **2021**, 410, 128358. |
| MnO_2_-Fe_2_O_3_/CNT | 0.80 | 0.92 | *Chem. Eng. J.* **2021**, 414, 128815. |
| Fe-N-DCSs | 0.90 | 1.00 | *Angew. Chem. Int. Ed.* **2022**, 61, e202208238. |
| FePC-NH_2_/HCB-800 | 0.84 | 0.98 | *J. Energy Chem.* **2019**, 28, 73-78. |
| Fe-ACSA@NC | 0.90 | 1.03 | *Angew. Chem. Int. Ed.*, **2022**, 61, e202116068. |

**Table S3.** The detailed fitting results of all catalysts include surface charge transfer resistance (*R*_ct_), error and error percentage.

| **Catalyst** | ***R*_ct_ (Ω)** | **Error** | **Error %** |
| --- | --- | --- | --- |
| sub-5nm Fe_2_O_3_/Sm_2_O_3_@N-CNFs | 64.08 | 0.64237 | 1.0025 |
| Fe_2_O_3_/Sm_2_O_3_@N-CNFs | 78.77 | 0.30039 | 0.38135 |
| sub-5nm Fe_2_O_3_@N-CNFs | 78.97 | 1.0642 | 1.3476 |
| Fe_2_O_3_@N-CNFs | 81.07 | 1.622 | 2.0007 |
| sub-5nm Sm_2_O_3_@N-CNFs | 82.26 | 0.43641 | 0.53053 |
| Sm_2_O_3_@N-CNFs | 100.2 | 1.4941 | 1.4911 |

**Table S4.** The element content obtained through Inductively Coupled Plasma (ICP) testing.

| **Sample** | **Fe (wt.%)** | **Sm (wt.%)** |
| --- | --- | --- |
| sub-5nm Fe_2_O_3_/Sm_2_O_3_@N-CNFs | 9.34 | 10.52 |
| post-tested sub-5nm Fe_2_O_3_/Sm_2_O_3_@N-CNFs | 9.20 | 9.98 |

**Table S5.** Comparison of ZABs performance of sub-5nm Fe_2_O_3_/Sm_2_O_3_@N-CNFs with some previously reported Fe-based electrocatalysts in 6 M KOH + 0.2 M ZnCl_2_ solution.

| **Catalysts** | **Power density (mW cm^-2^)** | **Cycle life (h)** | **Reference** |
| --- | --- | --- | --- |
| sub-5nm Fe_2_O_3_/Sm_2_O_3_@N-CNFs | 287.4 | 1367 | This work |
| SiO_2_/FeNSiC | 195.9 | 200 | *Nat. Commun.* **2025**, 16, 10178. |
| Fe_1_Co_2_/NC | 282.7 | 520 | *Nat. Commun.* **2025**, 16, 11691. |
| Ru_SA_-NiFe LDH HE | 299.2 | 2400 | *Nat. Commun.* **2024**, 15, 9616. |
| Co-N-Mn/NC | 271 | 200 | *Nat. Commun.* **2025**, 16, 5158. |
| CoCo-BiSalphen@KB | 215.7 | 500 | *Nat. Commun.* **2025**, 16, 921. |
| CuNa-CF | 264.2 | 5000 | *Nat. Commun.* **2024**, 15, 8365. |
| CA-Fe@BC | 227.6 | 650 | *Nat. Commun.* **2025**, 16, 2920. |
| Fe_2_NC-Cl | 231 | 2400 | *J. Am. Chem. Soc.* **2026**, 148, 5, 5167-5178 |
| P/S-pC | 204.9 | 190 | *J. Am. Chem. Soc.* **2025**, 147, 45, 41472-41480 |
| FeSn-C_2_N | 265.5 | 320 | *J. Am. Chem. Soc.* **2024**, 146, 31, 21357-21366 |
| FeDy-DAC | 231.8 | 2500 | *Adv. Mater.* **2026**, e20359. |
| FePc/cGDY/CNT | 191.3 | / | *Adv. Mater.* **2026**, e22464. |
| InPPc/v-CNTs | 270 | 865 | *Adv. Mater.* **2026***,* 38, 9, e22225. |
| PtCo@NPC | 238 | 840 | *Angew. Chem. Int. Ed..* **2026***,* 65, e24805. |
| FeSnNC | 262 | 1100 | *Angew. Chem. Int. Ed..* **2026,** 65, e24265. |
| g-CAN-Pc | 178 | 80 | *Angew. Chem. Int. Ed.* **2025**, 64, e16530, |
| Fe_1_V_1_-NC | 237 | 400 | *Angew. Chem. Int. Ed.* **2025***,* 64, e202514542. |
| CoPt_3_@NC-NFs | 184.8 | / | *Energy Environ. Sci.* **2026.** |
| Fe_1_Co_1_-N-C | 234 | 3600 | *Energy Environ. Sci.,* **2025***,*18, 4949-4961. |

**References**

[1] Kresse, G., Furthmüller, *J. Comput. Mater. Sci.* 1996, 6, 15-50.

[2] Kresse, G., Furthmüller, *J. Phys. Rev. B* 1996, 54, 11169-11186.

[3] Perdew, J. P., Burke, K.; *Phys. Rev. Lett.* 1996, 77, 3865-3868.

[4] Kresse, G., Joubert, D. *Phys. Rev. B* 1999, 59, 1758-1775.

[5] Blöchl, P. E. Projector Augmented-Wave Method. *Phys. Rev. B* 1994, 50, 17953-17979.

[6] Grimme, S., Antony, J.; Ehrlich, S.; Krieg, H. *J. Chem. Phys.* 2010, 132, 154104.

[7] Obodo, K. O., Gebreyesus, G., Ouma, C. N. M., Obodo, J. T., Ezeonu, S. O., Raif, D. P., Bouhafs, B. *RSC Adv.*, 2020,10, 15670-15676.

[8] Kirchner-Hall, N. E., Zhao, W., Xiong, Y., Timrov, I., Dabo, I. *Appl. Sci.*, 2021, 11(5), 2395.

[9] Nørskov, J. K., Rossmeisl, J., Logadottir, A., et al. *J. Phys. Chem.* B 2004, 108, 17886-17892.

[10] Zhang, D., Wang, Z., Liu, F., Yi, P., Peng L., Chen Y., Wei L., Li, H. *J. Am. Chem. Soc.* 2024, 146 (5), 3210-3219.

[11] Dickens, C. F., Kirk, C., Nørskov, J. K. *J. Phys. Chem. C* 2019, 123 (31), 18960-18977.

[12] Hansen, H. A., Viswanathan, V., Nørskov, J. K. *J. Phys. Chem. C* 2014, 118 (13), 6706-6718.

[13] Kelly, S. R., Kirk, C., Chan, K., Nørskov, J. K. *J. Phys. Chem. C* 2020, 124 (27), 14581-14591.

[14] Li, H., Kelly, S., Guevarra, D., Wang, Z., Wang, Y., Haber, J. A., Anand, M., Gunasooriya, G. T. K. K., Abraham, C. S., Vijay, S., Gregoire, J. M., Nørskov, J. K. *Nat. Catal*. 2021, 4, 463-468.

[15] Zhang, D., She, F., Chen, J., Wei, L., Li, H. *J. Am. Chem. Soc.* 2025, 147, 7, 6076-6086.

[16] Li, M., Wang, X. *J. Am. Chem. Soc.* 2025, 147, 1732-1739.

[17] Li, M., Yang, J., Li, S., Deng, L., Zhao, S., Li, L., Hung, S., Xing, G., Wang, T., Liang, Y., Ren, J., Wu, Y., Peng, S. *J. Am. Chem. Soc.* 2025, 147, 45680-45690.
